# Evolving the Promiscuity of *Elizabethkingia meningoseptica* Oleate Hydratase for the Regio‐ and Stereoselective Hydration of Oleic Acid Derivatives

**DOI:** 10.1002/anie.201901462

**Published:** 2019-04-17

**Authors:** Matthias Engleder, Gernot A. Strohmeier, Hansjörg Weber, Georg Steinkellner, Erich Leitner, Monika Müller, Daniel Mink, Martin Schürmann, Karl Gruber, Harald Pichler

**Affiliations:** ^1^ Institute of Molecular Biotechnology Graz University of Technology, NAWI Graz, BioTechMed Graz Petersgasse 14 8010 Graz Austria; ^2^ ACIB GmbH—Austrian Centre of Industrial Biotechnology Petersgasse 14 8010 Graz Austria; ^3^ Institute of Organic Chemistry Graz University of Technology, NAWI Graz Stremayrgasse 9 8010 Graz Austria; ^4^ Innophore GmbH Am Eisernen Tor 3 8010 Graz Austria; ^5^ Institute of Analytical Chemistry and Food Chemistry Graz University of Technology, NAWI Graz Stremayrgasse 9 8010 Graz Austria; ^6^ InnoSyn B.V. Urmonderbaan 22 6167 RD Geleen The Netherlands; ^7^ Institute of Molecular Biosciences University of Graz, NAWI Graz, BioTechMed Graz Humboldtstrasse 50 8010 Graz Austria

**Keywords:** enzyme catalysis, fatty acid hydratase, hydrolyases, protein engineering, substrate promiscuity

## Abstract

The addition of water to non‐activated carbon–carbon double bonds catalyzed by fatty acid hydratases (FAHYs) allows for highly regio‐ and stereoselective oxyfunctionalization of renewable oil feedstock. So far, the applicability of FAHYs has been limited to free fatty acids, mainly owing to the requirement of a carboxylate function for substrate recognition and binding. Herein, we describe for the first time the hydration of oleic acid (OA) derivatives lacking this free carboxylate by the oleate hydratase from *Elizabethkingia meningoseptica* (OhyA). Molecular docking of OA to the OhyA 3D‐structure and a sequence alignment uncovered conserved amino acid residues at the entrance of the substrate channel as target positions for enzyme engineering. Exchange of selected amino acids gave rise to OhyA variants which showed up to an 18‐fold improved conversion of OA derivatives, while retaining the excellent regio‐ and stereoselectivity in the olefin hydration reaction.

Fatty acid hydratases (FAHYs; EC 4.2.1.X) catalyze the formation of medium‐ and long‐chain hydroxylated fatty acids by addition of water to isolated carbon–carbon double bonds of free mono‐ or polyunsaturated fatty acids.^1^, ^2^, ^3^, ^4^, ^5^ As such, they provide access to secondary and tertiary alcohols, which makes them valuable tools for the production of a variety of chemicals, including flavor additives,^6^, ^7^, ^8^ surfactants, lubricants, and precursors in polymer chemistry.^9^, ^10^, ^11^, ^12^, ^13^ The use of FAHYs in synthesis promises advantages relating to their exquisite regio‐ and stereoselectivity, which permits reactions that are not possible with unselective acid‐catalyzed chemical hydrations.^14^, ^15^, ^16^, ^17^, ^18^, ^19^, ^20^, ^21^ Moreover, FAHYs are not impeded by the common challenges of other enzymes applied in hydroxylation reactions, which are often limited by low expression, poor stability and/or complex electron‐transport chains for nucleotide cofactor regeneration.^1^, ^2^, ^20^, ^21^, ^22^, ^23^, ^24^, ^25^, ^26^, ^27^ This has led to a growing attention for this class of hydrolyases for establishing new routes in sustainable organic syntheses. Most known FAHYs are highly regioselective in hydrating either the *cis*‐9 or *cis*‐12 double bond(s) of unsaturated fatty acids.^5^, ^28^, ^29^, ^30^, ^31^, ^32^, ^33^ The only enzyme described so far showing a broader substrate scope is a FAHY isolated from *Lactobacillus acidophilus* NTV001 (FA‐HY1). It catalyzes the hydration of *cis*‐9, *cis*‐12, *cis*‐13, *cis*‐14 or *cis*‐15 double bonds of free fatty acids with different chain lengths and degrees of saturation, but not of the esters thereof,^34^ which highlights limitations of the substrate scope. In contrast, the collection of 2046 putative FAHY sequences in a hydratase engineering database (HyED) reflects a hitherto largely unexplored potential of this enzyme class to possibly catalyze reactions that are unattainable with current synthetic methodologies.^35^

The most thoroughly characterized FAHY to date is the oleate hydratase from *Elizabethkingia meningoseptica* (OhyA, EC 4.2.1.53).^36^, ^37^, ^38^ This enzyme catalyzes the regio‐ and stereoselective hydration of oleic acid (OA), yielding (*R*)‐10‐hydroxy stearic acid with an excellent *ee* of ≥98 % and without the need for co‐factor recycling.^14^, ^21^, ^37^, ^38^ Currently, it is one of only four FAHYs with a resolved X‐ray crystal structure, and the only one analyzed in complex with the essential, non‐covalently bound FAD cofactor.^33^, ^38^, ^39^, ^40^ By comparing the surrounding of the FAD binding site of OhyA with other FAHY structures lacking a co‐crystallized cofactor, a structural role of the flavin for correct arrangement of the active site was inferred.^5^, ^32^, ^38^, ^39^, ^40^

Despite the progress in research, the applicability of FAHYs in an industrial setting is still limited, which can be mostly attributed to their strict substrate requirements. All published work demonstrated that a minimum distance of 7 carbon atoms between the essential carboxylate and the double bond in *cis*‐configuration, along with a minimum fatty acid chain length of 11 carbon atoms are mandatory for conversion.^5^, ^28^, ^35^, ^42^ Hydration at a terminal carbon atom in unsaturated fatty acids has also not been described so far, as a partial positive charge at the primary carbon atom would not be favored (Markovnikov's rule).^38^, ^43^ Several structural requirements for substrates to undergo OhyA‐catalyzed hydrations were recently bypassed by Demming and co‐workers. They succeeded in converting 1‐decene into (*S*)‐2‐decanol in a reaction using whole cells of *Escherichia coli* containing OhyA.^43^ Strikingly, varying an amino acid position (Ala 248) located at the end of the alkyl binding pocket to larger hydrophobic or aromatic residues allowed for improved hydration of various short‐chain 1‐alkenes, and even permitted the conversion of six out of 23 tested functionalized and internal alkenes.^44^ The activity of their catalytic system was, however, strongly dependent on the addition of a carboxylic acid as decoy molecule to arrange the binding of a non‐covalently joined “pseudo‐fatty acid motif”, which is presumably formed in situ by simultaneous alignment of the decoy carboxylic acid and the terminal olefin to be hydrated.^43^, ^44^, ^45^ These and our own results inspired us to further explore the promiscuity of oleate hydratases based on applying OhyA as the model enzyme. Driven by our primary focus on the role of the head group of a fatty acid in substrate recognition, we performed conversion assays with nine different OA derivatives (Scheme 1, entries **1 a**–**1 j**). The rationale behind our model substrate selection was to examine the acceptance of head groups with different physiochemical properties regarding size and hydrophobicity, as well as charge and polarization, that is, amine, amide, hydroxamic acid, alcohol, and short‐chain ester groups. Additionally, we preferably selected OA analogues that were already commonly used in various applications and which could be easily obtained in more than 90 % purity through purification of commercially available materials or via synthesis from appropriate precursors.

**Scheme 1:**
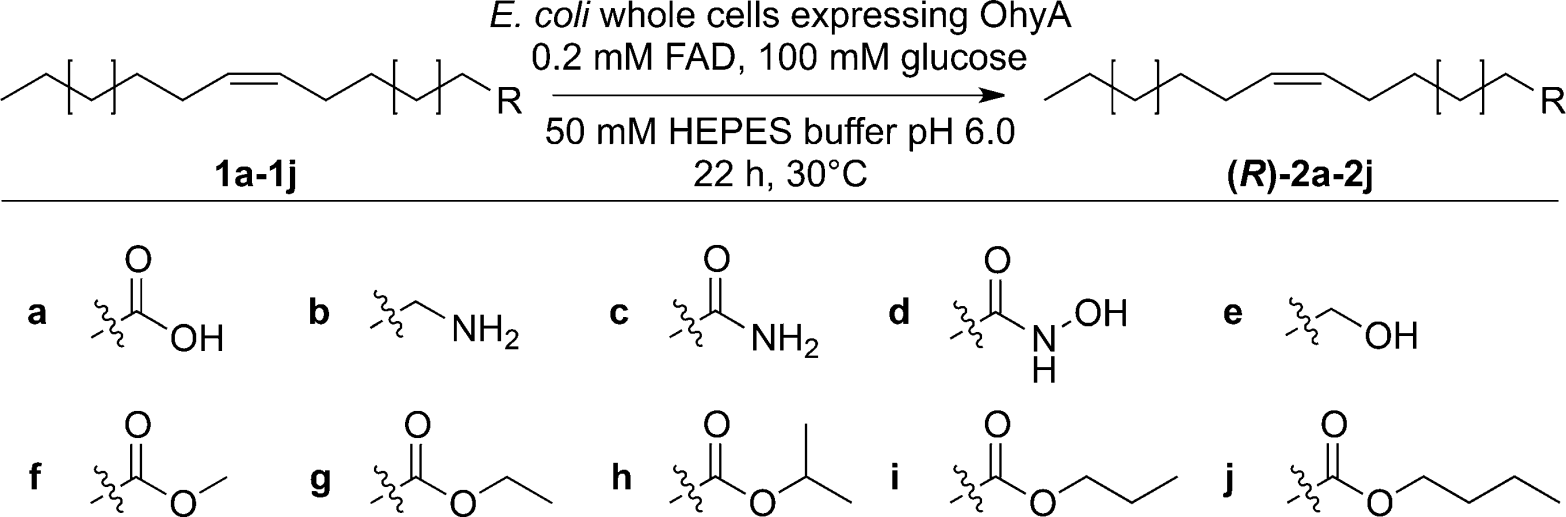
Regio‐ and stereoselective hydration of oleic acid (OA) and OA derivatives by *E. meningoseptica* oleate hydratase (OhyA). A whole‐cell *E. coli* biocatalyst harboring OhyA was used in the biotransformation assays.

At the beginning of our investigations, we incubated cell‐free lysate containing recombinantly expressed OhyA with the series of non‐natural substrates, but did not detect compound peaks corresponding to hydrated products in GC‐MS analyses. Knowing that the previously reported enzyme activity for the hydration of *cis*‐9‐undecenoic acid was also found low with cell‐free lysate, but could be greatly enhanced by using *E. coli* whole cells as enzyme source, we decided to repeat the biotransformations in a whole cell system and judge again the enzyme's activity on alternative substrates.^35^, ^43^

Remarkably, after 96 h of biotransformation, product from most OA derivatives was indeed detected in assays with *E. coli* cells harboring recombinant OhyA. As indicated by characteristic mass fragmentation patterns in GC‐MS, non‐physiological substrates **1 c**–**1 g**, as well as **1 i** were converted into the respective 10‐hydroxy compounds **2 c**–**2 g** and **2 i** (Figure S1–S7 and S9 in the Supporting Information). Control reactions using an OhyA‐free *E. coli* strain showed no hydration of any substrate and no side reactions (Figure S1–S10). Products from biotransformations were extracted, purified and verified by NMR spectroscopy (Figure S11–S22, S25 and S26). We were pleased to see that the (*R*)‐alcohols of the hydrated fatty acid derivatives were obtained with *ee* values ≥95 %. This confirmed that the excellent stereoselectivities remained unchanged in OhyA‐catalyzed hydration reactions when using the selected non‐natural substrates. Whereas the OhyA‐mediated conversion of short‐chain alkenes described by Demming et al.^43^ required the use of a carboxylic acid as decoy molecule, hydration of OA derivatives catalyzed by the same enzyme was independent of any co‐substrate and worked in the absence of a free carboxylic acid head group. Our study, therefore, presents the first hydration of fatty acid derivatives devoid of a free carboxylate by the action of an oleate hydratase, and, thus, breaks the notion of the essential carboxylic acid function required in substrate recognition by FAHYs.^5^, ^22^, ^28^, ^34^, ^42^, ^43^ In the tested reaction setups, only OA derivatives **1 b**, **1 h**, and **1 j** were not converted. We attribute the non‐conversion of **1 b** to the distinctly different properties of its amine head group, which exists as a cationic ammonium salt under our reaction conditions and, presumably, cannot bind to the enzyme's active site in a proper way to enable hydration. In case of **1 h** and **1 j**, the hydrophobicity of the substrates combined with the steric constraints of the entrance channel most likely prohibited their conversion.

Even though we observed hydration of six out of nine tested non‐natural substrates, the enzyme was notably more active on OA (**1 a**). We considered this as a challenge for the development of a strategy to improve enzyme activity on fatty acid derivatives lacking a free carboxylate head group. To start this task, we referred to our previous study,^38^ in which we proposed that several amino acid positions in OhyA (Gln 265, Thr 436, Asn 438, and His 442) close to the carboxylate of a docked OA molecule mediate substrate binding. This was supported by an analysis of the sequences compiled in the HyED, which showed that the identified positions were conserved among most members of the database (Figure S29).^35^ Thus, we selected OhyA residues Gln 265, Thr 436, Asn 438 and His 442 for rational amino acid exchanges. Ultimately, our goal was to stabilize the interaction between the enzyme and OA derivatives by introducing amino acid side chains that promote binding of the different head groups (Table 1). In view of the advantages provided by a whole‐cell system compared with cell‐free lysates or purified enzymes, such as the higher operational stability, easier handling and the avoidance of tedious purification steps,^43^, ^44^ we decided to employ recombinant *E. coli* whole‐cell biocatalysts throughout our work.

**Table 1:** Conserved amino acid (AA) positions in the substrate binding region of OhyA selected for the site‐directed mutagenesis study. Amino acids are colored according to the physiochemical properties of their side chains: Hydrophobic: Black. Positively charged: Green. Negatively charged: Red. Positively polarized, uncharged: Orange. Negatively polarized, uncharged: Blue. Aromatic: Gray.

AA position	AA in wild type enzyme	Exchanged for
265	Gln	Ala/Glu/Lys/Ser
436	Thr	Ala/Asn/Asp/Lys
438	Asn	Ala/Arg/Asp/Lys/Ser
442	His	Ala/Asn/Asp/Gln/Glu/Tyr

To unravel the involved complexity of oleyl amine (**1 b**) and oleamide (**1 c**) binding, we introduced side chains providing hydroxy and carboxy functions, while the acceptance of *N*‐hydroxy oleamide (**1 d**) and oleyl alcohol (**1 e**) was challenged by selecting amide, amine and guanidinium groups, as well as alanines. Binding of OA esters (**1 f**–**1 j**) was fostered by positioning alanine residues at the apex of the substrate binding channel to open up and increase the hydrophobicity at its entrance. In total, we created 33 enzyme variants (19 single, 10 double, three triple, and one quadruple variant) as authoritative subset to represent various tactics in modifying the substrate recognition site. After generation of the variants by site‐directed mutagenesis, we expressed them in *E. coli* BL21 Star (DE3; see Table S2 for a comprehensive list of all variants). The analysis of cell‐free *E. coli* lysates containing recombinant enzymes by SDS‐PAGE confirmed soluble and, overall, largely uniform expression of the wild type enzyme and most variants (Figure S30). Only OhyA His442Asp was prone to being expressed in insoluble fashion, while the OhyA Gln265Ala/Thr436Ala variant was not expressed at all and, thus, not employed in activity assays.

We tested the activity of each of the remaining 31 variants for the physiological substrate (**1 a**) and, consequently, assessed the impact of the amino acid exchanges on the conversion of OA derivatives (**1 b**–**1 j**). While the highest hydration activity for **1 a** was still obtained with the wild‐type enzyme (Figure S31), we were intrigued by the fact that several substitutions affected the conversion of non‐natural substrates **1 c**–**1 j** (Figure S33–S40), confirming our initial selection of important residues for substrate binding (Figure 1 a–f and Figure S41). We noticed that the impact of each amino acid exchange on enzyme activity was strongly dependent on its location, which indicated a position specific effect of each variation. We would also like to emphasize that the highly *R*‐selective hydration (*ee* ≥95 %) was not affected for any of the tested substrates, confirming the broad utility of this approach for the challenging asymmetric hydration of non‐activated carbon–carbon double bonds (Figure S42–S49).

**Figure 1:**
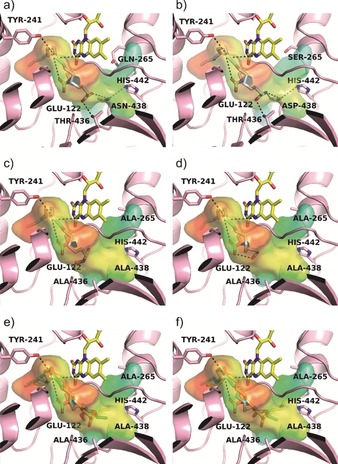
Docking of oleic acid (**1 a**) and oleic acid derivatives **1 c**–**1 j** to the OhyA 3D structure after in silico mutagenesis of conserved substrate binding residues 265, 436, 438, and 442. a)–f) show the enzyme variant–substrate combination that resulted in the best conversion to provide structure‐based evidence for the impact of the amino acid exchanges. The hydrophobicity of the enzyme cavity is represented by a color gradient from red (hydrophobic) to blue (hydrophilic). Co‐crystallized FAD (yellow) and the substrates in the best docking mode are shown in stick representation. Substrate binding residues and catalytic Glu 122 and Tyr 241 are highlighted. a) Docking of (**1 a**) to the 3D structure of OhyA wild type enzyme. b) Docking of oleamide (**1 c**) to OhyA Q265S/N438D. c) Docking of *N*‐hydroxy oleamide (**1 d**) to OhyA Q265A/T436A/N438A. d) Docking of oleyl alcohol (**1 e**) to OhyA Q265A/T436A/N438A. e) Docking of methyl (**1 f**), ethyl (**1 g**) and *n*‐propyl (**1 i**) oleate to OhyA Q265A/T436A/N438A. f) Docking of *i*‐propyl (**1 h**) and *n*‐butyl (**1 j**) oleate to OhyA Q265A/T436A/N438A.

Hydration of **1 c** was improved by substituting the amide side chains of Gln 265 and Asn 438 for hydroxy or carboxy functions. Most prominently, we found that combining the substitutions in OhyA variant Gln265Ser/Asn438Asp resulted in a 2.7‐fold increase in formation of **2 c** (Figure S50). We explain this higher activity of the double variant towards **1 c** with a more favorable interaction between its head group compared to wild‐type‐like amino acids (Figure 1 b). In case of **1 d**, we obtained a 3.6‐fold increase upon exchanging multiple substrate binding residues for alanines in OhyA Gln265Ala/Thr436Ala/Asn438Ala (Figure S51), whereas all enzyme variants harboring single amino acid exchanges and most variants with two substitutions showed either a substantially lower or wild type‐like activity. We hypothesize that the extended space in the substrate binding region provided by the triple alanine variant resulted in a better acceptance of the more sterically hindered **1 d** (Figure 1 c). Hydration of **1 e** was also 2‐fold higher with the triple alanine variant OhyA Gln265Ala/Thr436Ala/Asn438Ala (Figure S52). This observation was somewhat counterintuitive to our initial understanding of substrate recognition by OhyA, since we were expecting that a larger opening of the substrate binding channel would tend to destabilize the binding of the smaller head group of **1 e**. We therefore mainly attribute the higher conversion of **1 e** to the introduction of a more hydrophobic environment, resulting in a better compatibility of substrate and the binding region (Figure 1 d).

The activity of OhyA on short‐chain OA esters **1 f**–**1 j** could be increased remarkably with variants harboring alanine residues at the substrate entrance channel. While several single and double alanine substitutions moderately improved ester conversions, the triple alanine variant OhyA Gln265Ala/Thr436Ala/Asn438Ala attracted our attention by showing a 5‐ to 18‐fold improvement in hydration of **1 f**, **1 g**, and **1 i** (Figure S53, S54, and S56). Most likely, this can be attributed to the larger and more hydrophobic opening of the substrate binding channel, which is lined out by less ester substrate‐compatible amino acids in the wild‐type enzyme (Figure 1 e). Based on control experiments, we rule out that the hydration of OA esters in *E. coli* cells occurred through sequential ester hydrolysis,^46^ oleic acid hydration and esterification of (*R*)‐10‐hydroxy stearic acid. Co‐incubation of **1 f** and **1 g** with methanol, ethanol or *i*‐propanol in bioconversions did not result in the formation of any ester side product derived from a *trans*‐ or re‐esterification reaction (Figure S58 and S59). It is especially noteworthy that selected OhyA double variants and the triple alanine variant allowed the conversion of sterically demanding fatty acid ester derivatives **1 h** and **1 j** (Figure 1 f), whereas the wild‐type enzyme was inactive on these structures (see Figure S8, S10, S55, and S57 for GC‐MS and GC‐FID data, and Figure S23, S24, S27, and S28 for NMR spectra). We also performed activity assays with a respective quadruple alanine variant (OhyA Gln265Ala/Thr436Ala/Asn438Ala/His442Ala), but did not end up with conversion of any of the OA esters. This suggested that the exchange of histidine at position 442 for an alanine was severely affecting enzyme activity.

In summary, we describe the first regio‐ and stereoselective addition of water to fatty acid derivatives without the prerequisite of a free carboxylate. Stabilizing the interaction between the head groups of OA derivatives and the substrate binding site of OhyA by rational design markedly enhanced the activity on eight different OA derivatives, including amide, hydroxamic acid, alcohol and esters, while the highly *R*‐selective hydration of the *cis*‐9 double bond (*ee* ≥95 %) was retained in all cases (Table 2).

**Table 2:** List of individually best OhyA‐variants for the regio‐ and stereoselective hydration of OA (**1 a**) and OA derivatives (**1 c**–**1 j**) obtained by site‐directed mutagenesis of substrate binding residues.

Compound	Best variant for hydration reaction	Improvement compared to wild type^[a]^	Yield [%]^[b]^	Abs. conf. at C‐10^[c]^
a	Wild type	–	93	*R*
b	No conversion	–	0	*–*
c	Gln265Ser/ Asn438Asp	2.7	54	*R*
d	Gln265Ala/Thr436Ala/Asn438Ala	3.6	8	*R*
e	Gln265Ala/Thr436Ala/Asn438Ala	2.0	30	*R*
f	Gln265Ala/Thr436Ala/Asn438Ala	5.2	6	*R*
g	Gln265Ala/Thr436Ala/Asn438Ala	8.8	12	*R*
h	Gln265Ala/Thr436Ala/Asn438Ala	Wild type inactive	1	*R*
i	Gln265Ala/Thr436Ala/Asn438Ala	17.6	4	*R*
j	Gln265Ala/Thr436Ala/Asn438Ala	Wild type inactive	2	*R*

[a] Ratio of conversions obtained after 96 h compared to the wild‐type enzyme in application; quantified by GC‐FID analysis. [b] Isolated yield after chromatography. [c] *ee* values ≥95 % (*R*) in all cases as determined by ^1^H NMR spectroscopy after esterification of the 10‐hydroxy group with (*S*)‐(+)‐*O*‐acetylmandelic acid.^38^

Our work allows for a deeper understanding of the substrate recognition in this enzyme class and, to our knowledge, reports the first successful engineering of a FAHY towards improved conversion of OA derivatives. This knowledge provides a foundation for the development of FAHYs towards applications beyond current limitations.

## Conflict of interest

The authors declare no conflict of interest.

## Supporting information

As a service to our authors and readers, this journal provides supporting information supplied by the authors. Such materials are peer reviewed and may be re‐organized for online delivery, but are not copy‐edited or typeset. Technical support issues arising from supporting information (other than missing files) should be addressed to the authors.

Supplementary
