# Supplementary material for: Evolving the Promiscuity of Elizabethkingia meningoseptica Oleate Hydratase for the Regio‐ and Stereoselective Hydration of Oleic Acid Derivatives
Source: Angew Chem Int Ed Engl. 2019 Apr 17;58(22):7480–4. doi: 10.1002/anie.201901462 (PMC6563698; doi:10.1002/anie.201901462)
Supplement: Supplementary file 1 — Supplementary [file ANIE-58-7480-s001.pdf]

Angewandte Chemie
Eine Zeitschrift der Gesellschaft Deutscher Chemiker

Supporting Information

Evolving the Promiscuity of *Elizabethkingia meningoseptica* Oleate Hydratase for the Regio- and Stereoselective Hydration of Oleic Acid Derivatives

*Matthias Engleder[+], Gernot A. Strohmeier[+], Hansjörg Weber, Georg Steinkellner, Erich Leitner, Monika Müller, Daniel Mink, Martin Schürmann, Karl Gruber, and Harald Pichler**

anie_201901462_sm_miscellaneous_information.pdf

Supporting Information
Table of Contents

Table of Contents....1
Experimental Procedures....2
General....2
Plasmid and expression strain construction....2
Amino acid sequence alignments....2
Site-directed mutagenesis....2
Recombinant protein expression....4
In vitro conversion of OA and OA derivatives with cell-free lysate....4
Whole cell bioconversions....4
GC-MS analyses....4
GC-FID analyses....5
Preparation of OA derivatives....5
Preparative-scale hydration of OA derivatives....5
Purification of crude reaction products....5
NMR analysis of reaction products....5
Modeling of OA and derivatives to the OhyA 3D structure....5
Results and Discussion....6
Nucleotide information....6
GC-MS monitoring of the hydration of OA derivatives....6
Spectroscopic and optical data of purified reaction products....15
NMR spectra of reaction products....17
Amino acid sequence alignment....26
Conversion of OA derivatives with OhyA wild type and substrate binding variants....28
Stereoscopic imaging of modeling studies....36
Determination of the enantiomeric excess of reaction products by ^{1}H-NMR analysis....38
Determination of OhyA wild type and variant conversions by GC-FID....41
Control reactions with OA esters....45
References....47
Author contributions....48

Experimental Procedures

General

Unless stated otherwise, standard laboratory reagents were obtained from Sigma-Aldrich® (Steinheim, Germany) or Carl Roth GmbH & Co. KG (Karlsruhe, Germany) with the highest purity available. Oleic acid (OA) and esters thereof (methyl, ethyl, *i*-propyl), oleyl alcohol and oleyl amine were purchased from Sigma-Aldrich® (Steinheim, Germany). The OA methyl and ethyl ester and oleyl alcohol were distilled prior to use to a purity >90% according to GC-FID analysis. (*R*)-10-hydroxy stearic acid was obtained from DSM Innovative Synthesis B.V. (Geleen, Netherlands). Other starting materials used for the investigations were synthesized from suitable precursors as described below or used as received from Sigma-Aldrich®. Flash column chromatography was performed on Acros Organics silica gel 0.035-0.070 mm, 60 Å. Analytical thin layer chromatography (TLC) was performed using TLC-plates from Merck (TLC aluminium foil, silica gel 60 F254) and subsequent visualization with cerium ammonium molybdate stain. The specific optical rotations were determined on a Perkin Elmer Polarimeter 341 with an integrated sodium vapor lamp. All samples were measured at the D-line of the sodium light (λ = 589 nm).

Plasmid and expression strain construction

Restriction enzymes were acquired from Thermo Scientific (St. Leon-Rot, Germany). Sterile water was purchased from Fresenius Kabi (Graz, Austria*).* Molecular cloning of the expression vector was performed according to standard procedures[1] and correct integration of the insert was confirmed by sequencing (LGC Genomics, Berlin, Germany). For gene amplification, Phusion® High Fidelity DNA polymerase (Thermo Fisher Scientific Inc., St. Leon-Rot, Germany) was utilized in accordance with the recommended PCR protocol. A codon-optimized gene variant of OhyA (*Elizabethkingia meningoseptica* XP_001209325 oleate hydratase) was purchased from DNA2.0 (Menlo Park, CA). For expression of recombinant OhyA, a modified pMS470 expression vector, pMS470-HISTEV-OhyA was constructed as described previously.[2] For all cloning steps and plasmid replication, *E. coli* Top10 F’ (F'[*lacI*^{q} Tn*10*(tet^{R})] *mcrA* Δ(*mrr-hsd*RMS-*mcrBC*) φ80*lacZ*ΔM15 Δ*lacX*74 *deoR nupG recA1 araD139* Δ(*ara-leu*)7697 *galU galK rpsL*(Str^{R}) *endA1* λ^{-}) from Life technologies (Vienna, Austria) was used. Recombinant OhyA was expressed in *E. coli* BL21Star™ (DE3) (F- *ompT hsdS*$_{B}$ ($r_B^- m_B^-$) *gal dcm rne131* (DE3)) (Life technologies, Vienna, Austria).

Amino acid sequence alignments

The protein sequence of OhyA was compared to the amino acid sequences in the Hydratase Engineering Database (HyED), in which a total of 2046 sequences are collected.[3] Since OhyA is categorized in homologous family 11 (HFam11) of the HyED, all amino acid sequences from HFam11 were selected for the multiple sequence alignment. Sequences were extracted from the database for a multiple sequence alignment with the Clustal Omega sequence alignment tool using default settings,[4] and were visualized with the Unipro UGene software.

Site-directed mutagenesis

Amino acid exchange variants of OhyA were generated by site-directed mutagenesis using a modified Stratagene QuikChange™ site-directed mutagenesis protocol. Twenty-five µL of two separate PCR reactions containing forward and reverse primers, respectively, were prepared (**Fehler! Verweisquelle konnte nicht gefunden werden.**Table S1). After five cycling steps, PCR reactions were combined and the PCR was continued for 20 additional cycles. Mutated plasmids were verified by sequencing of the coding regions of the constructs.

Table S1. Primers used for introduction of point mutations into the OhyA nucleotide sequence. The underlined bases mark the mutated codons.

Primer name	Primer sequence (from 5’ to 3’)
Fw(OhyA_Gln265Ala)	GTTTCCGAAGTACAATGCATATGACACGTTTGTC
Rv(OhyA_Gln265Ala)	GACAAACGTGTCATATGCATTGTACTTCGGAAAC
Fw(OhyA_Gln265Glu)	GTTTCCGAAGTACAATGAATATGACACGTTTGTC
Rv(OhyA_Gln265Glu)	GACAAACGTGTCATATTCATTGTACTTCGGAAAC
Fw(OhyA_Gln265Lys)	GTTTCCGAAGTACAATAAATATGACACGTTTGTC

Rv(OhyA_Gln265Lys)	GACAAACGTGTCATATTTATTGTACTTCGGAAAC
Fw(OhyA_Gln265Ser)	GTTTCCGAAGTACAATTCTTATGACACGTTTGTC
Rv(OhyA_Gln265Ser)	GACAAACGTGTCATAAGAATTGTACTTCGGAAAC
Fw(OhyA_Thr436Ala)	TGGTTGATGAGCTTTGCGTGCAATCGCCAGCCG
Rv(OhyA_Thr436Ala)	CGGCTGGCGATTGCACGCAAAGCTCATCAACCA
Fw(OhyA_ Thr436Asn)	TGGTTGATGAGCTTTAACTGCAATCGCCAGCCG
Rv(OhyA_ Thr436Asn)	CGGCTGGCGATTGCAGTTAAAGCTCATCAACCA
Fw(OhyA_ Thr436Asp)	TGGTTGATGAGCTTTGATTGCAATCGCCAGCCG
Rv(OhyA_ Thr436Asp)	CGGCTGGCGATTGCAATCAAAGCTCATCAACCA
Fw(OhyA_ Thr436Lys)	TGGTTGATGAGCTTTAAATGCAATCGCCAGCCG
Rv(OhyA_ Thr436Lys)	CGGCTGGCGATTGCATTTAAAGCTCATCAACCA
Fw(OhyA_Asn438Ala)	GATGAGCTTTACCTGCGCACGCCAGCCGCATTTCC
Rv(OhyA_Asn438Ala)	GGAAATGCGGCTGGCGTGCGCAGGTAAAGCTCATC
Fw(OhyA_Asn438Arg)	GATGAGCTTTACCTGCCGCCGCCAGCCGCATTTCC
Rv(OhyA_Asn438Arg)	GGAAATGCGGCTGGCGGCGGCAGGTAAAGCTCATC
Fw(OhyA_Asn438Asp)	GATGAGCTTTACCTGCGCACGCCAGCCGCATTTCC
Rv(OhyA_Asn438Asp)	GGAAATGCGGCTGGCGTGCGCAGGTAAAGCTCATC
Fw(OhyA_Asn438Lys)	GATGAGCTTTACCTGCAAACGCCAGCCGCATTTCC
Rv(OhyA_Asn438Lys)	GGAAATGCGGCTGGCGTTTGCAGGTAAAGCTCATC
Fw(OhyA_Asn438Ser)	GATGAGCTTTACCTGCAGCCGCCAGCCGCATTTCC
Rv(OhyA_Asn438Ser)	GGAAATGCGGCTGGCGGCTGCAGGTAAAGCTCATC
Fw(OhyA_His442Ala)	CTGCAATCGCCAGCCGGCCTTCCCGGAGCAGCCGG
Rv(OhyA_His442Ala)	CCGGCTGCTCCGGGAAGGCCGGCTGGCGATTGCAG
Fw(OhyA_His442Asn)	CTGCAATCGCCAGCCGAATTTCCCGGAGCAGCCGG
Rv(OhyA_His442Asn)	CCGGCTGCTCCGGGAAATTCGGCTGGCGATTGCAG
Fw(OhyA_His442Asp)	CTGCAATCGCCAGCCGGATTTCCCGGAGCAGCCGG
Rv(OhyA_His442Asp)	CCGGCTGCTCCGGGAAATCCGGCTGGCGATTGCAG
Fw(OhyA_His442Gln)	CTGCAATCGCCAGCCGCAATTCCCGGAGCAGCCGG
Rv(OhyA_His442Gln)	CCGGCTGCTCCGGGAATTGCGGCTGGCGATTGCAG
Fw(OhyA_His442Glu)	CTGCAATCGCCAGCCGGAATTCCCGGAGCAGCCGG
Rv(OhyA_His442Glu)	CCGGCTGCTCCGGGAATTCCGGCTGGCGATTGCAG
Fw(OhyA_His442Tyr)	CTGCAATCGCCAGCCGTATTTCCCGGAGCAGCCGG
Rv(OhyA_His442Tyr)	CCGGCTGCTCCGGGAAATACGGCTGGCGATTGCAG
Fw(OhyA_ Asn438Ala/His442Ala)	GCGCACGCCAGCCGGCTTTCCCGGAGCAGCCGGAT
Rv(OhyA_ Asn438Ala/His442Ala)	ATCCGGCTGCTCCGGGAAAGCCGGCTGGCGTGCGC
Fw(OhyA_Thr436Asp/Asn438Asp)	GGTTGATGAGCTTTGACTGCGACCGCCAGCCGCATT
Rv(OhyA_Thr436Asp/Asn438Asp)	AATGCGGCTGGCGGTCGCAGTCAAAGCTCATCAACC
Fw(OhyA_Thr436Asp/Asn438Ser)	GGTTGATGAGCTTTGACTGCAGCCGCCAGCCGCATT
Rv(OhyA_Thr436Asp/Asn438Ser)	AATGCGGCTGGCGGCTGCAGTCAAAGCTCATCAACC

Fw(OhyA_Asn438Ala/His442Ala)	GCGCACGCCAGCCGGCTTTCCCGGAGCAGCCGGAT
Rv(OhyA_Asn438Ala/His442Ala)	ATCCGGCTGCTCCGGGAAAGCCGGCTGGCGTGCGC

Recombinant protein expression

OhyA was recombinantly expressed in E. coli. First, a pre-culture was inoculated with E. coli BL21 Star (DE3) cells harboring pMS470-HISTEV-OhyA wild type enzyme or variants, and grown in LB supplemented with 100 µg mL-1 ampicillin at 28°C and 130 rpm overnight. Main cultures were inoculated to an OD600 of 0.1 in auto induction medium (AIM) - Terrific Broth Base including Trace elements (Formedium, UK) containing 100 µg mL-1 ampicillin. Recombinant protein was expressed at 28°C and 130 rpm for 22 h. Cells were harvested by centrifugation for 10 min at 4,400 x g and 22°C, and were instantly used for whole cell biotransformations or frozen at -20°C until preparation of cell-free lysates.

In vitro conversion of OA and OA derivatives with cell-free lysate

For preparation of cell-free lysates containing recombinant hydratase enzyme, thawed cell pellets were resuspended in 50 mM HEPES, pH 7.4. Cells were lysed by ultrasonication for 4 min with a Sonifier® 250 (Branson, Danbury, CT) setting the duty cycle to 80% and the output control to level 8. Cell-free lysate was separated from the total lysate by centrifugation for 35 min at 48,300 x g and 4°C. In vitro activity assays were performed with 2 mg of *E. coli* cell-free lysate in Pyrex® glass culture tubes (Corning, NY). Cell-free lysate was incubated with 2 mM substrates **1a**–**1j** in 1 mL of 50 mM HEPES, pH 6.0, and 2% (v/v) of ethanol. Assays were shaken over night at 25°C and 150 rpm in the presence of 1 mM *n*-pentadecanoic acid as internal standard. After conversion, assays were quenched either by acidification to pH 2.0 with 0.12 M HCl and extraction with 2 × 2 mL of ethyl acetate (in case of **1a** and **1c**–**1j**), or only by extraction with 2 × 2 mL of ethyl acetate (in case of **1b**) while agitating on a Vibrax VXR basic shaker (IKA, Germany) for 30 min. The suspension was centrifuged for 5 min at 2,900 × g and 22°C to improve separation of the phases. Combined organic phases were concentrated under a N_2 stream. Fatty acid derivatives were silylated with 10 µL of pyridine and 50 µL of *N*,*O*-bis(trimethylsilyl)trifluoroacetamide (BSTFA). After incubation for 30 min at 500 rpm, the reaction mixtures were diluted with 200 µL of ethyl acetate and analyzed by GC-MS or GC-FID.

Whole cell bioconversions

Bioconversion assays of **1a**–**j** were performed with *E. coli* BL21 Star (DE3) cells immediately after expression of recombinant OhyA. Fifty OD_{600} units, which are corresponding to a cell dry weight of 50 mg, were resuspended in 50 mM HEPES, pH 6.0, supplemented with 100 mM glucose and 0.2 mM FAD in Pyrex® glass culture tubes (Corning, NY). Biotransformations at 1 mL scale were started by adding substrate to a final concentration of 2 mM from an ethanolic stock solution (100 mM). *n*-pentadecanoic acid (1 mM) was used as internal standard. The reactions were conducted in the presence of 2% (v/v) of ethanol as co-solvent at 30°C and shaking at 150 rpm at a defined angle of the Pyrex® tubes (55°). Biotransformations were performed for 22 h or 96 h.

Whole cell bioconversions of OA esters **1f** and **1g** with alcohol additives were performed with 50 mg of *E. coli* BL21 Star (DE3) after recombinant expression of OhyA Gln265Ala/Thr436Ala/Asn438Ala. Biotransformations of **1f** were co-incubated with equimolar concentrations of the substrate and either ethanol or *i*-propanol, and biotransformations of **1g** were co-incubated with equimolar concentrations of the substrate and either methanol or *i*-propanol. Otherwise, assay conditions were maintained as described above.

GC-MS analyses

Free fatty acids and derivatives thereof were initially analyzed and identified by gas chromatography-mass spectrometry (GC-MS). A HP-5 column (crosslinked 5% Ph-Me Polysiloxane; 30 m length, 0.25 mm in diameter and 0.25 µm film thickness) on a Hewlett-Packard 6890 Series II GC equipped with a mass selective detector was used. Sample aliquots of 1 µL were injected in split mode (split ratio 30:1) at 240°C injector temperature and 290°C detector temperature with N_2 as carrier at a flow rate set to 36 cm s^{-1} in constant flow mode. The temperature program was as follows: 100°C for 1 min, 15°C min^{-1} to 300°C, hold for 5 min. The total run time was 19.33 min. The mass selective detector was operated in a mass range of 50-400 amu at an electron multiplier voltage of 1765 V. Results were evaluated with the GC-MS Data Analysis software (Agilent Technologies, Austria).

GC-FID analyses

Product formation was quantified by GC after derivatization of extracted samples with BSTFA. A Shimadzu GC-2010 Plus instrument equipped with a flame ionization detector and a Phenomenex Zebron ZB-5 column (crosslinked 5% Ph-Me Polysiloxane; 30 m length, 0.32 mm in diameter and 0.25 µm film thickness) was used. Sample aliquots of 1 µL were injected in split mode (split ratio 10:1) at 240°C injector temperature and 320°C detector temperature. N_2 was used as carrier gas at a flow rate set to 20 cm s^{-1} in constant flow mode. The oven temperature program was as follows: 70°C for 4 min, 35°C min^{-1} to 300°C, hold for 5 min. The total run time was 15.57 min.

Preparation of OA derivatives

Oleamide (**1c**) was obtained via a literature procedure[5] and the material was purified through recrystallization from acetone[6] to a purity of 95% as checked by rp-HPLC at 210 nm. *n*-propyl (**1i**) and *n*-butyl oleate (**1j**) were synthesized from OA (**1a**) via Fischer esterification as described in the literature[7] and purified via flash chromatography on silica gel using cyclohexane/ethyl acetate 20:1 as eluent.
n-propyl oleate (**1i**):
^{1}H-NMR (300 MHz, $CDCl_3$): δ = 0.88 (3H, t, ^3J(H,H) = 6.7 Hz, Me), 0.94 (3H, t, ^3J(H,H) = 7.4 Hz, -$CO_2CH_2CH_2CH_2\underline{Me}$), 1.15-1.45 (20H, m, 10 CH_2), 1.52-1.70 (4H, m, 2 CH_2), 1.92-2.10 (4H, m, 2 CH_2), 2.29 (2H, t, ^3J(H,H) = 7.5 Hz, -$C\underline{H}_2$-$CO_2CH_2CH_2Me$), 4.02 (2H, t, ^3J(H,H) = 6.7 Hz, -$CO_2C\underline{H}_2CH_2Me$), 5.34 (2H, m, -C$\underline{H}$=C$\underline{H}$-).
^{13}C-NMR (75 MHz, $CDCl_3$): δ = 10.54, 14.25, 22.17, 22.83, 25.17, 27.32, 27.37, 29.26, 29.29, 29.32, 29.47 (2×C), 29.67, 29.84, 29.92, 32.06, 34.54, 65.97, 129.91, 130.15, 174.13.
n-butyl oleate (**1j**):
^{1}H-NMR (300 MHz, $CDCl_3$): δ = 0.88 (3H, t, ^3J(H,H) = 6.7 Hz, Me), 0.93 (3H, t, ^3J(H,H) = 7.4 Hz, -$CO_2CH_2CH_2CH_2\underline{Me}$), 1.15-1.45 (22H, m, 11 CH_2), 1.52-1.70 (4H, m, 2 CH_2), 1.92-2.10 (4H, m, 2 CH_2), 2.29 (2H, t, ^3J(H,H) = 7.5 Hz, -$C\underline{H}_2$-$CO_2CH_2CH_2CH_2Me$), 4.07 (2H, t, ^3J(H,H) = 6.6 Hz, -$CO_2C\underline{H}_2CH_2CH_2Me$), 5.34 (2H, m, -C$\underline{H}$=C$\underline{H}$-).
^{13}C-NMR (75 MHz, $CDCl_3$): δ = 13.85, 14.25, 19.30, 22.83, 25.17, 27.32, 27.37, 29.26, 29.29, 29.31, 29.47 (2×C), 29.67, 29.84, 29.92, 30.87, 32.06, 34.55, 64.25, 129.91, 130.15, 174.13.

Preparative-scale hydration of OA derivatives

OA derivatives **1c**–**1j** were hydrated to **2c**–**2j** in a semi-preparative scale. Twenty to 150 mg of non-physiological substrates were converted in 1 mL scale whole cell bioconversions. Each reaction contained 200 mg of *E. coli* cells in Pyrex® glass tubes, after over-expression of OhyA Gln265Ala/Thr436Ala/Asn438Ala, resuspended in 50 mM HEPES, pH 6.0, containing 100 mM glucose and 0.2 mM FAD. Biotransformations were incubated for 96 h at 30°C and 150 rpm at a defined angle of the Pyrex® tubes (55°). After quenching by acidification to pH 2.0 with 0.12 M HCl, the suspensions were extracted with ethyl acetate (3 × 2 mL for 30 min) with intermittent centrifugation for 5 min at 2,900 × g and 22°C to improve phase separation. The organic phases were quantitatively collected and concentrated under a stream of N_2.

Purification of crude reaction products

The products extracted with ethyl acetate were purified via flash chromatography (9.5 g silica gel, 20 × 1 cm column size) using eluent mixtures of cyclohexane/ethyl acetate in ratios from 10:1 to 1:1 (v/v) dependent on the polarity of the respective derivative. All fractions containing the desired product were pooled and evaporated to dryness.

NMR analysis of reaction products

^{1}H and ^{13}C NMR spectra were recorded on a Bruker AVANCE III 300 spectrometer (^{1}H: 300.36 MHz; ^{13}C: 75.53 MHz) or a Varian INOVA 500 (^{1}H: 499.88 MHz; ^{13}C: 125.71 MHz). Chemical shifts were referenced to residual protonated solvent signals as internal standard. Chemical shift values are reported in parts per million, and coupling constants (*J* values) are given in Hertz. Abbreviations for ^{1}H-NMR signals are as follows: s, singlet; d, doublet; t, triplet; dd, doublet of doublets; and m, multiplet.

Modeling of OA and derivatives to the OhyA 3D structure

Docking of OA to the OhyA 3D structure was performed using AutoDock implemented in YASARA structure as described previously.[2] Briefly, receptor (chain A of OhyA; PDB code: 4uir) and ligand (OA; formal charge of -1) were prepared and energy minimized with the Schrodinger package. The receptor was kept rigid, and the ligand had full

conformational flexibility around each single bond. The docking box (x=25 Å, y=27 Å, z= 25 Å) was set to be close to the flavin cofactor covering the elongated active site cavity. After 50 individual runs, the best docking modes were sorted by binding energies and chemical plausibility and were finally clustered according to a maximum root-mean-square deviation (r.m.s.d.) of the heavy atoms of 2.0 Å.

OA derivatives **1c**–**1j** were prepared using YASARA and AM1 charges were applied accordingly. The derivatives **1c**–**1j** were docked to the 3D structure model of OhyA after introducing amino acid exchanges that resulted in the best activity on each substrate. The binding mode with the lowest docking energy was visually inspected. Amino acid exchanges were introduced in silico using YASARA. Docking was performed using VINA[8] implemented in YASARA structure in analogy to OA after substitution of the carboxylate for the different head groups of **1c**–**1j** using 20 independent docking runs and a 2.0 Å r.m.s.d cluster deviation.

The docking with the lowest energy for **1i** and **1j** did not result in a productive binding mode, even in the triple variant (visual inspection). This may be due to the rigid receptor docking, which resulted in higher docking energies for the larger derivatives in the productive binding mode. By overlaying of **1i** and **1j** with the docked OA and by performing an energy minimization step (Force Field: Amber03), the ligands **1i** and **1j** fitted quite well into the larger pocket introduced by the mutations. It can be assumed that small changes in the structure on the site of the mutations, although not optimal, do also provide enough space for the larger derivatives **1i** and **1j**.

Results and Discussion

Nucleotide information

Open reading frame of the codon-optimized *E. meningoseptica* oleate hydratase gene (OhyA, GenBank: ACT54545.1) used in this study:

5’ - ATGCATCACCATCACCATCACCATCACCATCACAACCCAATCACCAGCAAATTCGACAAAGTCCTGAACGCATCC
AGCGAGTACGGCCACGTTAATCACGAACCGGATAGCAGCAAAGAGCAGCAACGCAACACCCCGCAGAAGTCCATG
CCATTTAGCGATCAAATCGGCAACTATCAACGTAACAAAGGTATTCCGGTTCAGAGCTATGATAATTCGAAGATTTAC
ATCATTGGTTCTGGTATTGCGGGTATGTCGGCTGCGTACTACTTCATCCGTGACGGTCACGTTCCGGCGAAGAACA
TCACGTTCCTGGAGCAACTGCACATTGATGGCGGCTCTCTGGATGGTGCTGGCAACCCGACCGACGGCTATATCAT
CCGTGGTGGTCGTGAAATGGATATGACCTACGAGAACCTGTGGGATATGTTCCAGGATATTCCGGCGCTGGAGATG
CCGGCACCGTATAGCGTTCTGGATGAATATCGTCTGATTAATGACAACGATAGCAATTACAGCAAAGCACGTCTGAT
CAACAATAAGGGCGAAATCAAGGACTTCAGCAAGTTTGGTCTGAATAAGATGGACCAGCTGGCCATCATCCGTCTG
TTGCTGAAAAACAAAGAAGAGCTGGACGACTTGACCATTGAGGACTACTTCTCTGAGAGCTTTCTGAAAAGCAATTT
CTGGACGTTTTGGCGCACGATGTTCGCGTTTGAGAACTGGCATAGCCTGTTGGAACTGAAGCTGTACATGCACCGC
TTCCTGCACGCCATTGACGGTCTGAACGACCTGAGCAGCCTGGTGTTTCCGAAGTACAATCAATATGACACGTTTGT
CACGCCGCTGCGTAAATTCCTGCAAGAAAAGGGTGTTAACATCCACTTGAATACCTTGGTCAAGGATCTGGATATTC
ACATCAATACCGAGGGTAAAGTCGTCGAGGGCATCATTACCGAGCAAGACGGTAAAGAGGTAAAGATTCCGGTGG
GTAAGAATGACTATGTTATCGTGACGACCGGTTCCATGACCGAGGACACGTTTTACGGTAACAACAAAACCGCACC
GATCATTGGTATCGACAATAGCACTAGCGGTCAGAGCGCTGGCTGGAAACTGTGGAAGAACCTGGCTGCCAAGAG
CGAAATCTTCGGCAAGCCGGAGAAATTCTGTAGCAATATTGAGAAATCCGCGTGGGAAAGCGCGACCCTGACGTGT
AAACCTTCCGCGTTGATCGACAAACTGAAAGAATATTCGGTCAACGACCCGTACAGCGGTAAGACCGTGACCGGCG
GTATCATTACTATCACCGATAGCAACTGGTTGATGAGCTTTACCTGCAATCGCCAGCCGCATTTCCCGGAGCAGCC
GGATGACGTCCTGGTGCTGTGGGTGTATGCGCTGTTTATGGATAAAGAAGGTAACTACATTAAGAAAACCATGCTG
GAGTGCACCGGTGATGAGATTTTGGCGGAGCTGTGTTACCATCTGGGCATTGAAGATCAGCTGGAAAATGTGCAGA
AGAATACGATTGTTCGCACCGCATTCATGCCGTATATCACGAGCATGTTTATGCCACGTGCCAAAGGTGACCGCCC
TCGTGTGGTCCCGGAAGGTTGCAAAAACCTGGGCCTGGTTGGTCAATTTGTGGAAACGAACAATGACGTCGTGTTT
ACGATGGAATCTAGCGTTCGCACGGCCCGTATTGCGGTGTACAAGTTGCTGAATCTGAACAAGCAGGTGCCGGACA
TTAATCCGCTGCAATACGATATTCGCCATCTGCTGAAAGCGGCAAAGACCCTGAATGATGACAAACCGTTCGTGGG
CGAAGGCTTGCTGCGTAAGGTTCTGAAAGGCACCTATTTTGAGCACGTTCTGCCTGCGGGTGCAGCGGAAGAAGA
AGAGCATGAGAGCTTCATTGCGGAACATGTTAACAAGTTCCGTGAGTGGGTCAAGGGTATCCGTGGCTAATAA - 3’

GC-MS monitoring of the hydration of OA derivatives

Bioconversions of **1a**–**1j** were performed with whole *E. coli* cells. Representative GC-chromatograms of technical triplicates of an authentic substrate standard, an OhyA-free *E. coli* strain (empty vector control, EVC) and a biotransformation with cells after over-expression of OhyA are overlaid (Figure S1–Figure **S10**). In case a substrate was not converted with the wild type enzyme (**1h** and **1j**), a representative chromatogram of the OhyA Gln265Ala/Thr436Ala/Asn438Ala bioconversion is shown. The OA-derived *N*-hydroxy oleamide (**1d**) and the hydrated reaction product (**2d**) were both detected as the respective isocyanates after a Lossen rearrangement occurring under

GC-MS analysis conditions.[9] Moreover, conversion of **1d** with the *E. coli* EVC and the strain expressing OhyA led to the unexpected formation of oleamide (**1c**), with a subsequent hydration to 10-hydroxy octadecanamide (**2c**) only in OhyA biotransformations. Since *N*-hydroxy oleamide (**1d**) was initially oleamide-free, one must assume that the oleamide (**1c**) was formed by degradation of *N*-hydroxy oleamide (**1d**) in *E. coli*.[10]

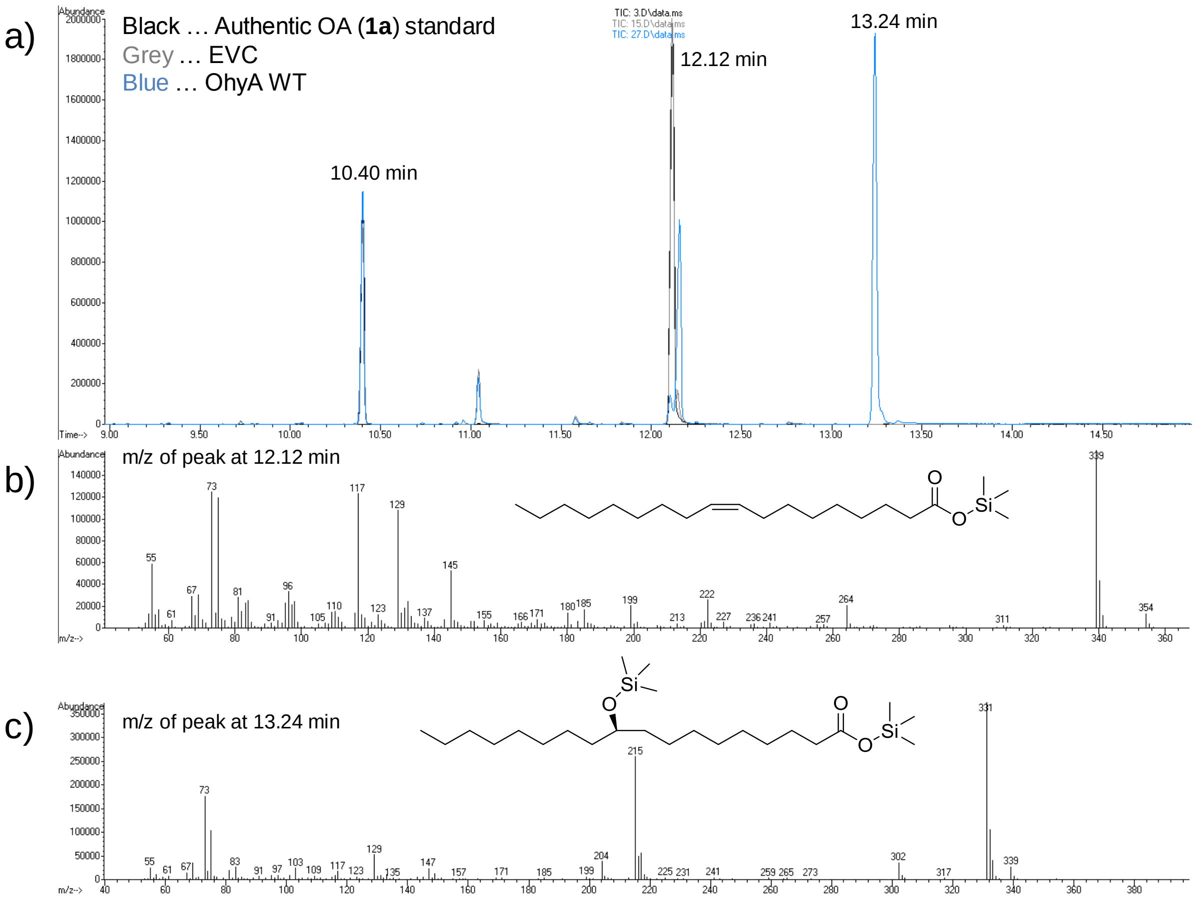

Figure S1. Bioconversion of OA (**1a**) to 10-hydroxy stearic acid (**2a**) with *E. coli* whole cells over-expressing OhyA wild type enzyme. a) Overlay of representative GC-MS chromatograms from technical triplicates of an authentic **1a** standard and biotransformations of **1a** with an *E. coli* empty vector control (EVC) and an *E. coli* strain over-expressing OhyA wild type. Retention times of the TMS-derivatives of the internal standard *n*-pentadecanoic acid (10.40 min), **1a** (12.12 min) and **2a** (13.24 min) are highlighted. b) Mass spectrum of the peak at 12.12 min, corresponding to the TMS-derivative of **1a**. c) Mass spectrum of the peak at 13.24 min, corresponding to the TMS-derivative of **2a**.

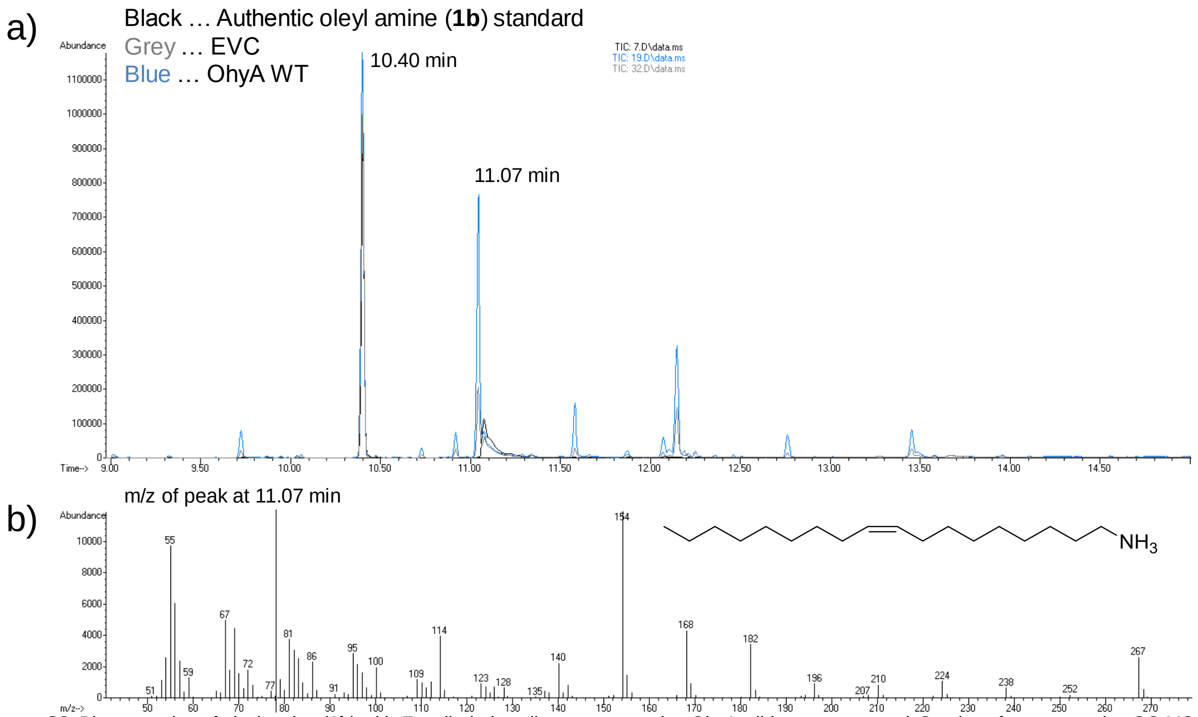

Figure S2. Bioconversion of oleyl amine (**1b**) with *E. coli* whole cells over-expressing OhyA wild type enzyme. a) Overlay of representative GC-MS chromatograms from technical triplicates of an authentic **1b** standard and biotransformations of **1b** with an *E. coli* empty vector control (EVC) and an *E. coli* strain over-expressing OhyA wild type. Retention times of the TMS-derivatives of the internal standard *n*-pentadecanoic acid (10.40 min) and **1b** (11.07 min) are highlighted. b) Mass spectrum of the peak at 11.07 min, corresponding to the TMS-derivative of **1b**.

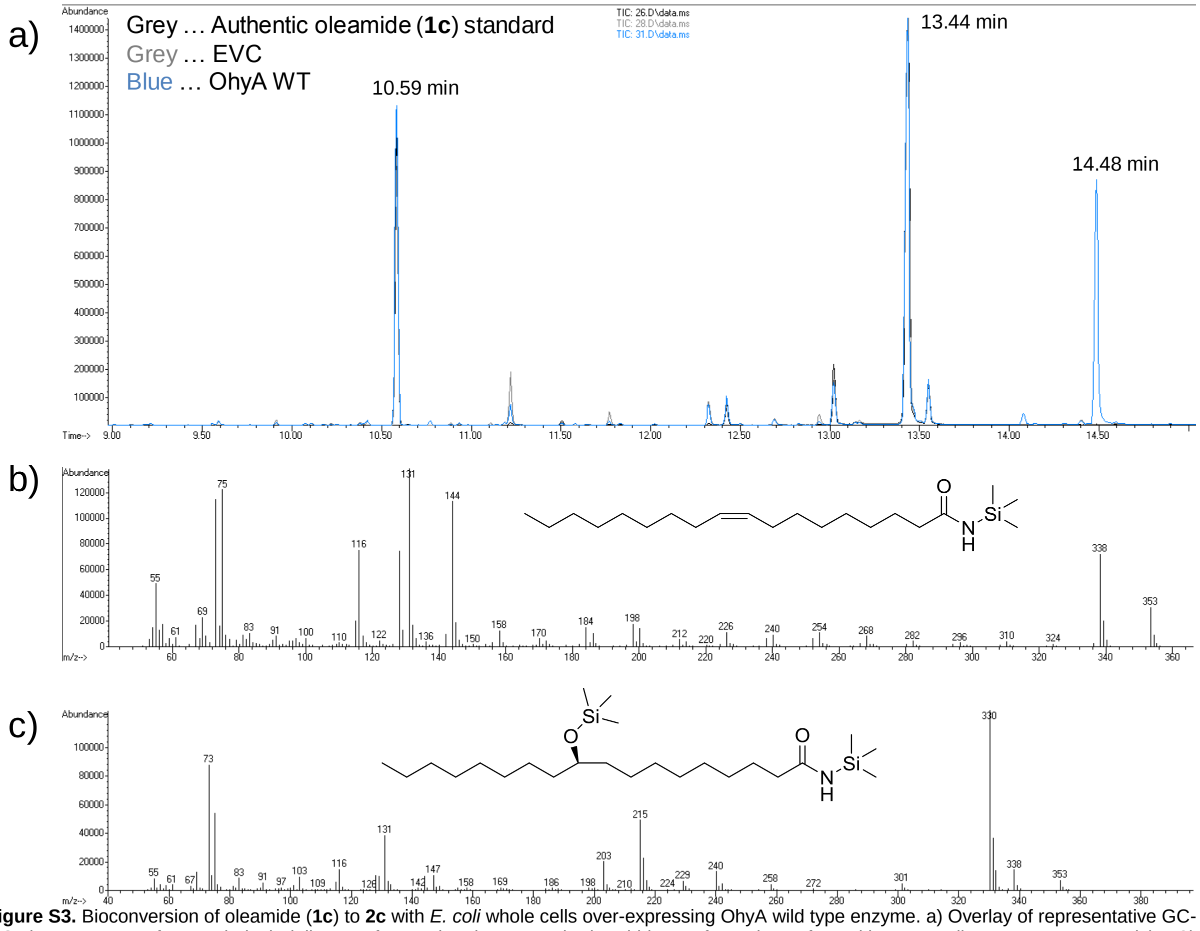

Figure S3. Bioconversion of oleamide (**1c**) to **2c** with *E. coli* whole cells over-expressing OhyA wild type enzyme. a) Overlay of representative GC-MS chromatograms from technical triplicates of an authentic **1c** standard and biotransformations of **1c** with an *E. coli* empty vector control (EVC) and an *E. coli* strain over-expressing OhyA wild type. Retention times of the TMS-derivatives of the internal standard *n*-pentadecanoic acid (10.59 min), **1c** (13.44 min) and **2c** (14.48 min) are highlighted. b) Mass spectrum of the peak at 13.44 min, corresponding to the TMS-derivative of **1c**. c) Mass spectrum of the peak at 14.48 min, corresponding to the TMS-derivative of **2c**.

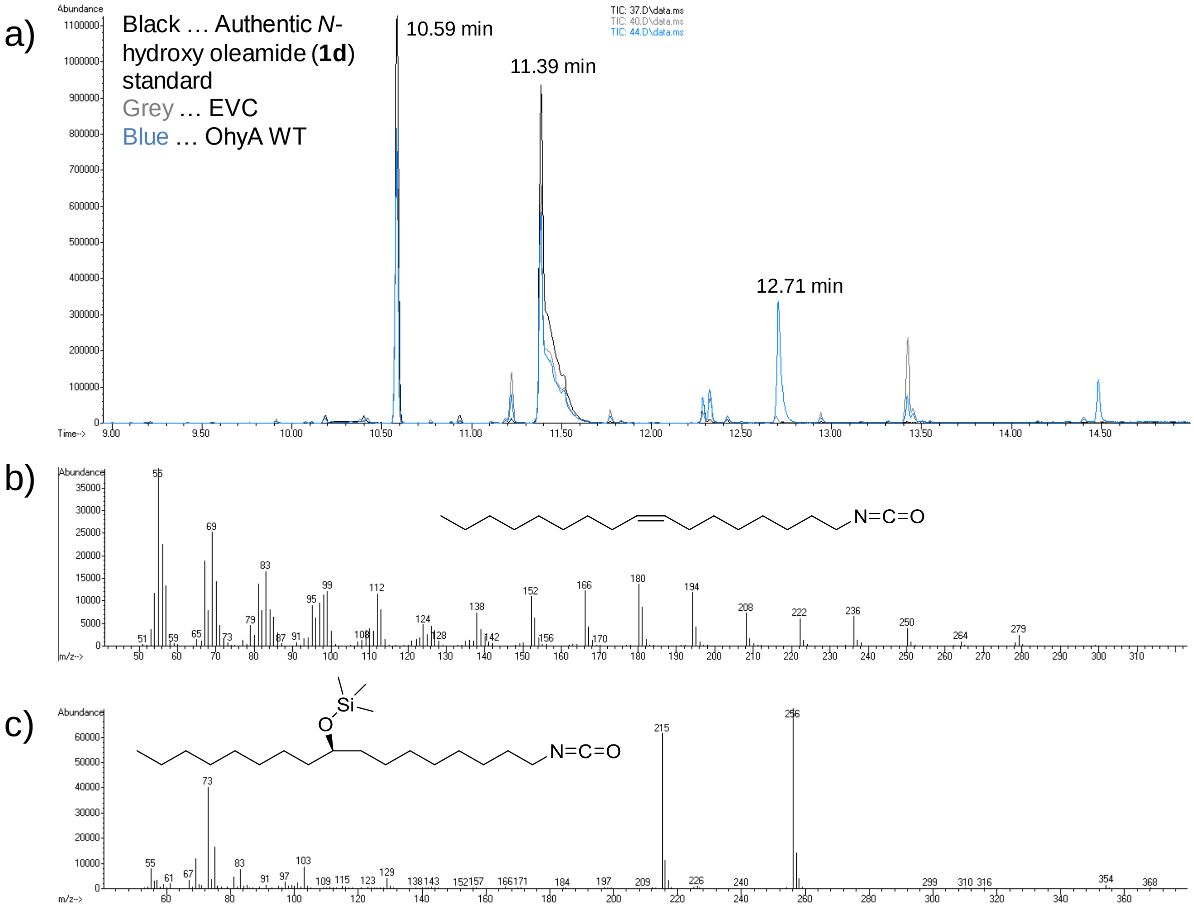

Figure S4. Bioconversion of *N*-hydroxy oleamide (**1d**) to 2d with *E. coli* whole cells over-expressing OhyA wild type enzyme. Due to thermally induced Lossen rearrangement, only the isocyanates were detected in the GC-MS. a) Overlay of representative GC-MS chromatograms from technical triplicates of an authentic **1d** standard and biotransformations of **1d** with an *E. coli* empty vector control (EVC) and an *E. coli* strain over-expressing OhyA wild type. Retention times of the TMS-derivatives of the internal standard *n*-pentadecanoic acid (10.59 min), **1d** (11.39 min) and **2d** (12.71 min) are highlighted. The latter two compounds may exist in the Lossen-rearranged form already during the chromatography. b) Mass spectrum of the peak at 11.39 min, corresponding to **1d** degraded to (*Z*)-1-isocyanatoheptadec-8-ene by Lossen rearrangement. c) Mass spectrum of the peak at 12.71 min, corresponding to the TMS-derivative of 1-isocyanatoheptadecan-9-ol after Lossen rearrangement of silylated **2d**.

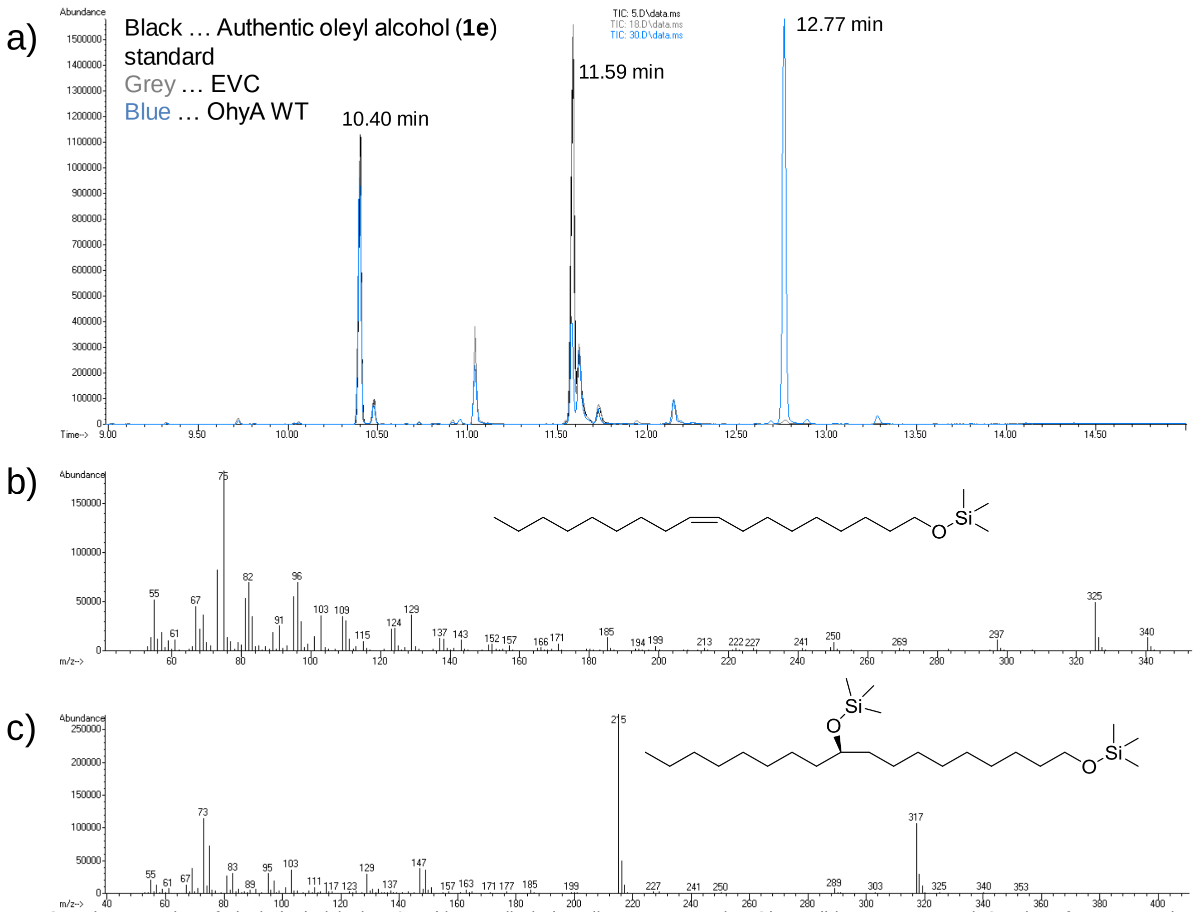

Figure S5. Bioconversion of oleyl alcohol (**1e**) to **2e** with *E. coli* whole cells over-expressing OhyA wild type enzyme. a) Overlay of representative GC-MS chromatograms from technical triplicates of an authentic **1e** standard and biotransformations of **1e** with an *E. coli* empty vector control (EVC) and an *E. coli* strain over-expressing OhyA wild type. Retention times of the TMS-derivatives of the internal standard *n*-pentadecanoic acid (10.40 min), **1e** (11.59 min) and **2e** (12.77 min) are highlighted. b) Mass spectrum of the peak at 11.59 min, corresponding to the TMS-derivative of **1e**. c) Mass spectrum of the peak at 12.77 min, corresponding to the TMS-derivative of **2e**.

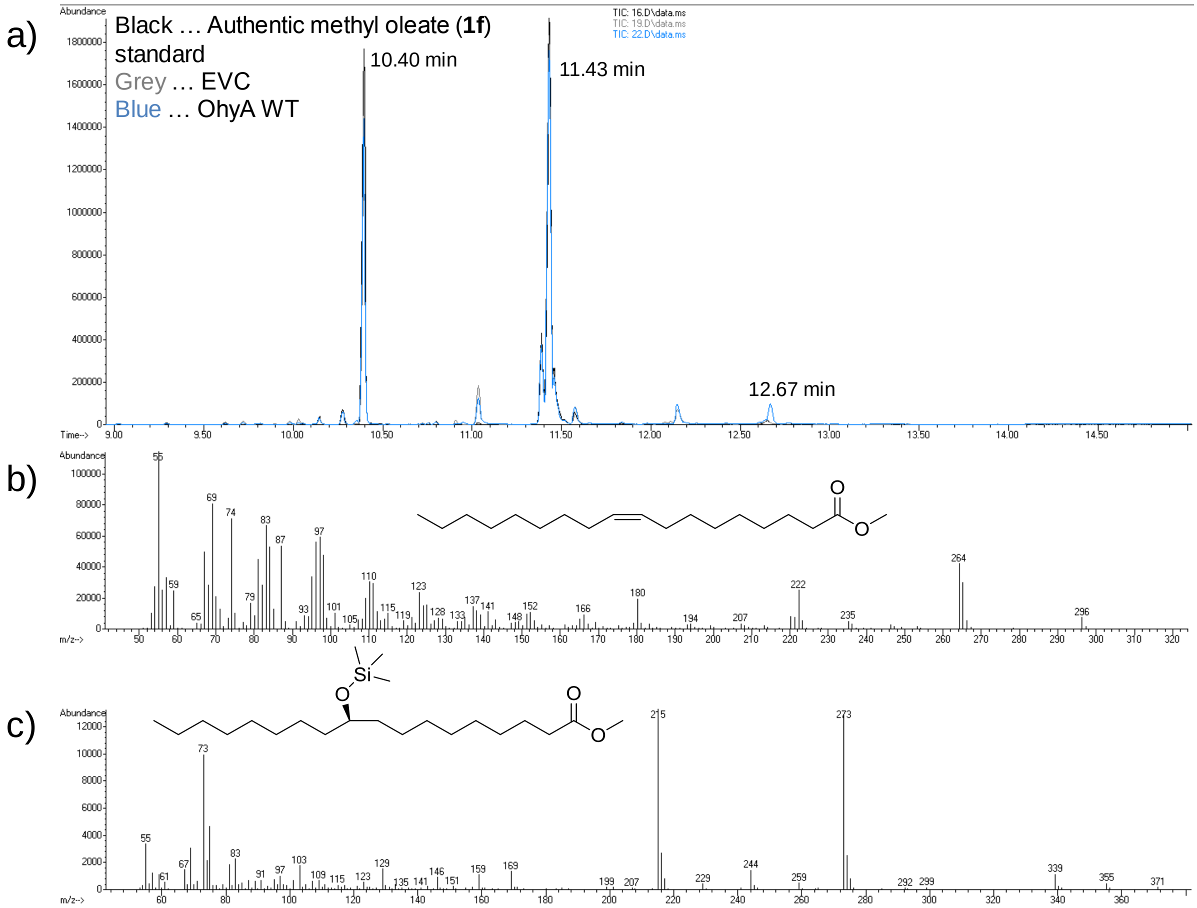

Figure S6. Bioconversion of methyl oleate (**1f**) to **2f** with *E. coli* whole cells over-expressing OhyA wild type enzyme. a) Overlay of representative GC-MS chromatograms from technical triplicates of an authentic **1f** standard and biotransformations of **1f** with an *E. coli* empty vector control (EVC) and an *E. coli* strain over-expressing OhyA wild type. Retention times of the TMS-derivative of the internal standard *n*-pentadecanoic acid (10.40 min), **1f** (11.43 min) and the TMS-derivative of **2f** (12.67 min) are highlighted. b) Mass spectrum of the peak at 11.43 min, corresponding to **1f**. c) Mass spectrum of the peak at 12.67 min, corresponding to the TMS-derivative of **2f**.

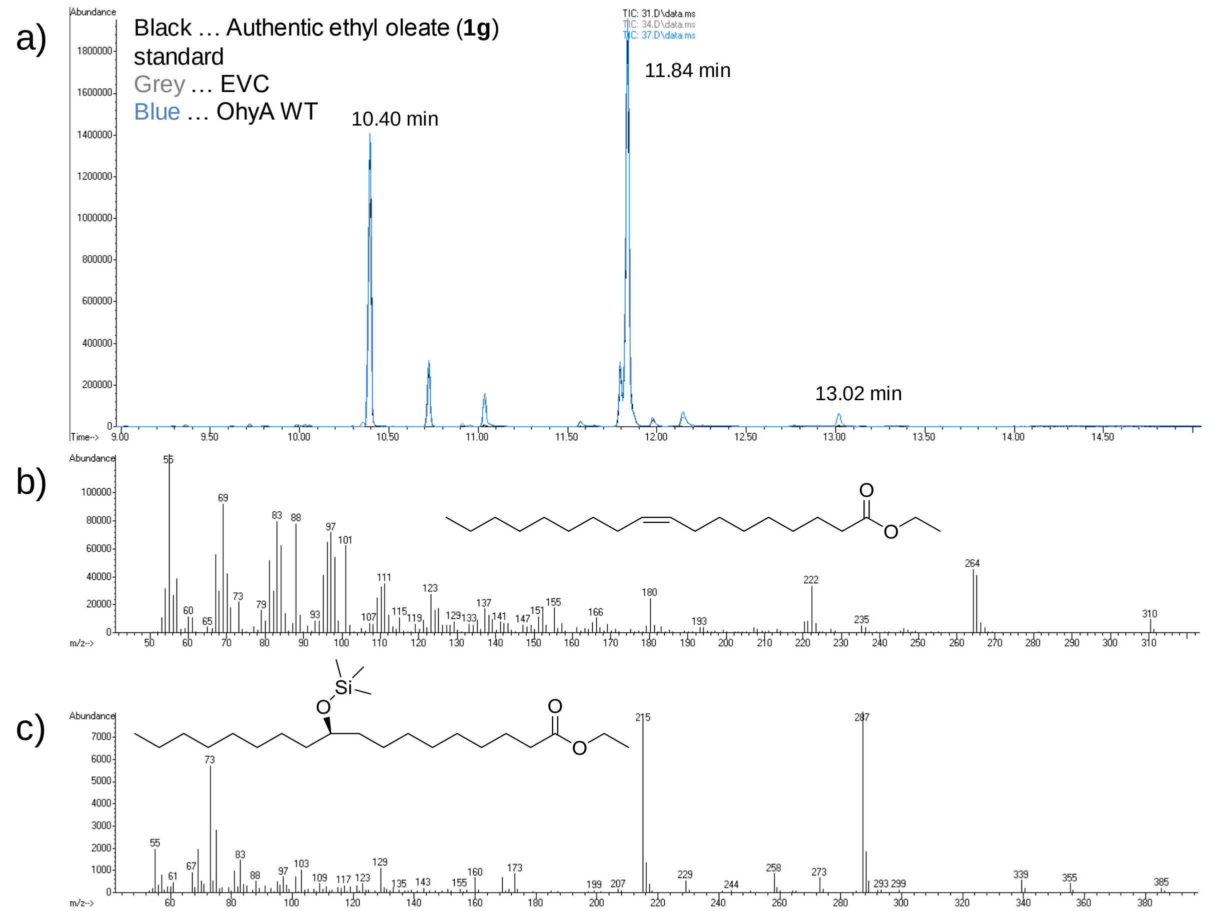

Figure S7. Bioconversion of ethyl oleate (**1g**) to **2g** with *E. coli* whole cells over-expressing OhyA wild type enzyme. a) Overlay of representative GC-MS chromatograms from technical triplicates of an authentic **1g** standard and biotransformations of **1g** with an *E. coli* empty vector control (EVC) and an *E. coli* strain over-expressing OhyA wild type. Retention times of the TMS-derivative of the internal standard *n*-pentadecanoic acid (10.40 min), **1g** (11.84 min) and the TMS-derivative of **2g** (13.02 min) are highlighted. b) Mass spectrum of the peak at 11.84 min, corresponding to **1g**. c) Mass spectrum of the peak at 13.02 min, corresponding to the TMS-derivative of **2g**.

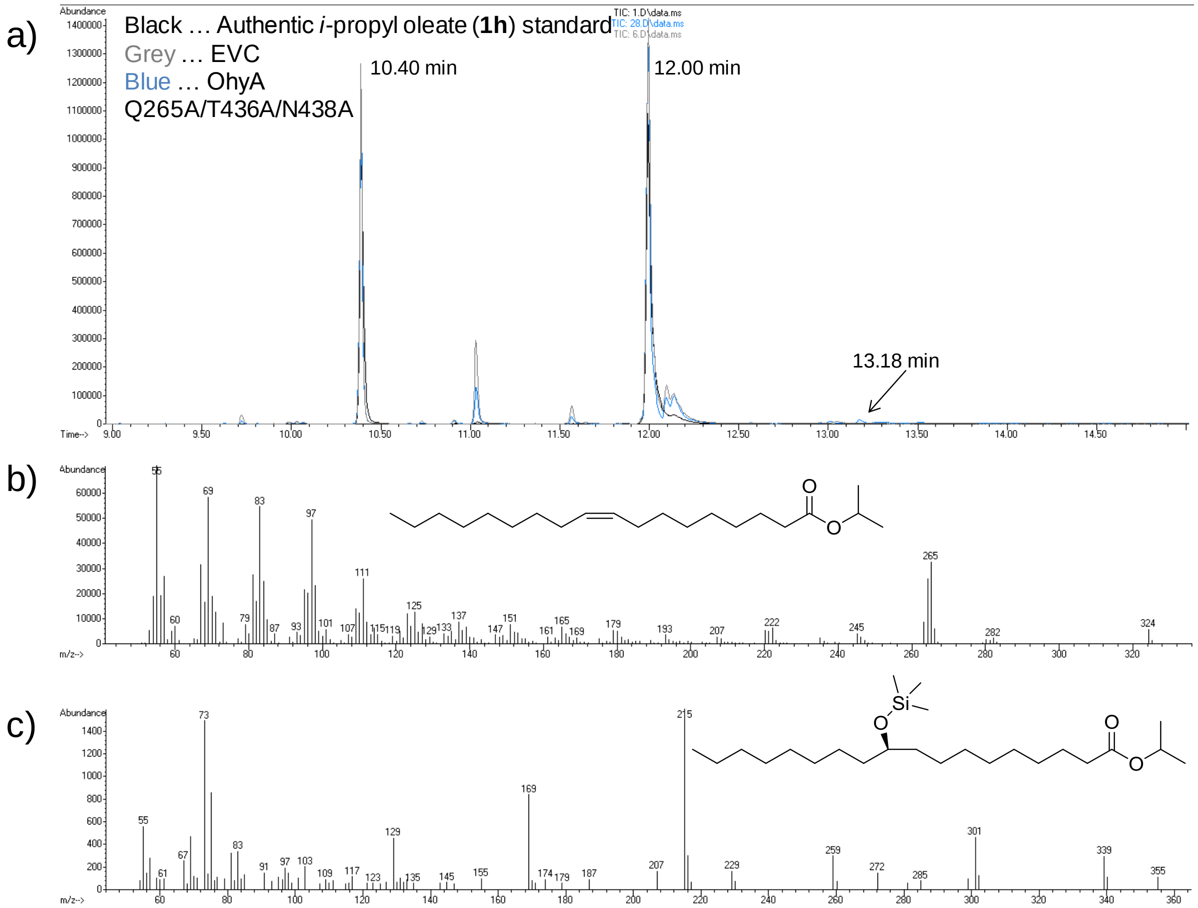

Figure S8. Bioconversion of *i*-propyl oleate (**1h**) to **2h** with *E. coli* whole cells over-expressing OhyA Gln265Ala/Thr436Ala/Asn438Ala. a) Overlay of representative GC-MS chromatograms from technical triplicates of an authentic **1h** standard and biotransformations of **1h** with an *E. coli* empty vector control (EVC) and an *E. coli* strain over-expressing OhyA Gln265Ala/Thr436Ala/Asn438Ala. Retention times of the TMS-derivative of the internal standard *n*-pentadecanoic acid (10.40 min), **1h** (12.00 min) and the TMS-derivative of **2h** (13.18 min) are highlighted. b) Mass spectrum of the peak at 12.00 min, corresponding to **1h**. c) Mass spectrum of the peak at 13.18 min, corresponding to the TMS-derivative of **2h**.

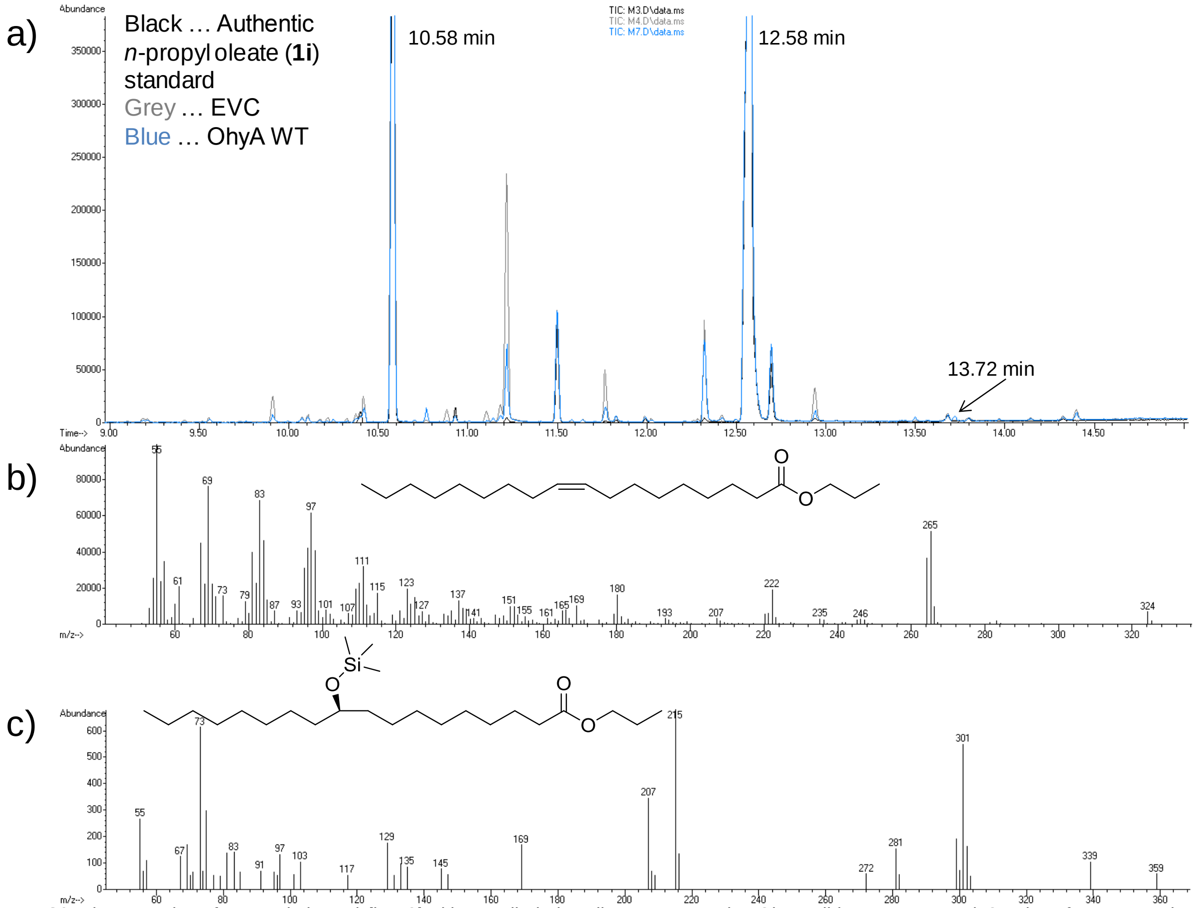

Figure S9. Bioconversion of *n*-propyl oleate (**1i**) to **2i** with *E. coli* whole cells over-expressing OhyA wild type enzyme. a) Overlay of representative GC-MS chromatograms from technical triplicates of an authentic **1i** standard and biotransformations of **1i** with an *E. coli* empty vector control (EVC) and an *E. coli* strain over-expressing OhyA wild type. Retention times of the TMS-derivative of the internal standard *n*-pentadecanoic acid (10.58 min), **1i** (12.58 min) and the TMS-derivative of **2i** (13.72 min) are highlighted. b) Mass spectrum of the peak at 12.58 min, corresponding to **1i**. c) Mass spectrum of the peak at 13.72 min, corresponding to the TMS-derivative of **2i**.

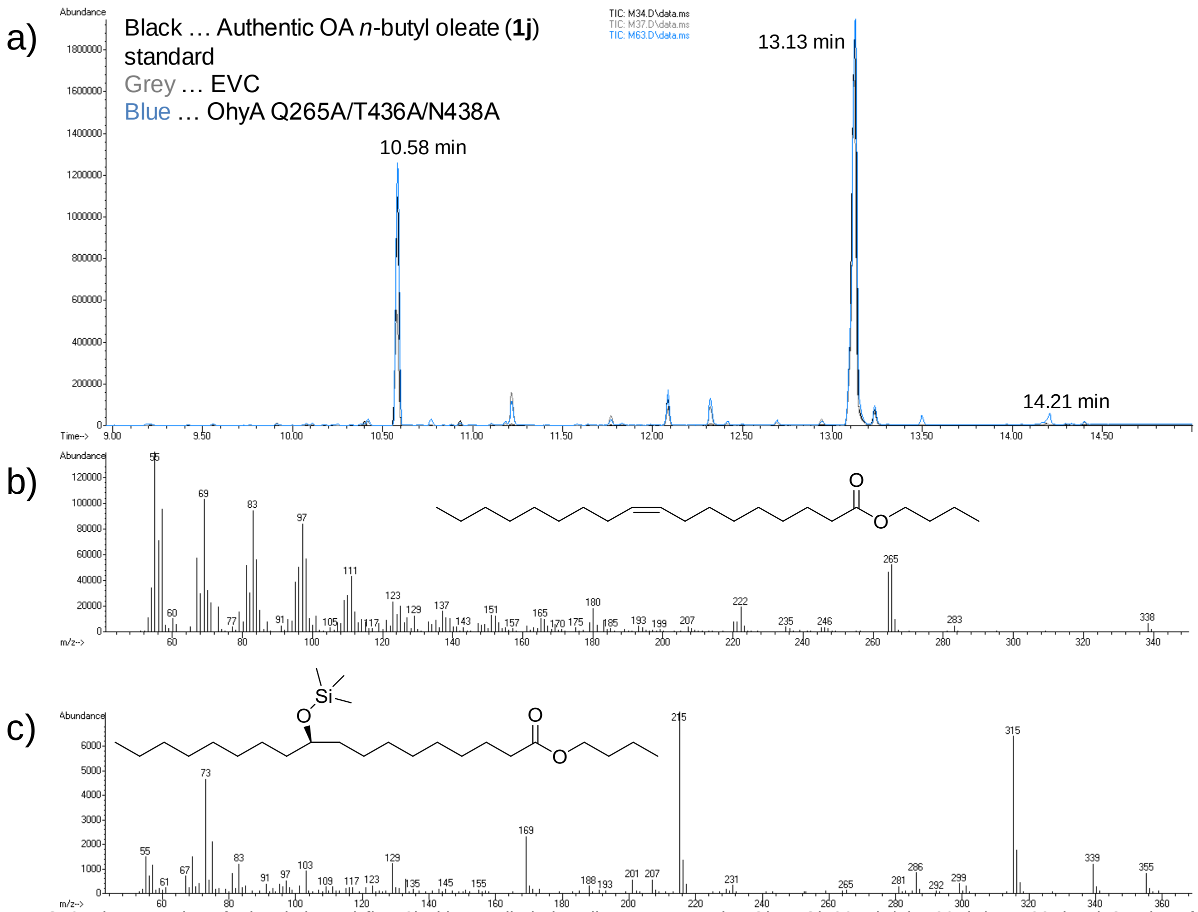

Figure S10. Bioconversion of *n*-butyl oleate (**1j**) to 2j with *E. coli* whole cells over-expressing OhyA Gln265Ala/Thr436Ala/Asn438Ala. a) Overlay of representative GC-MS chromatograms from technical triplicates of an authentic **1j** standard and biotransformations of **1j** with an *E. coli* empty vector control (EVC) and an *E. coli* strain over-expressing OhyA Gln265Ala/Thr436Ala/Asn438Ala. Retention times of the TMS-derivative of the internal standard *n*-pentadecanoic acid (10.58 min), **1j** (13.13 min) and the TMS-derivative of **2j** (14.21 min) are highlighted. b) Mass spectrum of the peak at 13.13 min, corresponding to **1j**. c) Mass spectrum of the peak at 14.21 min, corresponding to the TMS-derivative of **2j**.

Spectroscopic and optical data of purified reaction products

Compound **2a**: (*R*)-10-hydroxy stearic acid (analyzed as methyl ester and after esterification of the 10-hydroxy group with (*S*)-(+)-*O*-acetylmandelic acid) (see lit.[2,11]).

^{1}H-NMR (500 MHz, $CDCl_3$): δ = 0.87 (3H, t, ^3J(H,H) = 7.2 Hz, H-18), 0.99-1.33 (24H, m, 12 CH_2], 1.33-1.40 (2H, dd, ^3J(H,H) = 14.8 Hz, 7.0 Hz, H-9 or H-11), 1.58-1.65 (2H, m, H-3 or H-4), 2.19 (3H, s, CH_3CO), 2.30 (2H, t, ^3J(H,H) = 7.5 Hz, H-2), 3.6646 (3H, s, OCH_3), 4.87 (1H, p, ^3J(H,H) = 6.2 Hz, H-10), 5.87 (1H, s, H-2'), 7.33-7.39 (3H, m, H-3", H-4", H-5"), 7.47 (2H, dd, ^3J(H,H) = 7.2 Hz, ^4J(H,H) = 2.0 Hz, H-2", H-6").

^{13}C-NMR (125 MHz, $CDCl_3$): δ = 14.24, 20.87, 22.78, 24.88, 25.09, 25.26, 29.26 (3C), 29.40, 29.42, 29.49, 29.51, 31.99, 34.07, 34.26, 34.29, 51.55, 74.92, 76.14, 127.75, 128.77, 129.22, 134.35, 168.85, 170.36, 174.44.

Compound **2c**: 10-hydroxy octadecanamide

^{1}H-NMR (300 MHz, $CDCl_3$): δ = 0.88 (3H, t, ^3J(H,H) = 6.6 Hz, Me), 1.15-1.45 (23H, m, 11 CH_2, -CH-O$\underline{H}$), 1.45-1.72 (6H, m, -C$\underline{H}_2$-CH(OH)-C$\underline{H}_2$-, -C$\underline{H}_2$-CH_2- CO_2NH_2), 2.22 (2H, t, ^3J(H,H) = 7.5 Hz, -C$\underline{H}_2$-CO_2NH_2), 3.58 (1H, m, >C$\underline{H}$-OH), 5.35 (2H, br, -CO_2N$\underline{H}_2$).
^{13}C-NMR (75 MHz, $CDCl_3$): δ = 14.25, 22.82, 25.63, 25.74, 25.82, 29.32, 29.36, 29.43, 29.53, 29.74 (2C), 29.87, 32.03, 36.03, 37.60, 37.69, 72.16, 175.56.

$[\alpha]_D^{25}$ = -4.0 ° (c = 0.15 in $CHCl_3$)

Compound **2d**: *N*,10-dihydroxyoctadecanamide
^{1}H-NMR (300 MHz, $CDCl_3$): δ = 0.88 (3H, t, ^3J(H,H) = 6.7 Hz, Me), 1.05-1.40 (22H, m, 11 CH_2), 1.40-1.53 (4H, m, -C$\underline{H}_2$-CH(OH)-C$\underline{H}_2$-), 1.56-1.72 (3H, m, -C$\underline{H}_2$-CH_2-CO_2NHOH, -CH-O$\underline{H}$), 2.17 (2H, t, ^3J(H,H) = 7.1 Hz, -C$\underline{H}_2$-CO_2NHOH), 3.58 (1H, m, >C$\underline{H}$-OH), 4.37 (2H, d, ^3J(H,H) = 7.0 Hz, -CO_2N$\underline{H}$O$\underline{H}$).

^{13}C-NMR (75 MHz, $CDCl_3$): δ = 14.25, 22.82, 25.24, 25.54, 25.81, 28.98, 29.21, 29.41, 29.49, 29.71, 29.73, 29.86, 32.04, 35.99, 37.46, 37.68, 72.25, 175.41.

$[\alpha]_D^{25}$ = -7.9 ° (c = 0.1 in $CHCl_3$)

Compound **2e**: 1,10-octadecanediol

^{1}H-NMR (300 MHz, $CDCl_3$): δ = 0.88 (3H, t, ^3J(H,H) = 6.8 Hz, Me), 1.15-1.50 (30H, m, 12 CH_2, -C$\underline{H}_2$-CH(OH)-C$\underline{H}_2$-, 2× OH), 1.56 (2H, m, -C$\underline{H}_2$-CH_2OH), 3.58 (1H, m, >C$\underline{H}$-OH), 3.64 (2H, t, ^3J(H,H) = 6.6 Hz, -C$\underline{H}_2$OH).

^{13}C-NMR (75 MHz, $CDCl_3$): δ = 14.25, 22.82, 25.79, 25.81, 25.87, 29.43, 29.55, 29.68 (2C), 29.75, 29.83, 29.87, 32.03, 32.95, 37.63, 37.67, 63.23, 72.18.

$[\alpha]_D^{25}$ = -0.5 ° (c = 0.3 in $CHCl_3$)

Compound **2f**: 10-hydroxy stearic acid acid methyl ester

^{1}H-NMR (300 MHz, $CDCl_3$): δ = 0.87 (3H, t, ^3J(H,H) = 6.3 Hz, Me), 1.15-1.39 (22H, m, 11 CH_2), 1.39-1.52 (4H, m, -C$\underline{H}_2$-CH(OH)-C$\underline{H}_2$-), 1.52-1.69 (3H, t+m, ^3J(H,H) = 7.2 Hz, -C$\underline{H}_2$-CH_2-COOMe, -CH-O$\underline{H}$), 2.30 (2H, t, ^3J(H,H) = 7.5 Hz, -C$\underline{H}_2$-CO_2Me), 3.58 (1H, m, >C$\underline{H}$-OH), 3.66 (3H, s, -CO_2Me).

^{13}C-NMR (75 MHz, $CDCl_3$): δ = 14.25, 22.82, 25.09, 25.76, 25.81, 29.27, 29.33, 29.43, 29.55, 29.75, 29.76, 29.87, 32.04, 34.26, 37.62, 37.67, 51.59, 72.16, 174.47.

$[\alpha]_D^{25}$ = -1.0 ° (c = 0.35 in $CHCl_3$)

Compound **2g**: 10-hydroxy stearic acid acid ethyl ester

^{1}H-NMR (300 MHz, $CDCl_3$): δ = 0.87 (3H, t, ^3J(H,H) = 6.0 Hz, Me), 1.15-1.39 (25H, m, -CO_2CH_2-$\underline{Me}$, 11 CH_2), 1.40-1.50 (4H, m, -C$\underline{H}_2$-CH(OH)-C$\underline{H}_2$-), 1.51-1.68 (3H, t+m, ^3J(H,H) = 6.8 Hz, -C$\underline{H}_2$-CH_2-CO_2Et, -CH-O$\underline{H}$), 2.28 (2H, t, ^3J(H,H) = 7.5 Hz, -C$\underline{H}_2$-CO_2Et), 3.58 (1H, m, >C$\underline{H}$-OH), 4.12 (2H, t, ^3J(H,H) = 7.1 Hz, -CO_2C$\underline{H}_2$-Me).

^{13}C-NMR (75 MHz, $CDCl_3$): δ = 14.24, 14.40, 22.82, 25.11, 25.76, 25.81, 29.26, 29.34, 29.43, 29.55, 29.74, 29.77, 29.87, 32.03, 34.53, 37.62, 37.67, 60.30, 72.15, 174.04.

$[\alpha]_D^{25}$ = -0.7 ° (c = 0.3 in $CHCl_3$)

Compound **2h**: 10-hydroxy stearic acid acid *i*-propyl ester

^{1}H-NMR (300 MHz, $CDCl_3$): δ = 0.88 (3H, t, ^3J(H,H) = 6.6 Hz, Me), 1.12-1.37 (28H, m, 11 CH_2, -CO_2CH$\underline{Me}_2$), 1.37-1.48 (4H, m, -C$\underline{H}_2$-CH(OH)-C$\underline{H}_2$-), 1.50-1.67 (3H, m, -C$\underline{H}_2$-CH_2-CO_2CHMe_2, -CH-O$\underline{H}$), 2.25 (2H, t, ^3J(H,H) = 7.4 Hz, -C$\underline{H}_2$-CO_2CHMe_2), 3.58 (1H, m, >C$\underline{H}$-OH), 5.00 (1H, p, ^3J(H,H) = 6.4 Hz, -CO_2CHMe_2).

^{13}C-NMR (75 MHz, $CDCl_3$): δ = 14.25, 22.01 (-CO_2CH$\underline{Me}_2$), 22.82, 25.18, 25.77, 25.81, 29.24, 29.35, 29.43, 29.57, 29.75, 29.78, 29.87, 32.04, 34.87, 37.63, 37.67, 67.48, 72.17, 173.59.

$[\alpha]_D^{25}$ = -3.1 ° (c = 0.1 in $CHCl_3$)

Compound **2i**: 10-hydroxy stearic acid acid *n*-propyl ester

^{1}H-NMR (300 MHz, $CDCl_3$): δ = 0.88 (3H, t, ^3J(H,H) = 6.3 Hz, Me), 0.94 (3H, t, ^3J(H,H) = 7.4 Hz, -$CO_2CH_2CH_2$$\underline{Me}$), 1.15-1.38 (22H, m, 11 CH_2), 1.38-1.48 (4H, m, -C$\underline{H}_2$-CH(OH)-C$\underline{H}_2$-), 1.48-1.67 (3H, m, -C$\underline{H}_2$-CH_2-$CO_2CH_2CH_2$Me, -CH-O$\underline{H}$), 1.65 (2H, sextet, ^3J(H,H) = 7.1 Hz, -CO_2CH_2C$\underline{H}_2$Me), 2.29 (2H, t, ^3J(H,H) = 7.5 Hz, -C$\underline{H}_2$-$CO_2CH_2CH_2$Me), 3.58 (1H, m, >C$\underline{H}$-OH), 4.02 (2H, t, ^3J(H,H) = 6.6 Hz, -CO_2C$\underline{H}_2$$CH_2$Me).

^{13}C-NMR (75 MHz, $CDCl_3$): δ = 10.55, 14.25, 22.17, 22.82, 25.16, 25.77, 25.81, 29.28, 29.35, 29.43, 29.56, 29.75, 29.77, 29.87, 32.04, 34.54, 37.62, 37.67, 65.98, 72.17, 174.16.

$[\alpha]_D^{25}$ = -1.5 ° (c = 0.15 in $CHCl_3$)

Compound **2j**: 10-hydroxy stearic acid acid *n*-butyl ester

^{1}H-NMR (300 MHz, $CDCl_3$): δ = 0.88 (3H, t, ^3J(H,H) = 6.6 Hz, Me), 0.93 (3H, t, ^3J(H,H) = 7.4 Hz, $-CO_2CH_2CH_2CH_2\underline{Me}$), 1.18-1.36 (22H, m, 11 CH_2), 1.36-1.47 (6H, m, $-C\underline{H}_2$-CH(OH)-$C\underline{H}_2$-, $-CO_2CH_2CH_2C\underline{H}_2Me$), 1.50-1.67 (5H, m, $-C\underline{H}_2-CH_2-CO_2CH_2CH_2CH_2Me$, $-CO_2CH_2C\underline{H}_2CH_2Me$, -CH-O$\underline{H}$), 2.29 (2H, t, ^3J(H,H) = 7.4 Hz, $-C\underline{H}_2-CO_2CH_2CH_2CH_2Me$), 3.58 (1H, m, >C$\underline{H}$-OH), 4.07 (2H, t, ^3J(H,H) = 6.5 Hz, $-CO_2C\underline{H}_2CH_2CH_2Me$).

^{13}C-NMR (75 MHz, $CDCl_3$): δ = 13.86, 14.25, 19.31, 22.83, 25.16, 25.77, 25.81, 29.28, 29.35, 29.43, 29.56, 29.75, 29.78, 29.87, 30.87, 32.04, 34.55, 37.62, 37.67, 64.26, 72.17, 174.15.

$[\alpha]_D^{25}$ = -5.1 (c = 0.1 in $CHCl_3$)

NMR spectra of reaction products

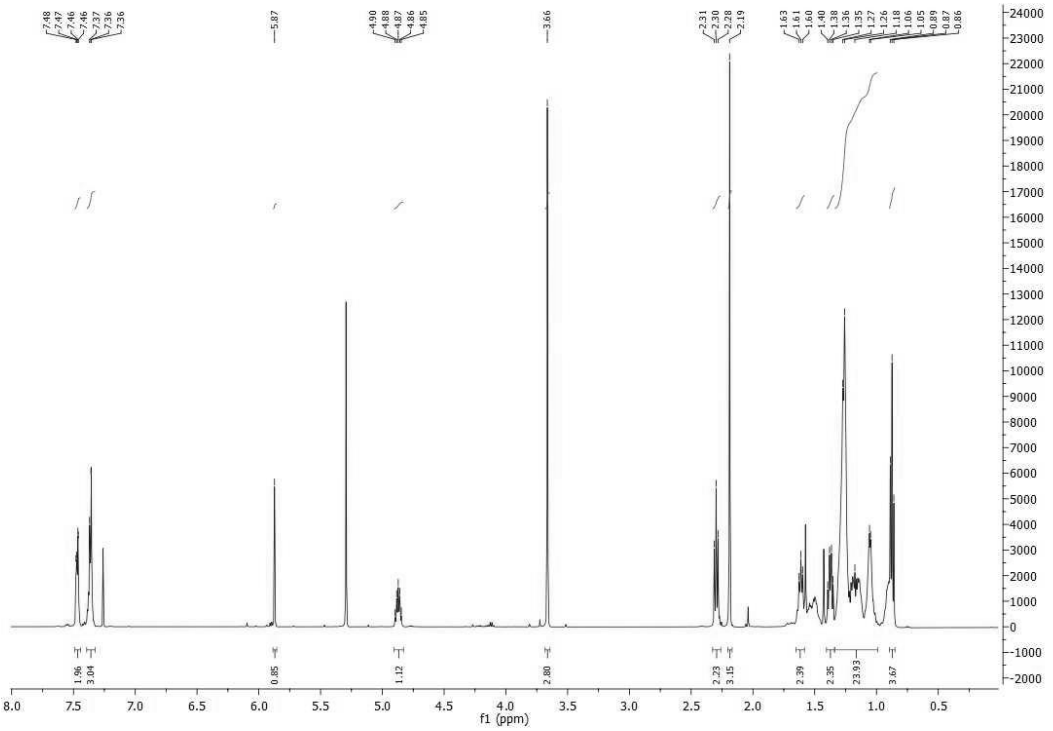

Figure S11. ^{1}H-NMR (500MHz, $CDCl_3$) of **2a** as methyl ester and after esterification of the 10-hydroxy group with (*S*)-(+)-*O*-acetylmandelic acid.

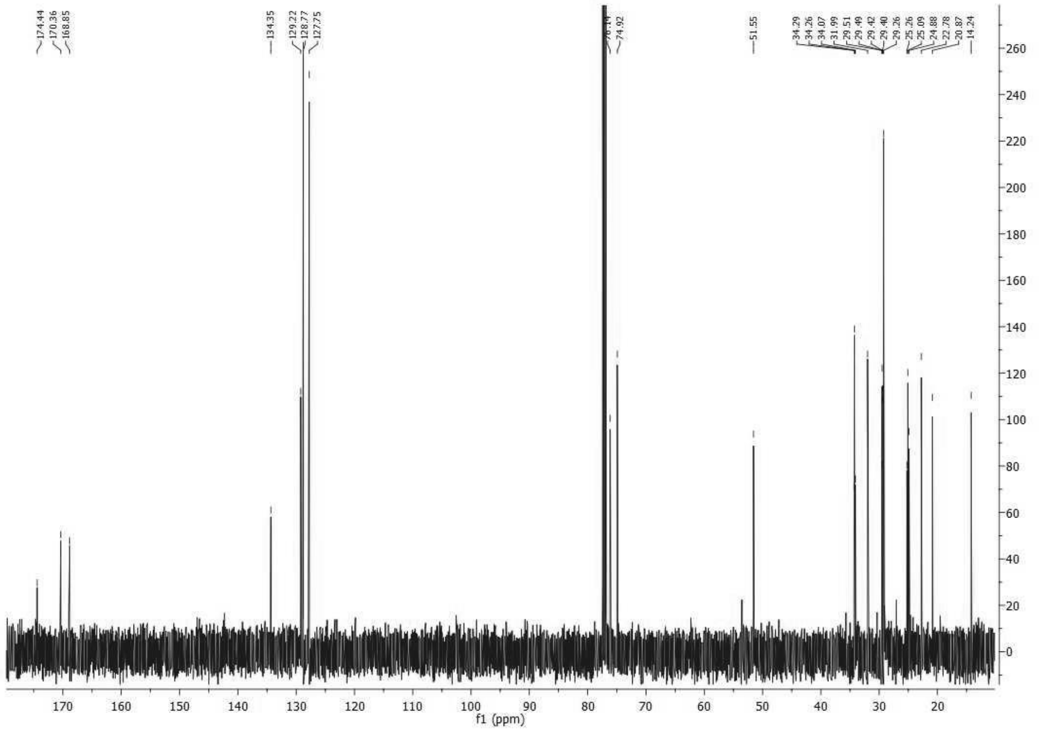

Figure S12. ^{13}C-NMR (125MHz, $CDCl_3$) of **2a** as methyl ester and after esterification of the 10-hydroxy group with (*S*)-(+)-*O*-acetylmandelic acid.

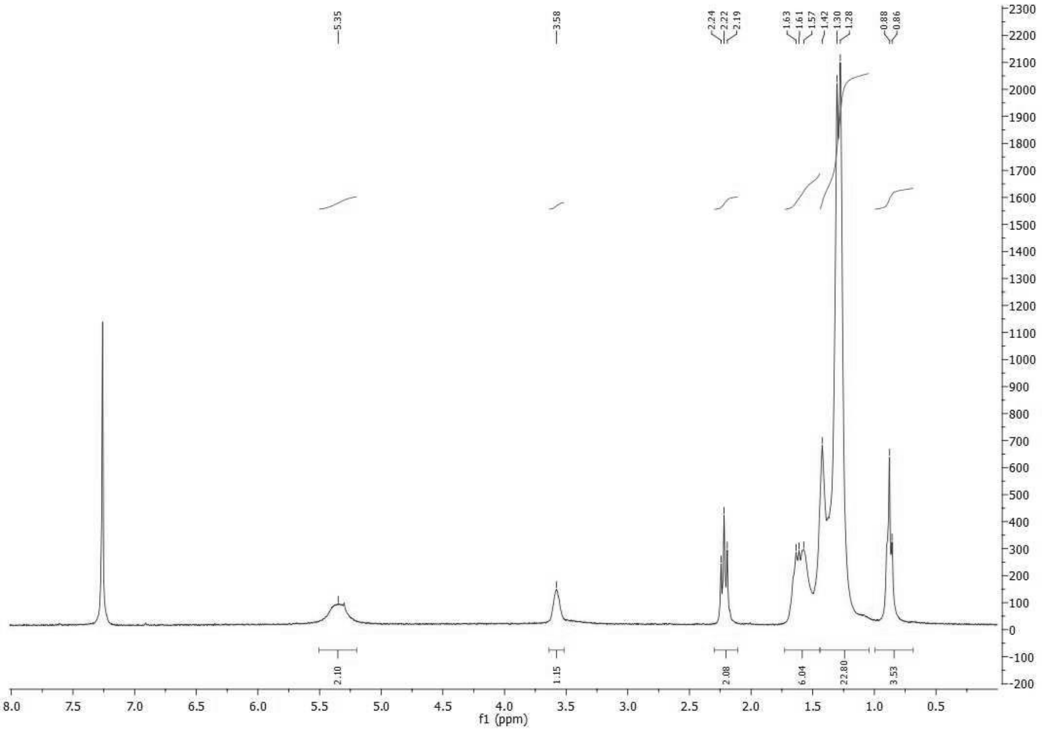

Figure S13. ^{1}H-NMR (300MHz, $CDCl_3$) of **2c**.

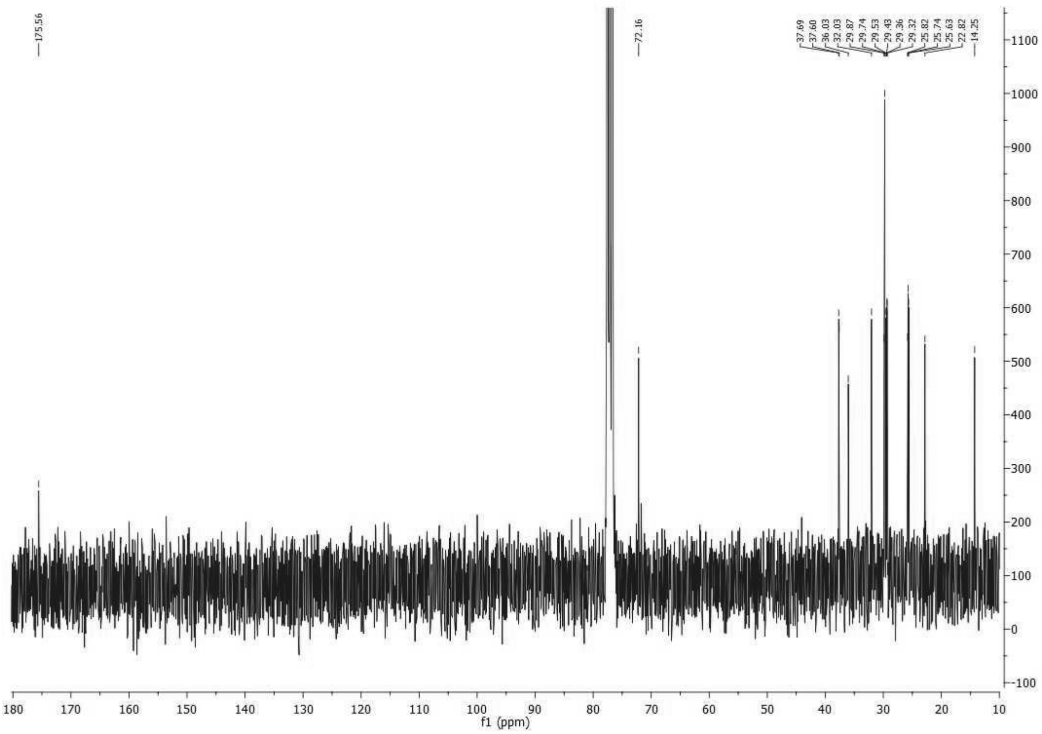

Figure S14. ^{13}C-NMR (75MHz, $CDCl_3$) of **2c**.

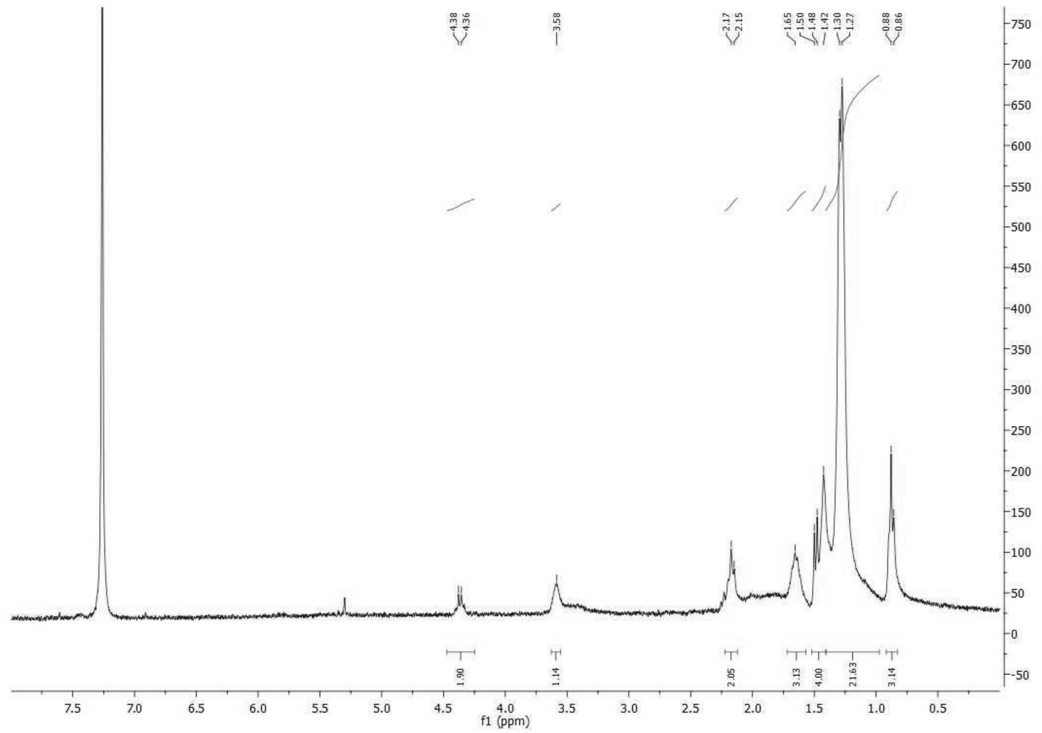

Figure S15. ^{1}H-NMR (300MHz, $CDCl_3$) of **2d**.

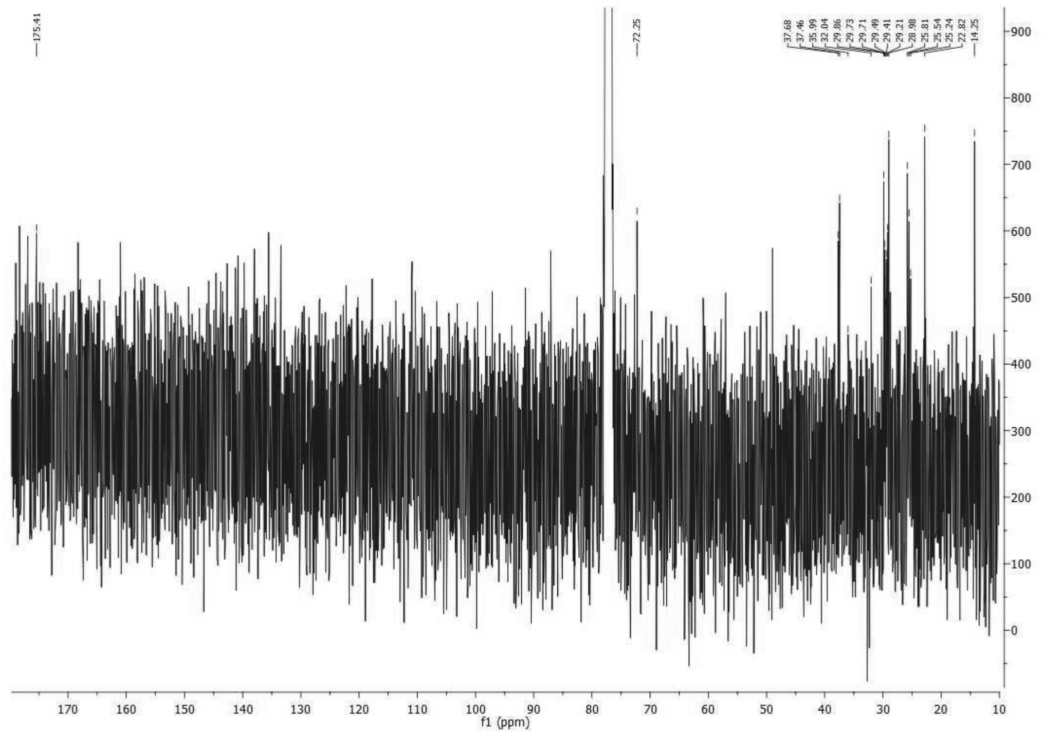

Figure S16. ^{13}C-NMR (75MHz, $CDCl_3$) of **2d**.

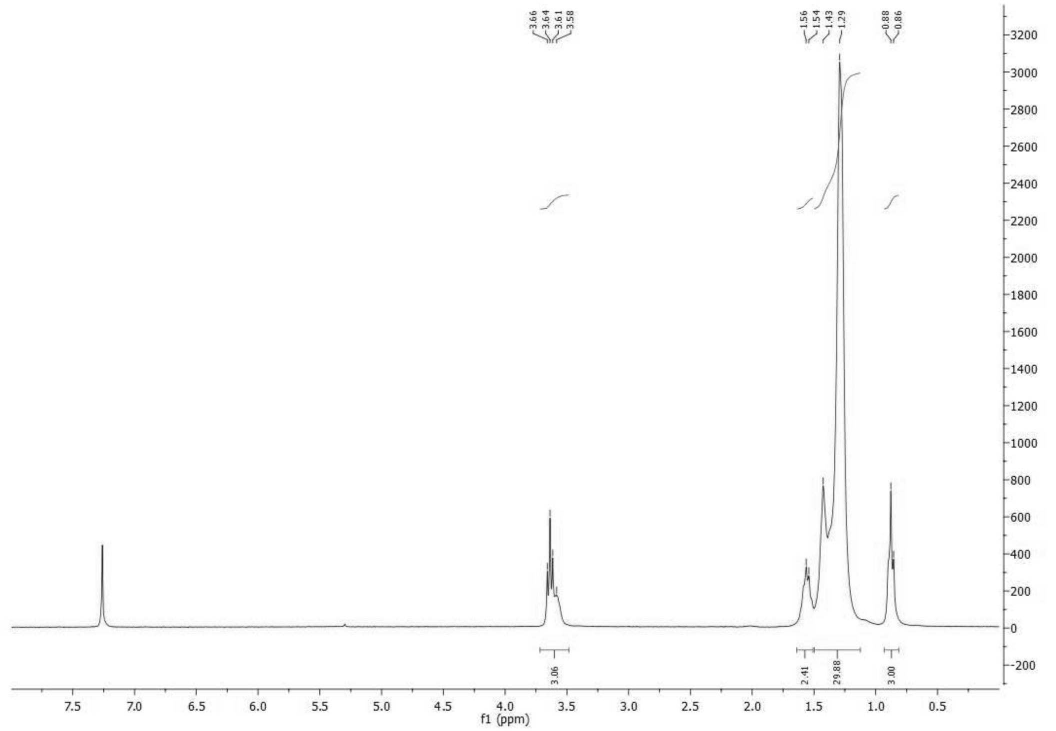

Figure S17. ^{1}H-NMR (300MHz, $CDCl_3$) of **2e**.

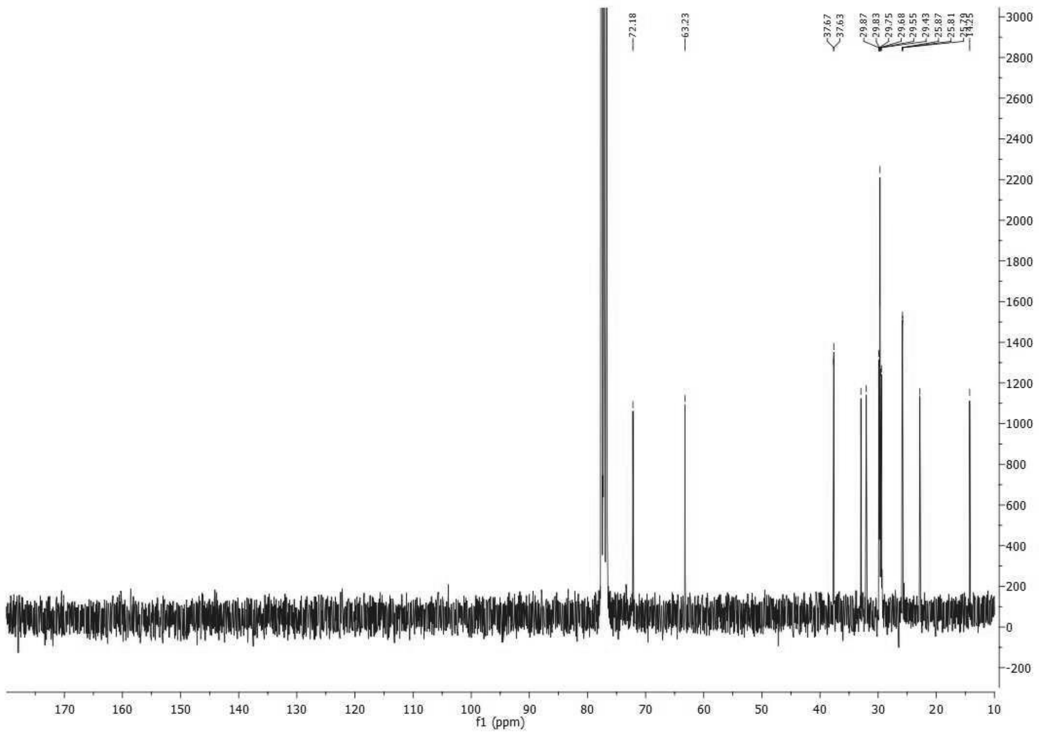

Figure S18. ^{13}C-NMR (75MHz, $CDCl_3$) of **2e**.

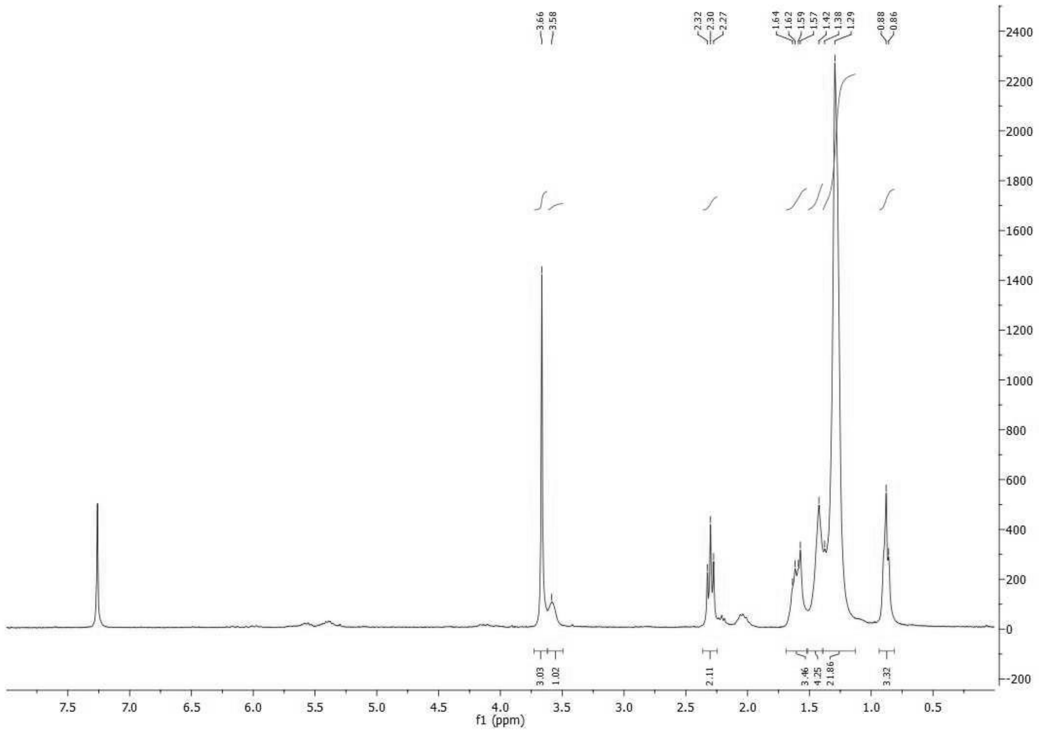

Figure S19. ^{1}H-NMR (300MHz, $CDCl_3$) of **2f**.

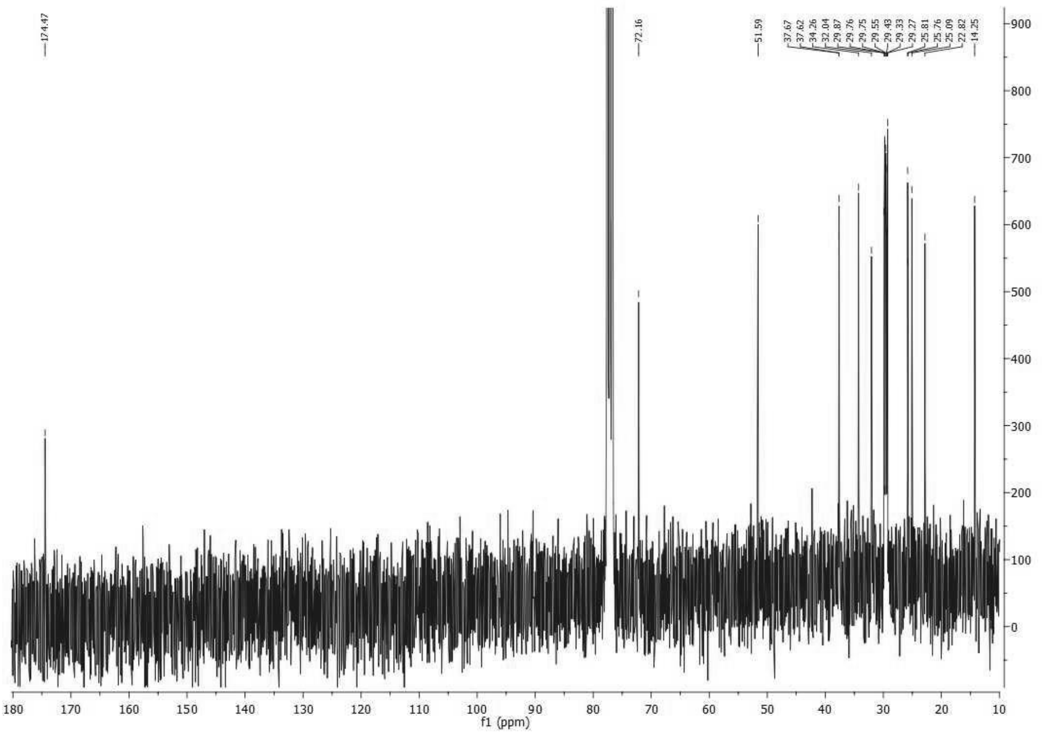

Figure S20. ^{13}C-NMR (75MHz, $CDCl_3$) of **2f**.

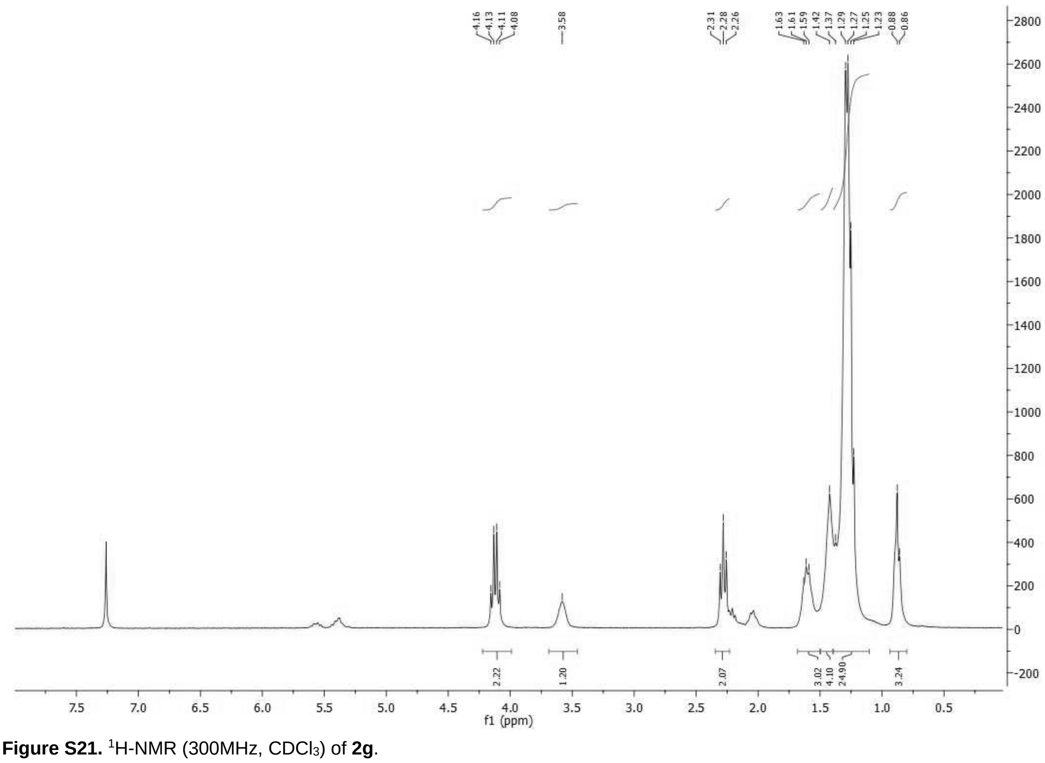

Figure S21. ^{1}H-NMR (300MHz, $CDCl_3$) of **2g**.

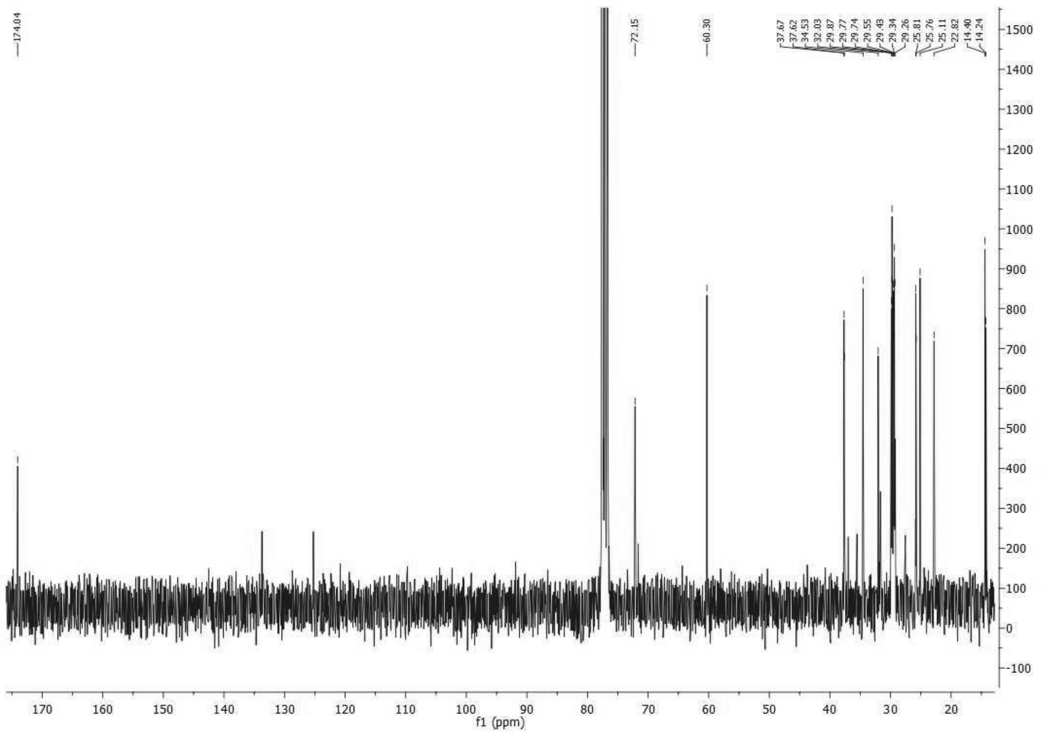

Figure S22. ^{13}C-NMR (75MHz, $CDCl_3$) of **2g**.

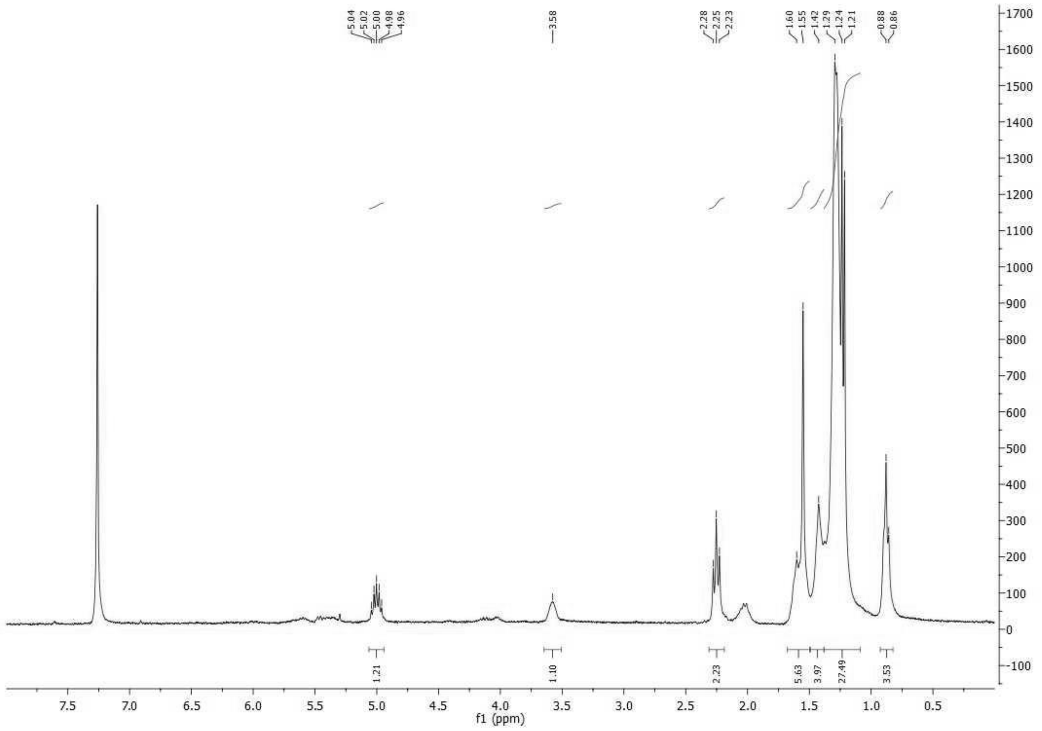

Figure S23. ^{1}H-NMR (300MHz, $CDCl_3$) of **2h**.

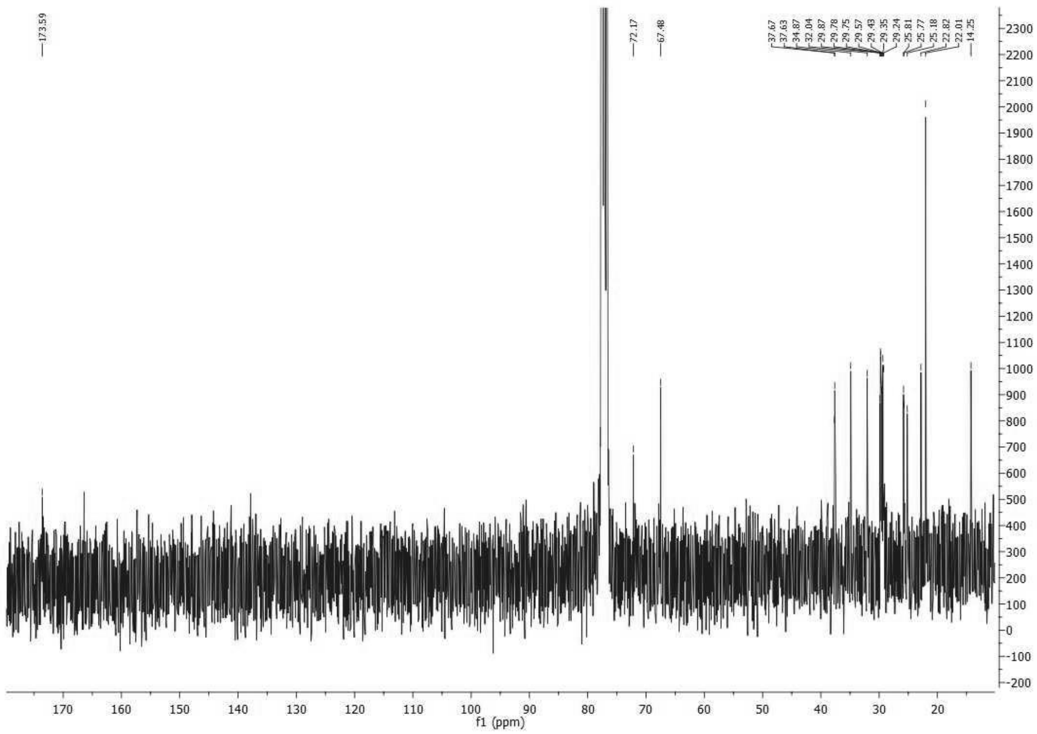

Figure S24. ^{13}C-NMR (75MHz, $CDCl_3$) of **2h**.

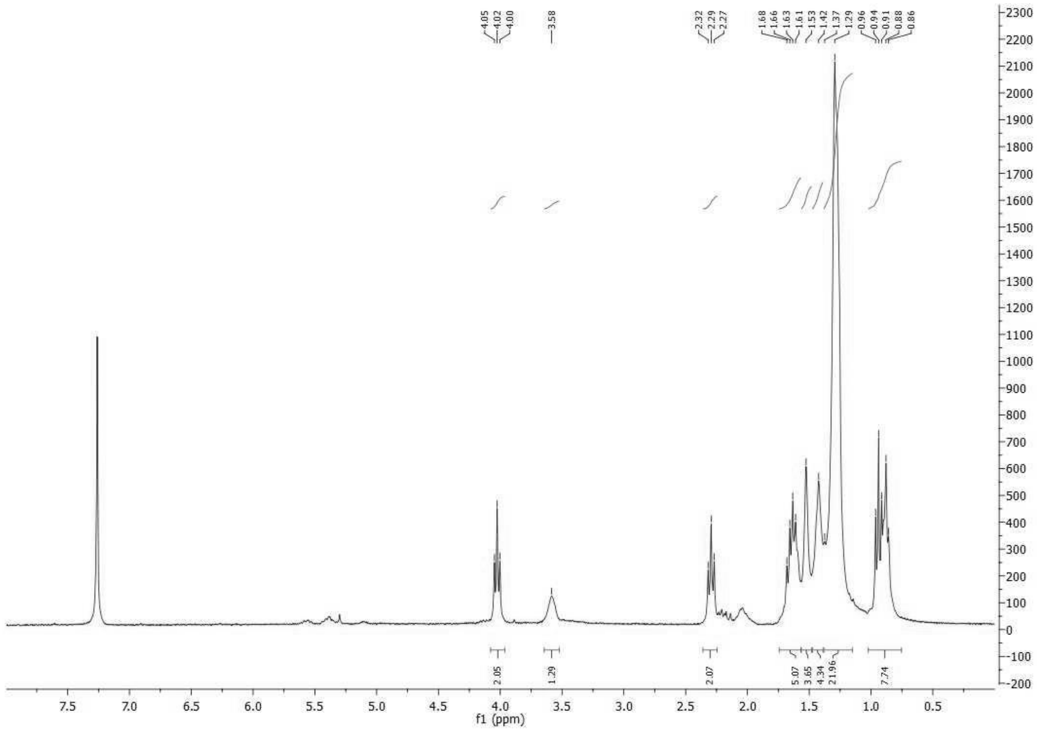

Figure S25. ^{1}H-NMR (300MHz, $CDCl_3$) of **2i**.

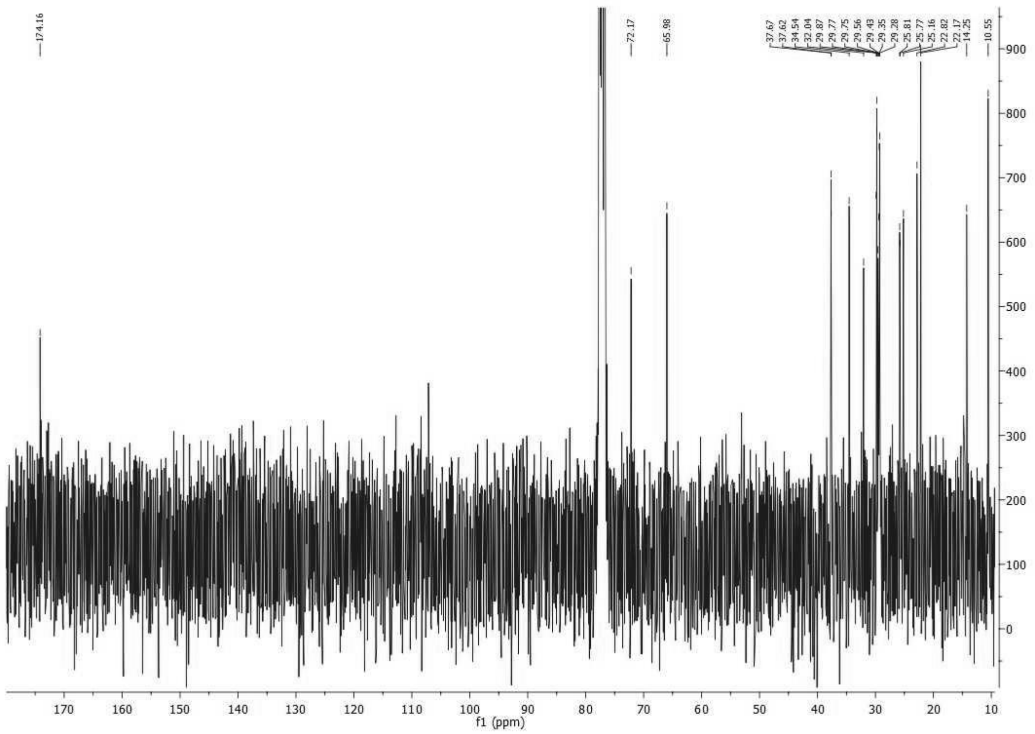

Figure S26. ^{13}C-NMR (75MHz, $CDCl_3$) of **2i**.

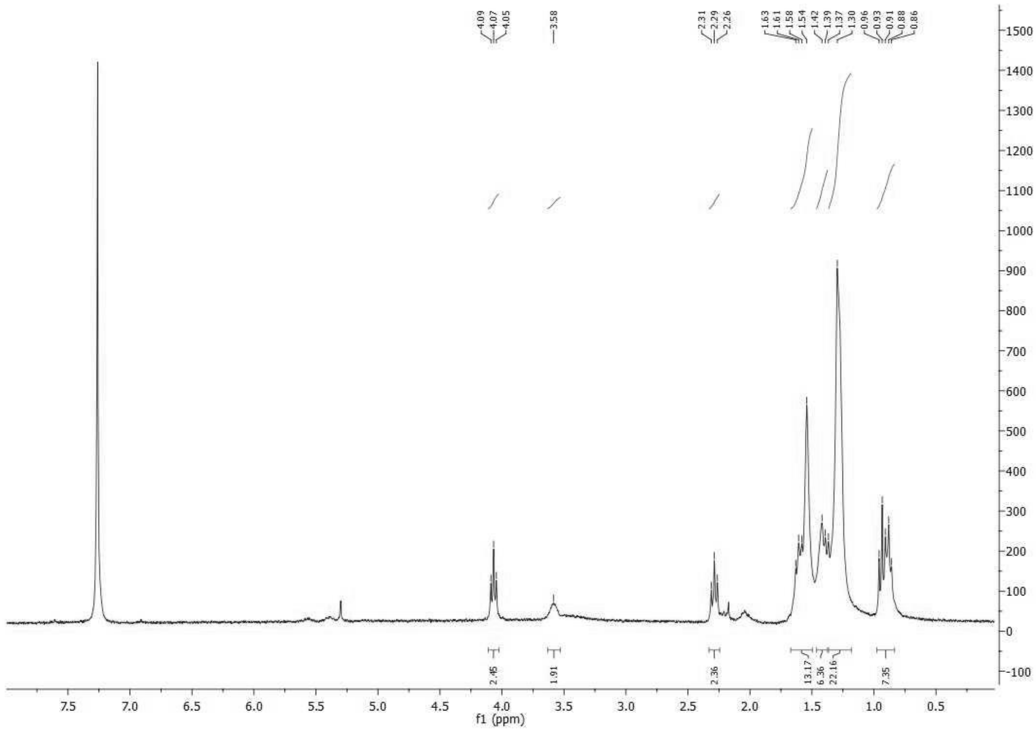

Figure S27. ^{1}H-NMR (300MHz, $CDCl_3$) of **2j**.

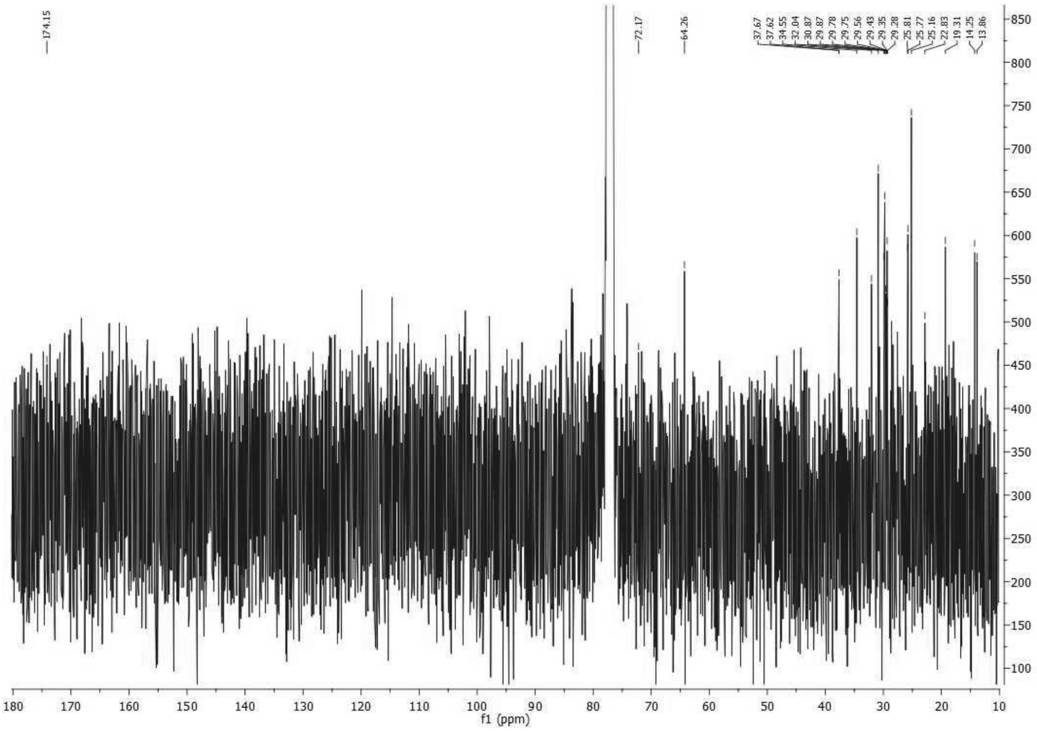

Figure S28. ^{13}C-NMR (75MHz, $CDCl_3$) of **2j**.

Amino acid sequence alignment

To support our selection of amino acid residues involved in substrate binding in OhyA, we performed alignments of the OhyA protein sequence with all sequences of HFam11 in the HyED (Figure S29).[3] The high degree of conservation of Gln265, Thr436, Asn438 and His442 is highlighted by the red boxes, and perfectly in line with our docking and site-directed mutagenesis analyses.[2]

a)

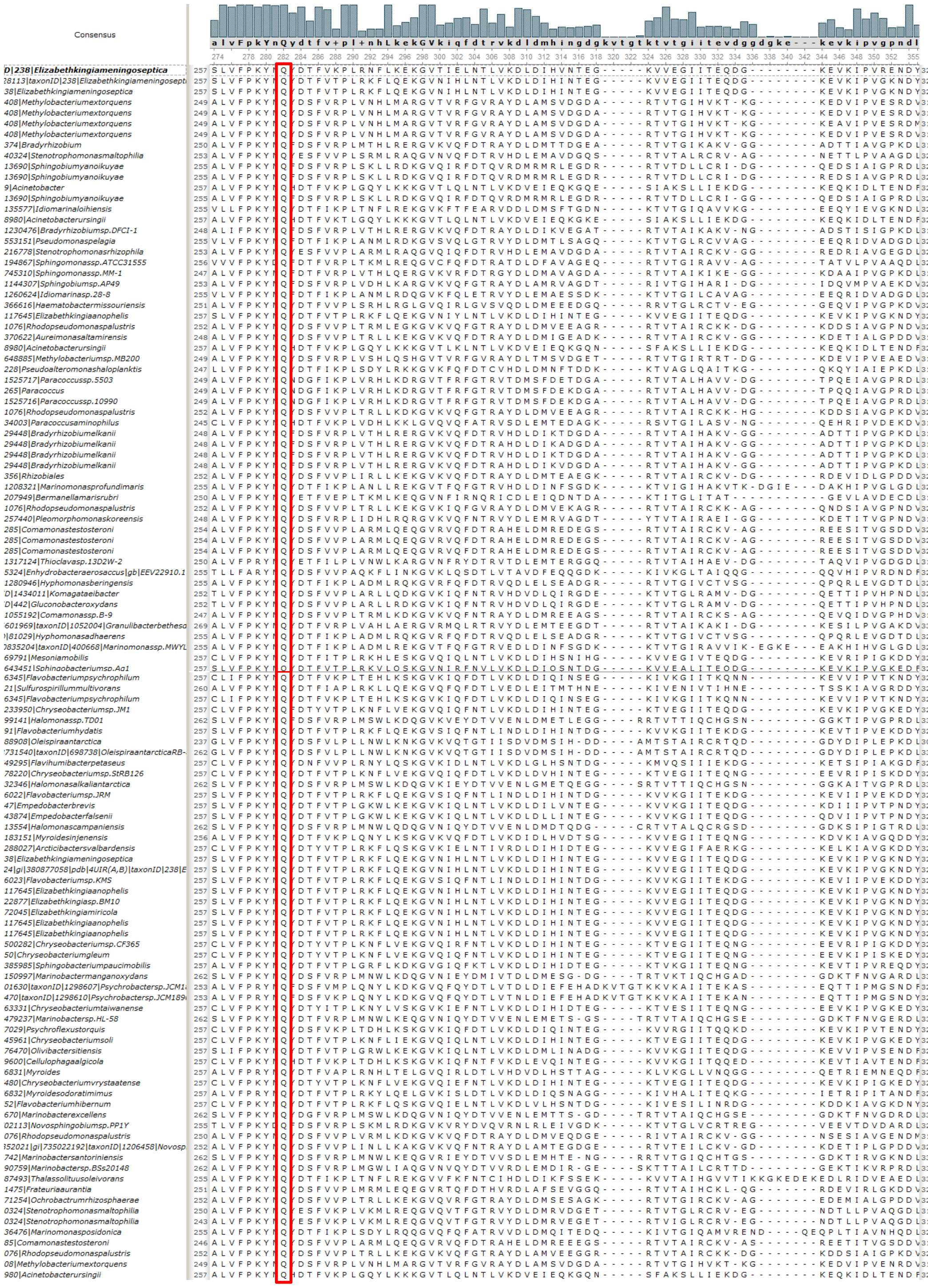

b)

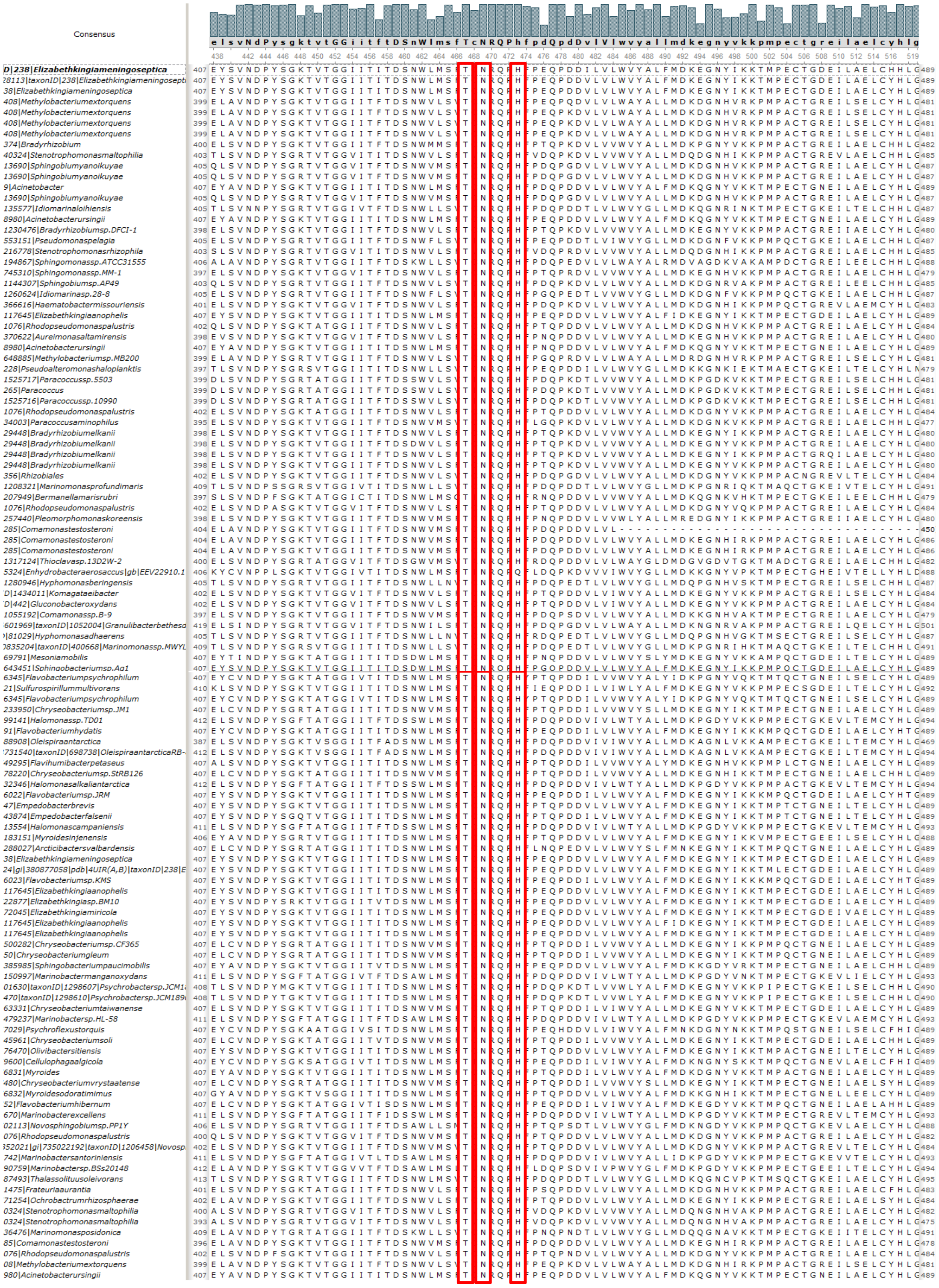

Figure S29. A segment of the multiple sequence alignment of the 116 amino acid sequences from HFam11 collected in the hydratase engineering database (HyED), highlighting the conserved residues involved in binding of a carboxylate (red boxes). a) Gln265 is conserved among all members of HFam11. b) Thr436 and Asn438 are conserved among all members of HFam11, while His442 is conserved among all but one member, where it is substituted with a Gln.

Conversion of OA derivatives with OhyA wild type and substrate binding variants

We compared the activity of OhyA wild type and all solubly expressed variants for hydration of **1a–1j** by performing bioconversions with whole *E. coli* cells for 22 h (Figure S32 - Figure S39).

Table S1. OhyA variants used for conversion of OA derivatives **1b–1j**. The enzymes tested with the different substrates were selected on basis of favoring the interaction between head groups and the substrate binding residues.

Head group	Entry	OhyA variants applied in conversions			
		Single variants	Double variants	Triple variants	Quadruple variants
Amine	**1b**	Gln265Glu; Gln265Ser<br>Thr436Asp<br>Asn438Asp; Asn438Ser<br>His442Asp; His442Glu; His442Tyr	Gln265Glu/Thr436Asp<br>Gln265Glu/Asn438Asp		
Amide	**1c**	Gln265Glu; Gln265Ser<br>Thr436Asp<br>Asn438Asp; Asn438Ser<br>His442Asp; His442Glu; His442Tyr	Gln265Glu/Thr436Asp<br>Gln265Glu/Asn438Asp<br>Gln265Ser/Asn438Asp<br>Gln265Ser/Asn438Ser<br>Thr436Asp/Asn438Asp<br>Thr436Asp/Asn438Ser	Gln265Glu/Thr436Asp/Asn438Asp<br>Gln265Ser/Thr436Asp/Asn438Asp	
Hydroxamic acid (*N*-hydroxy oleamide)	**1d**	Gln265Ala; Gln265Lys<br>Thr436Ala; Thr436Asn; Thr436Lys<br>Asn438Ala; Asn438Arg; Asn438Lys<br>His442Ala; His442Asn; His442Gln	Gln265Ala/Asn438Ala<br>Gln265Lys/Thr436Lys<br>Gln265Lys/Asn438Lys<br>Thr436Ala/Asn438Ala	Gln265Ala/Thr436Ala/Asn438Ala	
Alcohol	**1e**	Gln265Ala; Gln265Lys<br>Thr436Ala; Thr436Asn; Thr436Lys<br>Asn438Ala; Asn438Arg; Asn438Lys<br>His442Ala; His442Asn; His442Gln	Gln265Ala/Asn438Ala<br>Gln265Lys/Thr436Lys<br>Gln265Lys/Asn438Lys<br>Thr436Ala/Asn438Ala	Gln265Ala/Thr436Ala/Asn438Ala	

Table S2. (continued)

Head group	Entry	OhyA variants applied in conversions			
		Single variants	Double variants	Triple variants	Quadruple variants
Esters	**1f**	Gln265Ala; Gln265Glu; Gln265Lys; Gln265Ser<br>Thr436Ala<br>Asn438Ala<br>His442Ala	Gln265Ala/Asn438Ala<br>Thr436Ala/Asn438Ala	Gln265Ala/Thr436Ala/Asn438Ala	Gln265Ala/Thr436Ala/Asn438Ala/His442Ala
	**1g–1j**	Gln265Ala<br>Thr436Ala<br>Asn438Ala<br>His442Ala	Gln265Ala/Asn438Ala<br>Thr436Ala/Asn438Ala	Gln265Ala/Thr436Ala/Asn438Ala	Gln265Ala/Thr436Ala/Asn438Ala/His442Ala

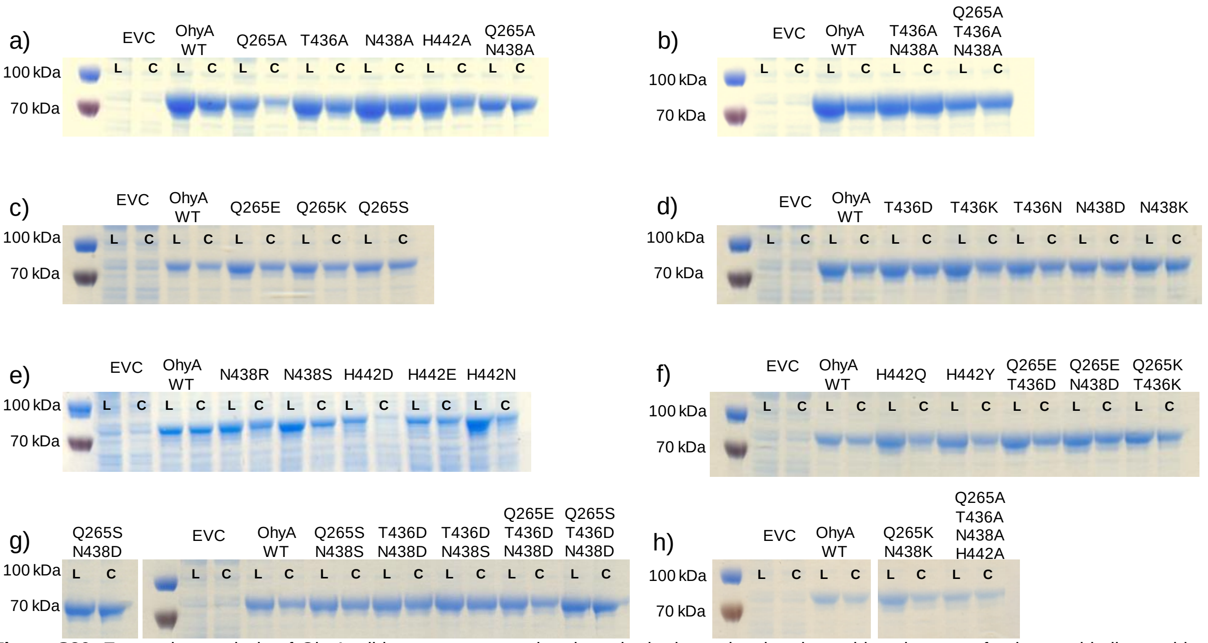

Figure S30. Expression analysis of OhyA wild type enzyme and variants harboring rational amino acid exchanges of substrate binding residues. The level of recombinant hydratase present in *E. coli* cell lysate before (lanes indicated with 'T' for total lysate) and after (lanes indicated with 'C' for cell-free lysate) separation of insoluble proteins was analyzed. Two µL of lysate was loaded in each lane. To allow for easier comparison of the protein amounts, cell lysate containing the wild type enzyme was loaded on each gel (a–h).

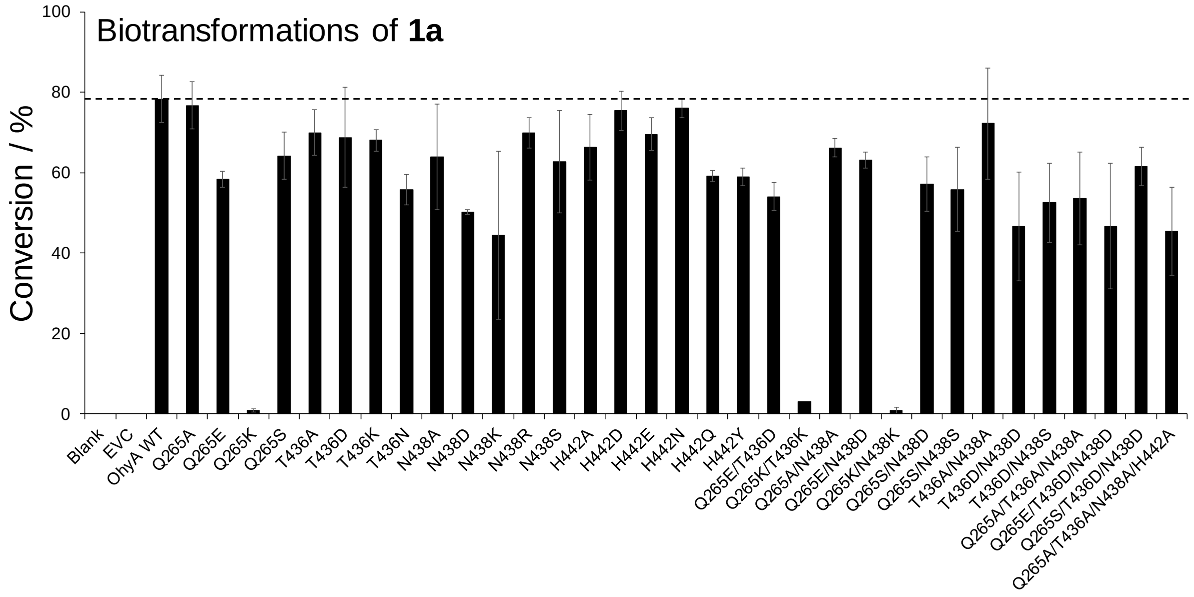

Figure S31. Conversion of **1a** by OhyA wild type and the amino acid exchange variants as whole cell *E. coli* biocatalysts after over-expression of the enzymes. Control reactions contained either the substrate added to the reaction buffer without cells or the substrate added to an *E. coli* empty vector control (EVC).

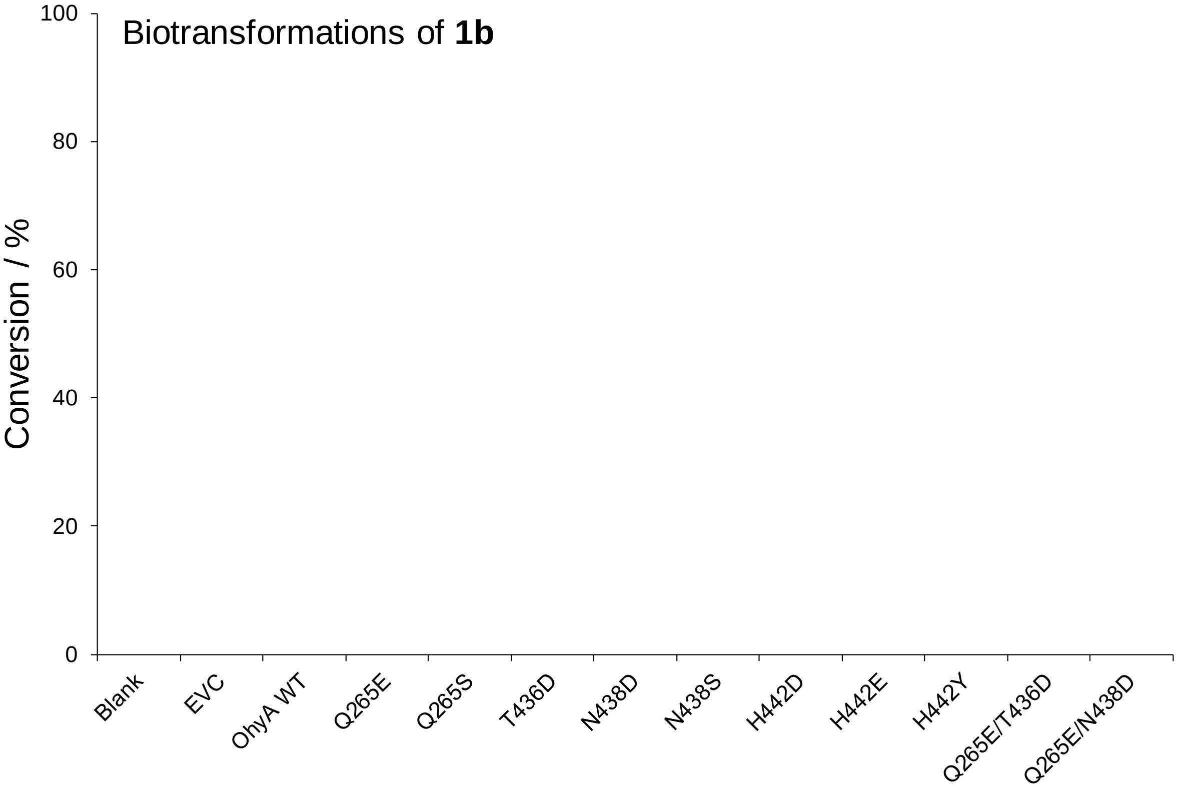

Figure S32. Conversion of **1b** by OhyA wild type and the amino acid exchange variants as whole cell *E. coli* biocatalysts after over-expression of the enzymes. Control reactions contained either the substrate added to the reaction buffer without cells or the substrate added to an *E. coli* empty vector control (EVC). No hydration of **1b** was obtained.

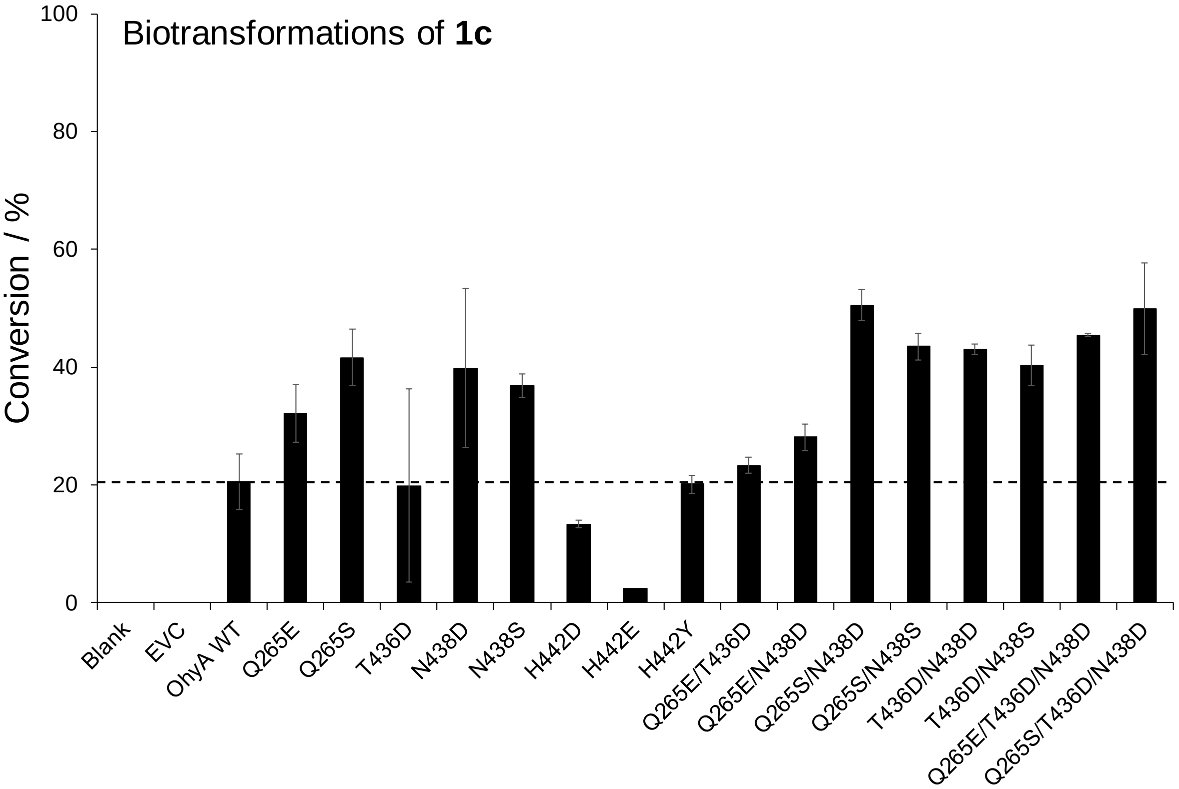

Figure S33. Conversion of **1c** by OhyA wild type and the amino acid exchange variants as whole cell *E. coli* biocatalysts after over-expression of the enzymes. Control reactions contained either the substrate added to the reaction buffer without cells or the substrate added to an *E. coli* empty vector control (EVC).

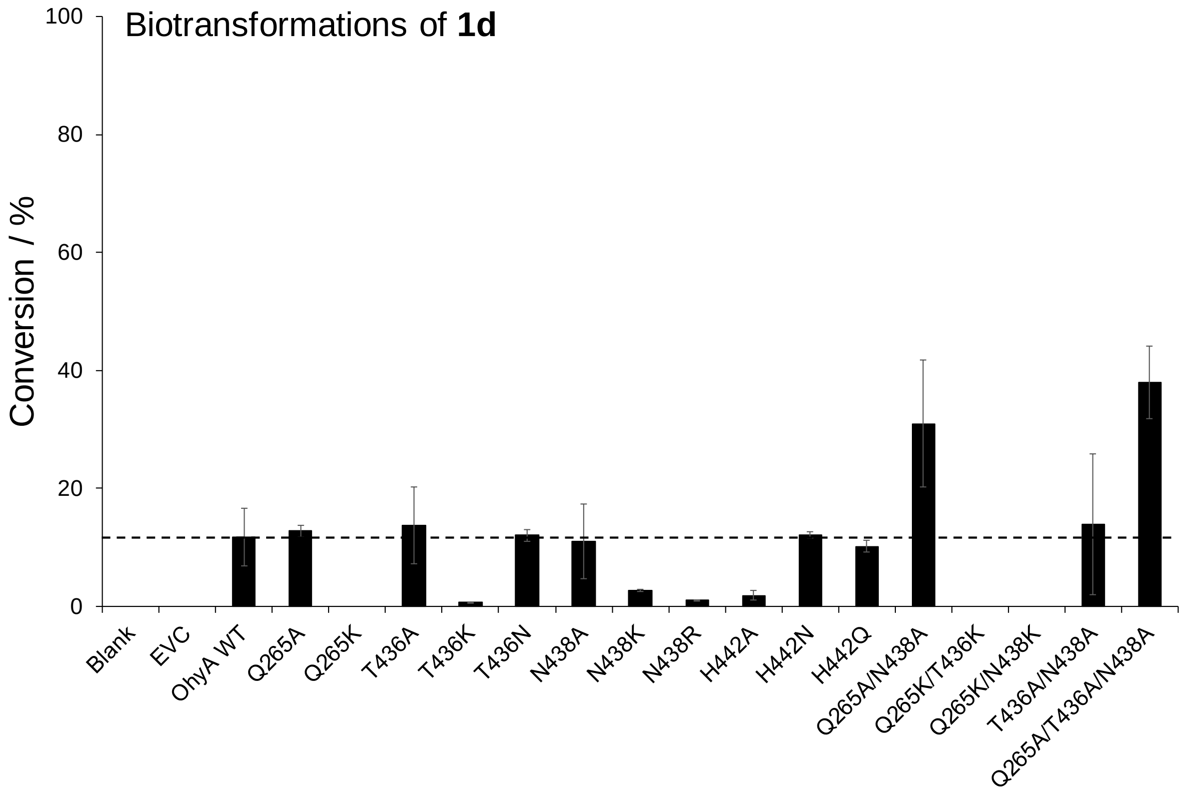

Figure S34. Conversion of **1d** by OhyA wild type and the amino acid exchange variants as whole cell *E. coli* biocatalysts after over-expression of the enzymes. Control reactions contained either the substrate added to the reaction buffer without cells or the substrate added to an *E. coli* empty vector control (EVC).

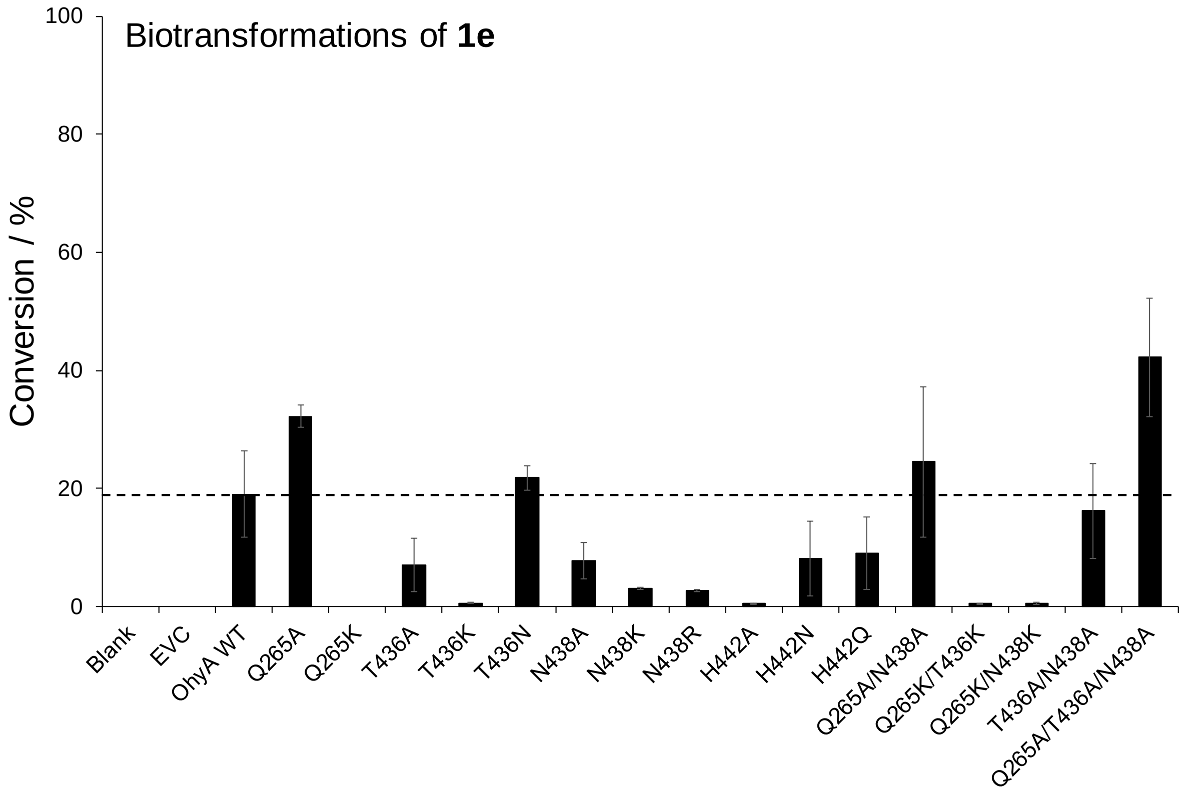

Figure S35. Conversion of **1e** by OhyA wild type and the amino acid exchange variants as whole cell *E. coli* biocatalysts after over-expression of the enzymes. Control reactions contained either the substrate added to the reaction buffer without cells or the substrate added to an *E. coli* empty vector control (EVC).

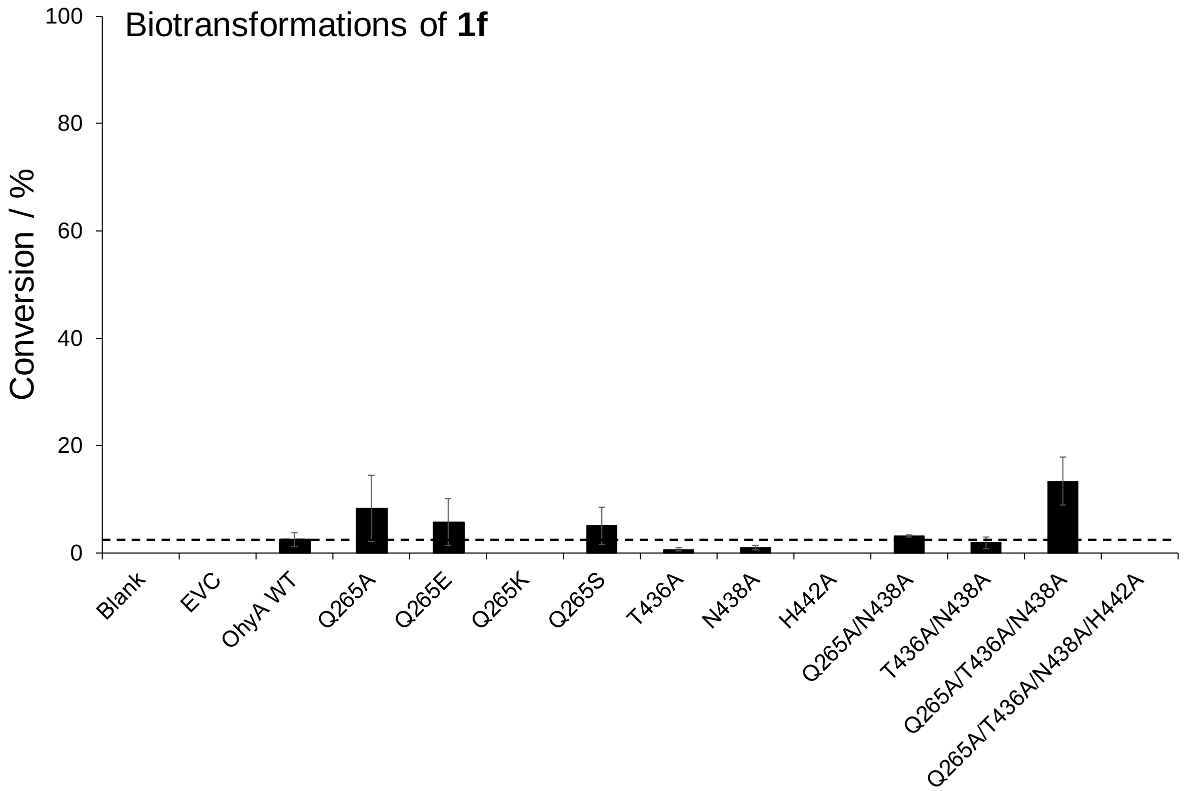

Figure S36. Conversion of **1f** by OhyA wild type and the amino acid exchange variants as whole cell *E. coli* biocatalysts after over-expression of the enzymes. Control reactions contained either the substrate added to the reaction buffer without cells or the substrate added to an *E. coli* empty vector control (EVC).

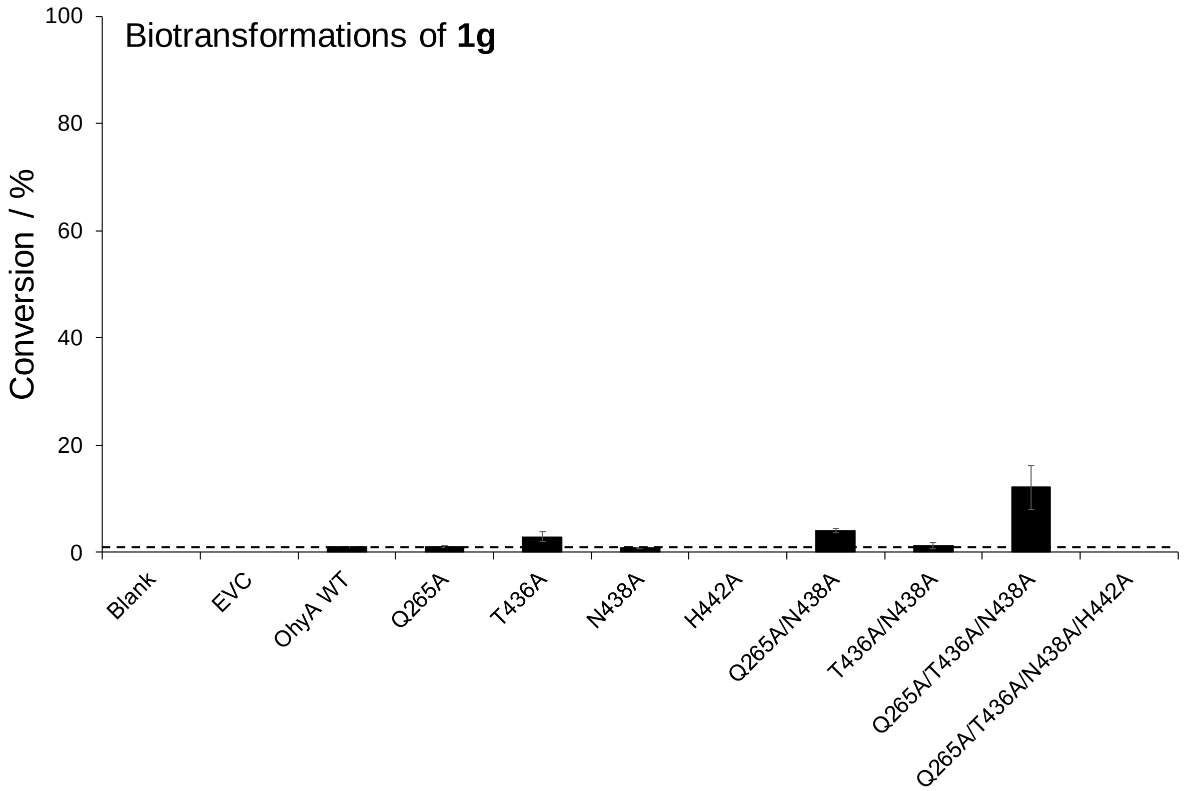

Figure S37. Conversion of **1g** by OhyA wild type and the amino acid exchange variants as whole cell *E. coli* biocatalysts after over-expression of the enzymes. Control reactions contained either the substrate added to the reaction buffer without cells or the substrate added to an *E. coli* empty vector control (EVC).

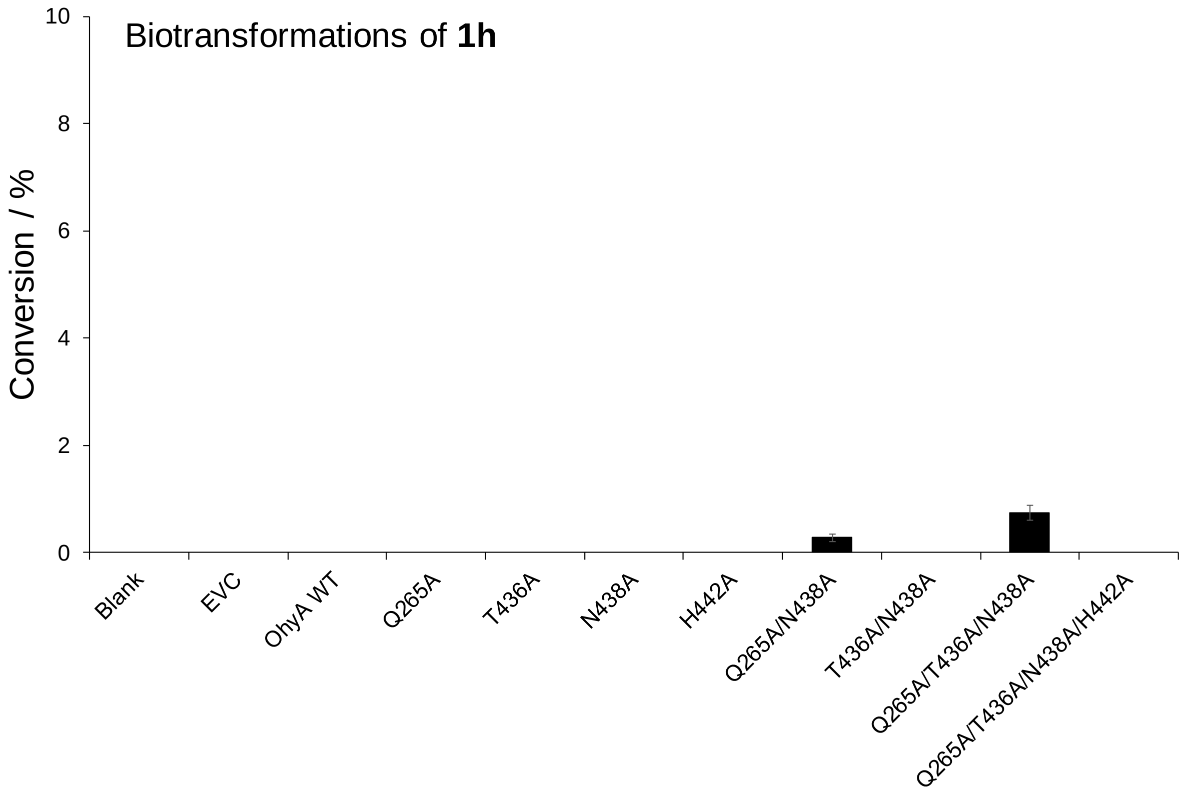

Figure S38. Conversion of **1h** by OhyA wild type and the amino acid exchange variants as whole cell *E. coli* biocatalysts after over-expression of the enzymes. Control reactions contained either the substrate added to the reaction buffer without cells or the substrate added to an *E. coli* empty vector control (EVC).

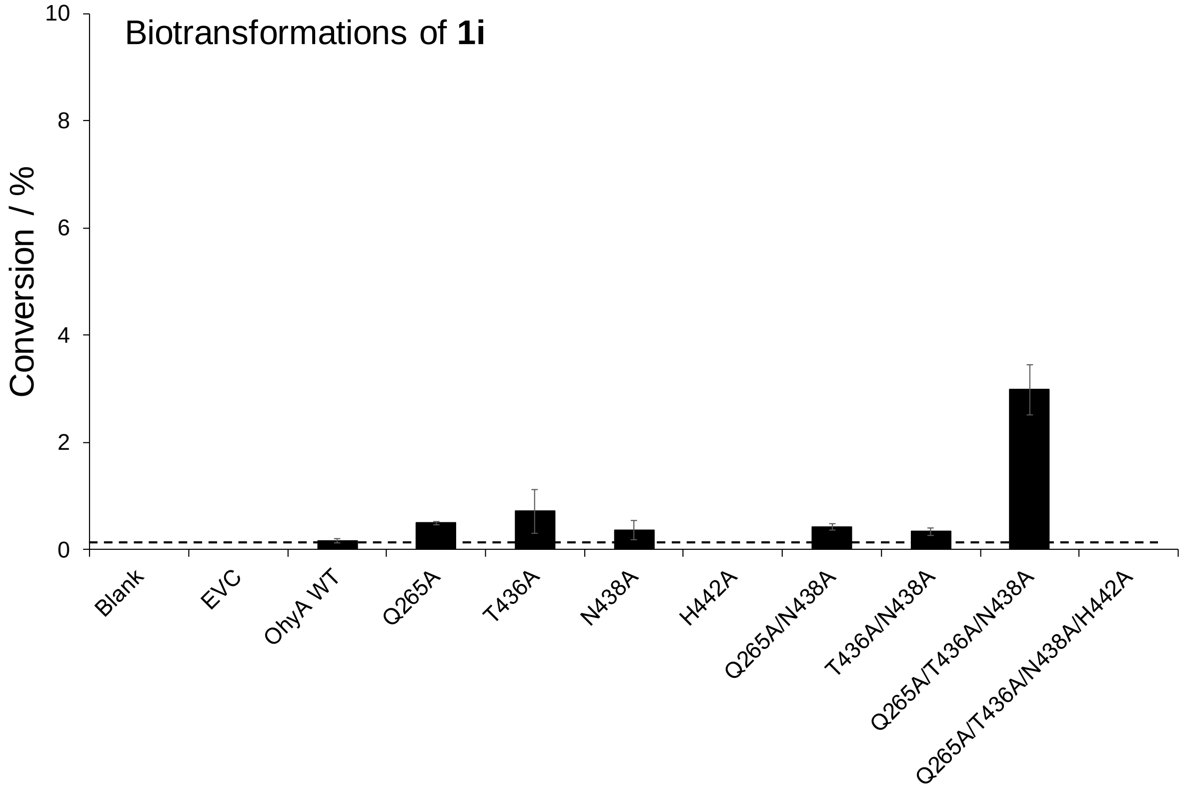

Figure S39. Conversion of **1i** by OhyA wild type and the amino acid exchange variants as whole cell *E. coli* biocatalysts after over-expression of the enzymes. Control reactions contained either the substrate added to the reaction buffer without cells or the substrate added to an *E. coli* empty vector control (EVC).

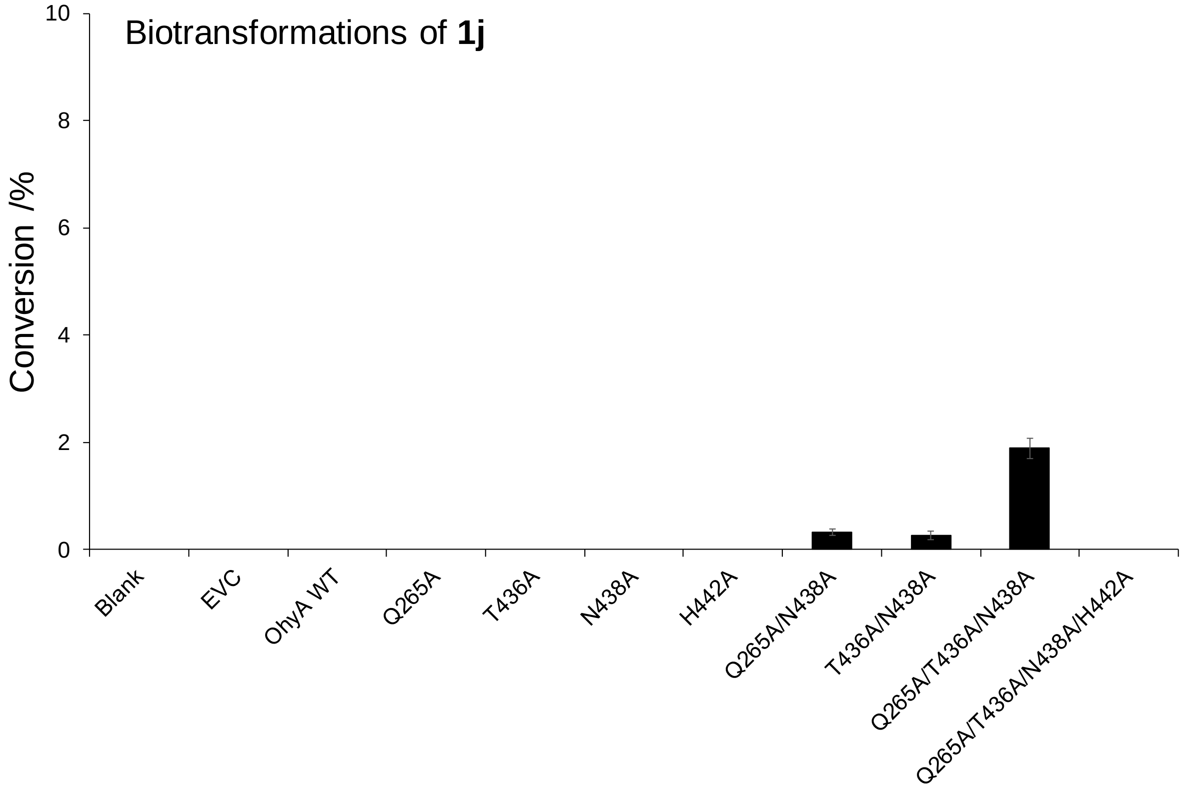

Figure S40. Conversion of **1j** by OhyA wild type and the amino acid exchange variants as whole cell *E. coli* biocatalysts after over-expression of the enzymes. Control reactions contained either the substrate added to the reaction buffer without cells or the substrate added to an *E. coli* empty vector control (EVC).

Stereoscopic imaging of modeling studies

a)

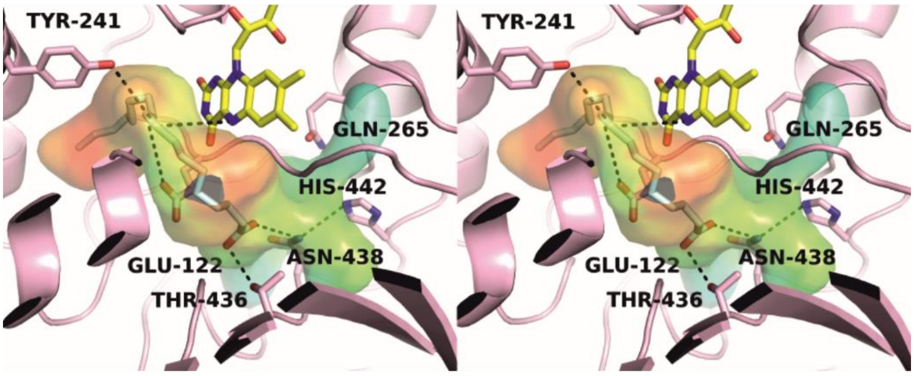

b)

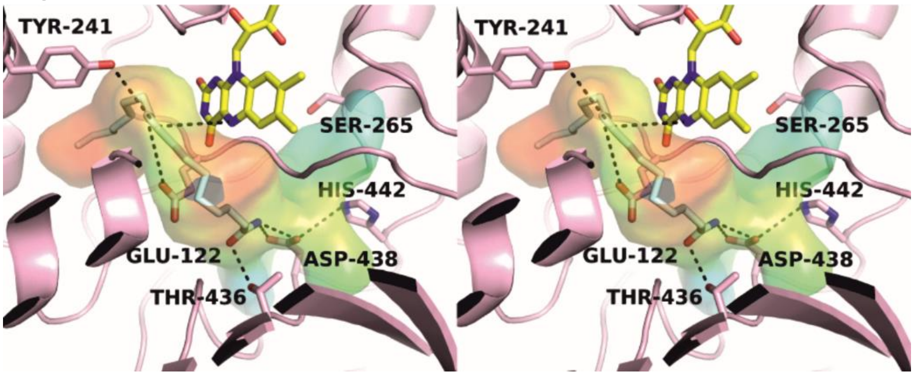

c)

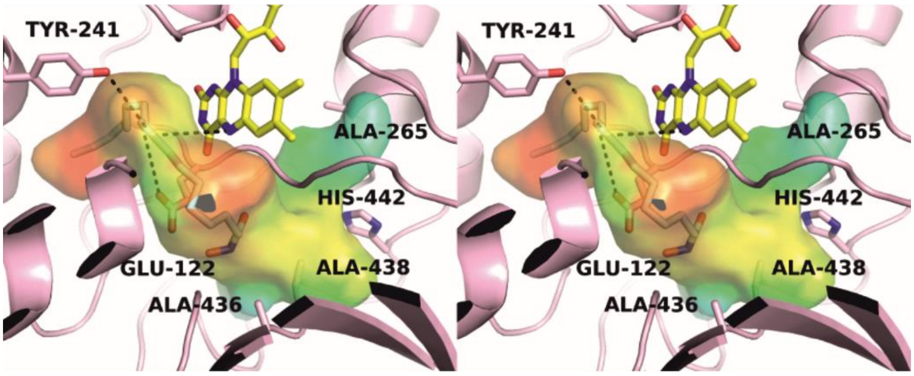

d)

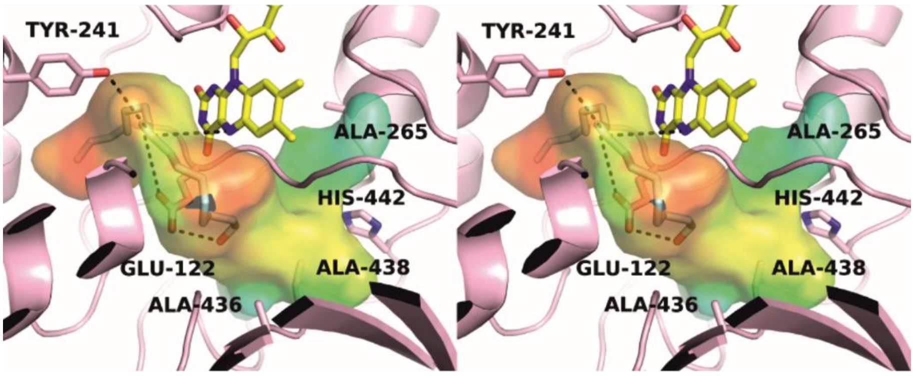

e)

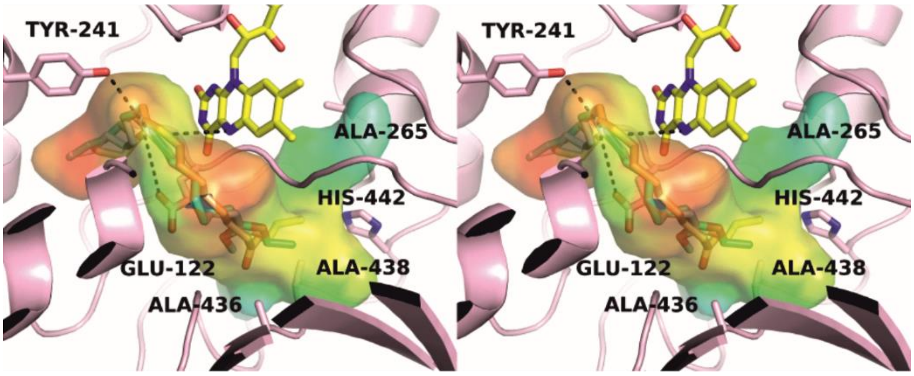

f)

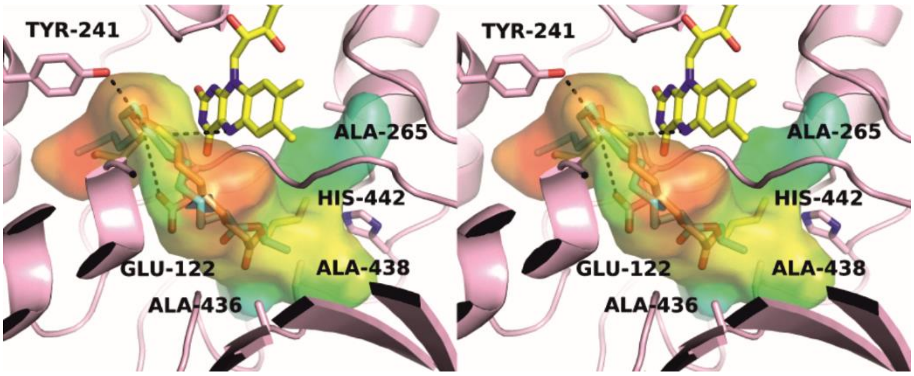

Figure S41. Stereoscopic representation of the in silico docking of oleic acid (**1a**) and oleic acid derivatives **1c**–**1j** to the OhyA 3D structure after mutagenesis of substrate binding residues (amino acid positions 265, 436, 438 and 442). The enzyme variant – substrate combinations resulting in the highest conversion are shown in the panels. The hydrophobicity of the enzyme cavity is represented by a color gradient from red (hydrophobic) to blue (hydrophilic). Co-crystallized FAD (yellow) and the substrates in the best docking mode are shown in stick representation. Substrate binding residues and catalytic Glu122 and Tyr241 are highlighted. a) Docking of (**1a**) to the 3D structure of OhyA wild type enzyme. b) Docking of oleamide (**1c**) to OhyA Q265S/N438D. c) Docking of *N*-hydroxy oleamide (**1d**) to OhyA Q265A/T436A/N438A. d) Docking of oleyl alcohol (**1e**) to OhyA Q265A/T436A/N438A. e) Docking of methyl (**1f**), ethyl (**1g**) and *n*-propyl (**1i**) oleate to OhyA Q265A/T436A/N438A. f) Docking of *i*-propyl (**1h**) and *n*-butyl (**1j**) oleate to OhyA Q265A/T436A/N438A.

Determination of the enantiomeric excess of reaction products by ^{1}H-NMR analysis

All products from the enzyme-catalyzed hydration reactions were *O*-acylated with (*S*)-(+)-*O*-acetylmandelic acid using a known procedure and purified by flash chromatography on silica gel using cyclohexane/ethyl acetate mixtures.[2] A reference material was obtained from methyl *rac*-10-hydroxy stearic acid for comparison in the ^{1}H-NMR analyses.[2]

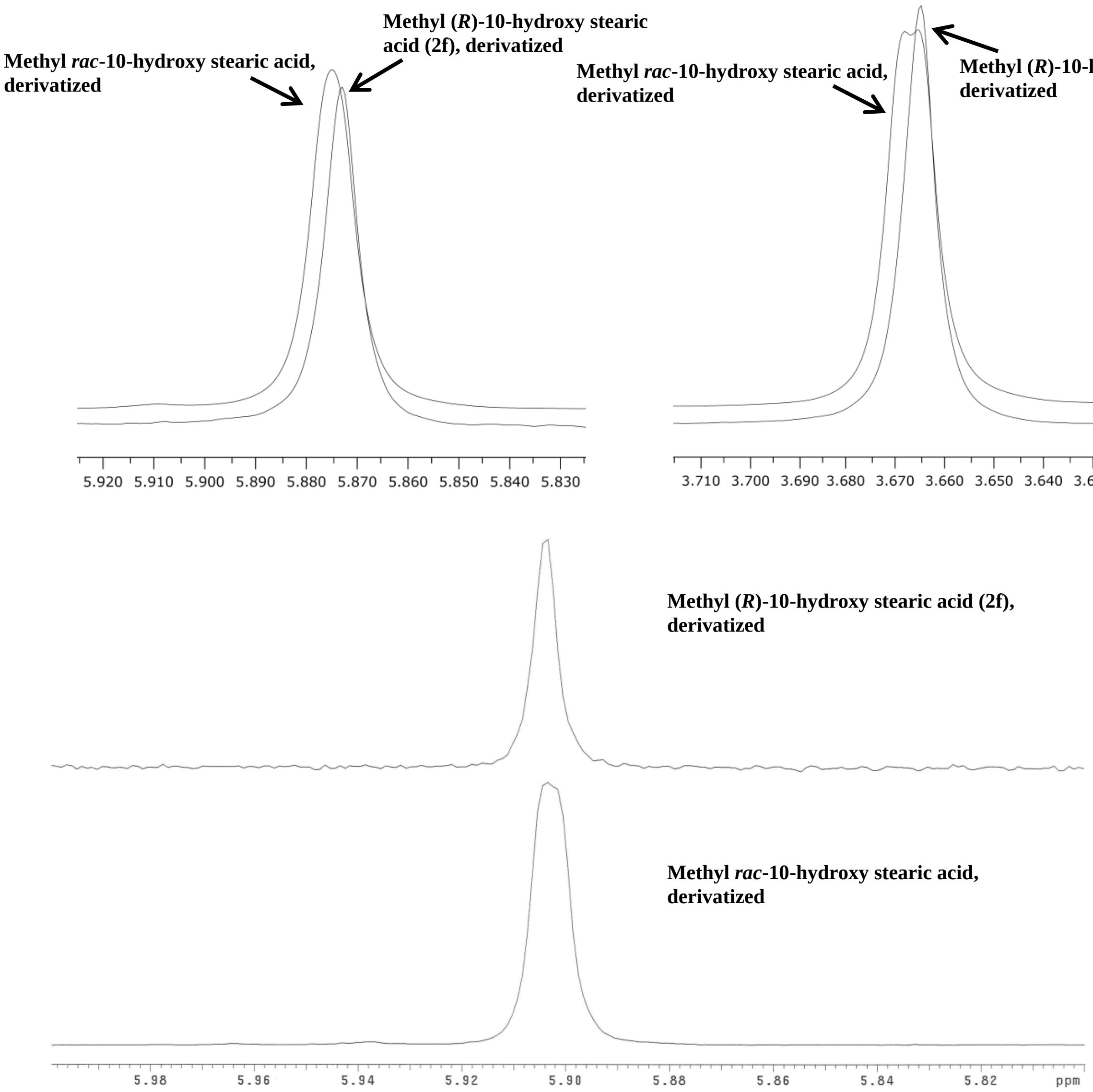

Figure S42. ^{1}H-NMR analysis ($CDCl_3$, 500 MHz) of (*S*)-*O*-acetylmandelic acid derivatized **2f** ((10*R*,2'*S*)- vs. (10*RS*,2'*S*)-diastereomers). The material derived from racemic methyl 10-hydroxy stearic acid shows a broader signal at 5.88 ppm as consequence of incomplete resolution of the 2'-H protons and a partly resolved methyl ester signal at 3.67 ppm. At both sections of the spectrum, the signals derived from derivatized methyl (*R*)-10-hydroxy stearic acid (**1a** converted to the corresponding methyl ester **1f**) appear sharper and are on the side of lower chemical shifts.

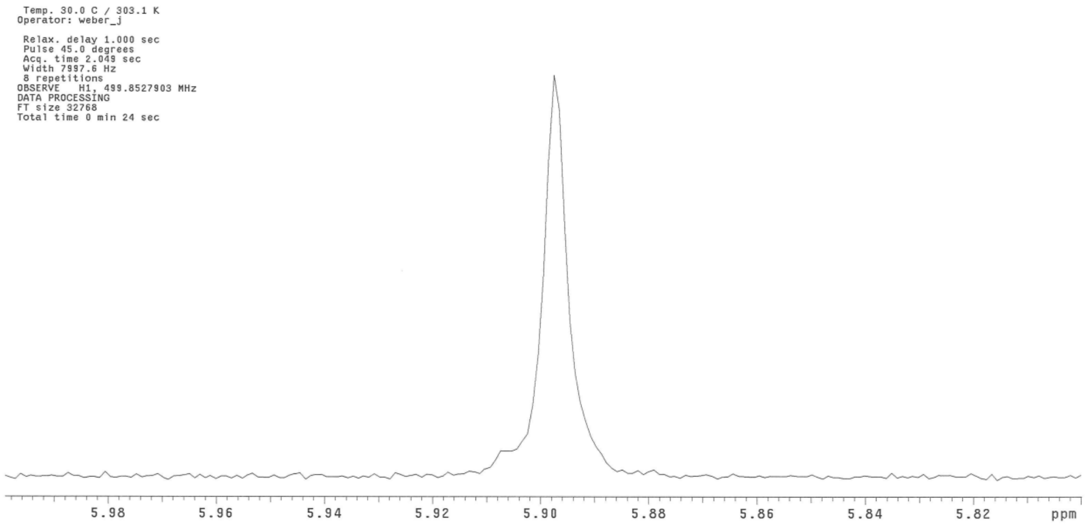

Figure S43. ^{1}H-NMR analysis ($CDCl_3$, 500 MHz) of (*S*)-*O*-acetylmandelic acid derivatized **2c**.

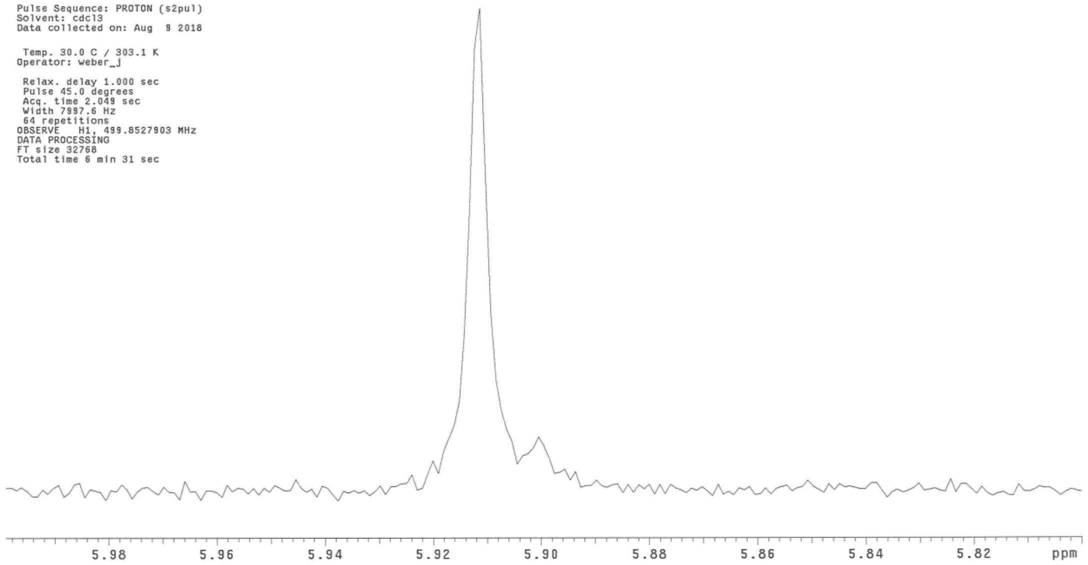

Figure S44. ^{1}H-NMR analysis ($CDCl_3$, 500 MHz) of (*S*)-*O*-acetylmandelic acid derivatized **2d**.

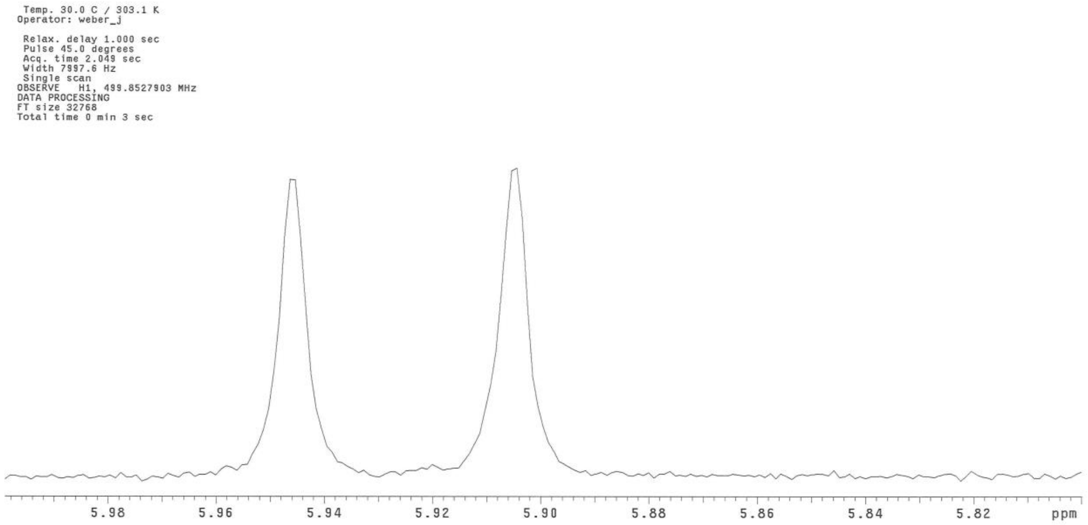

Figure S45. ^{1}H-NMR analysis ($CDCl_3$, 500 MHz) of (*S*)-*O*-acetylmandelic acid derivatized **2e** (both alcohol functionalities converted).

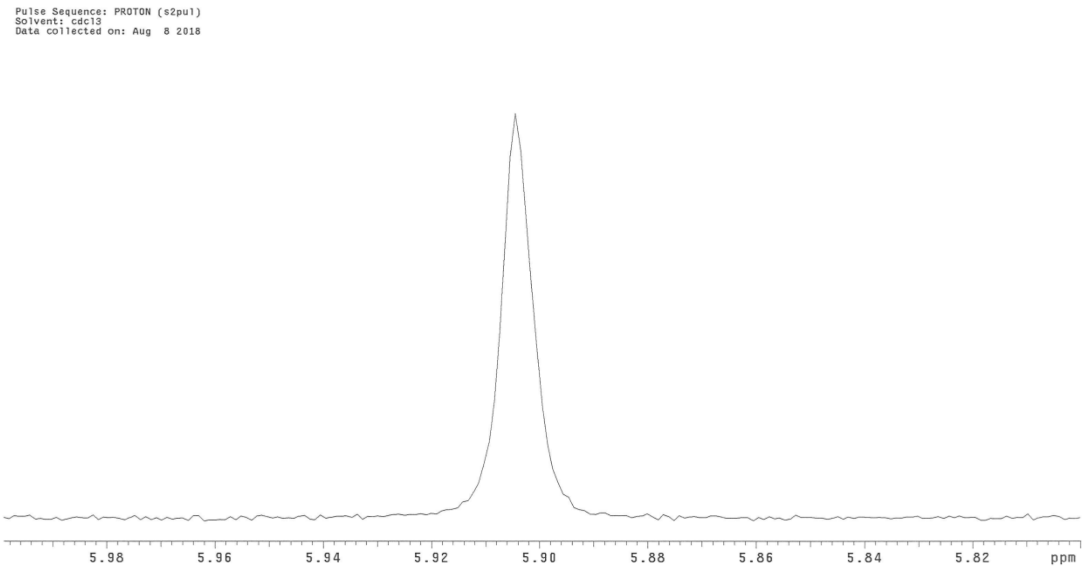

Figure S46. ^{1}H-NMR analysis ($CDCl_3$, 500 MHz) of (*S*)-*O*-acetylmandelic acid derivatized compound **2g**.

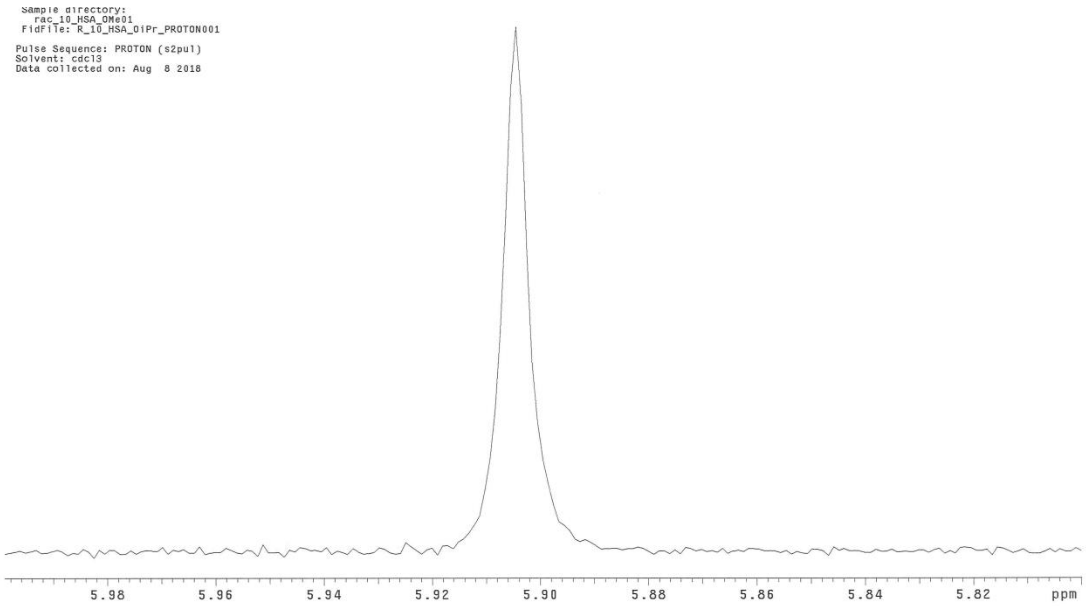

Figure S47. ^{1}H-NMR analysis ($CDCl_3$, 500 MHz) of (*S*)-*O*-acetylmandelic acid derivatized compound **2h**.

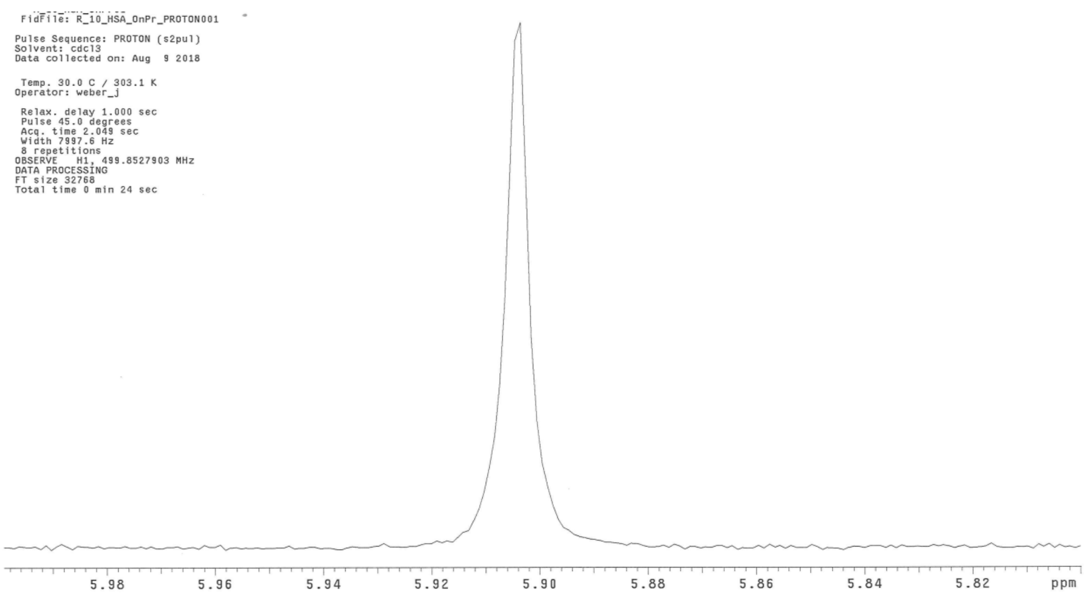

Figure S48. ^{1}H-NMR analysis ($CDCl_3$, 500 MHz) of (*S*)-*O*-acetylmandelic acid derivatized compound **2i**.

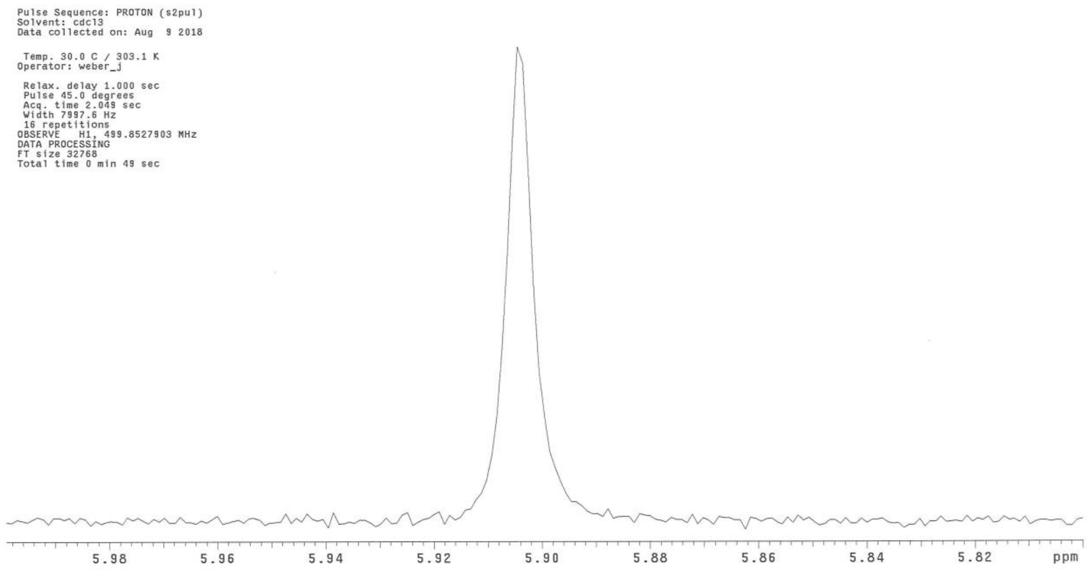

Figure S49. ^{1}H-NMR analysis ($CDCl_3$, 500 MHz) of (*S*)-*O*-acetylmandelic acid derivatized compound **2j**.

Based on the clear NMR proof of (*R*)-selectivity in case of the hydrated compounds **2a** and **2f**, the strict enzymatic reaction mechanism involved and the fact that all other compounds **2c**–**2e** and **2g**–**2j** show a similarly shaped sharp NMR signal at 5.90 ppm, we conclude that the reaction proceeds in all cases with high stereoselectivity (*ee* ≥ 95 %) and that the products are the expected (*R*)-10-alcohols.

Determination of OhyA wild type and variant conversions by GC-FID

Hydration reactions of **1a**–**1j** were quantified via GC on a Shimadzu GC-2010 Plus instrument equipped with a flame ionization detector and a Phenomenex Zebron ZB-5 column under the conditions described in the Experimental Procedures section of the Supporting Information. The improvement in catalytic activity is illustrated by representative chromatograms from wild type and the best variant conversions of each OA derivative (Figure S49–S56). Integration results are shown as peak area values for each substrate and product, respectively.

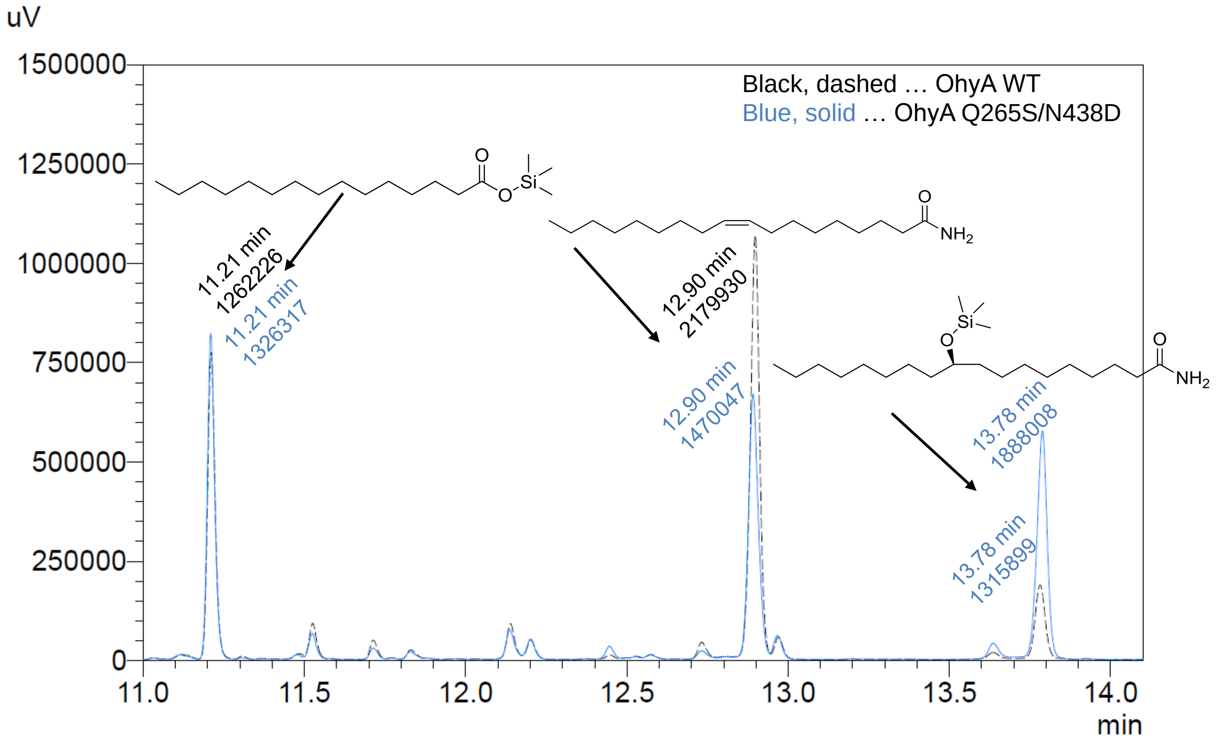

Figure S50. Overlay of GC-FID chromatograms from bioconversions of oleamide (**1c**) to **2c** with *E. coli* whole cells over-expressing OhyA wild type and OhyA Gln265Ser/Asn438Asp. Retention times and peak area values for the TMS-derivative of the internal standard *n*-pentadecanoic acid (11.21 min), **1c** (12.90 min) and **2c** (13.78 min) are highlighted.

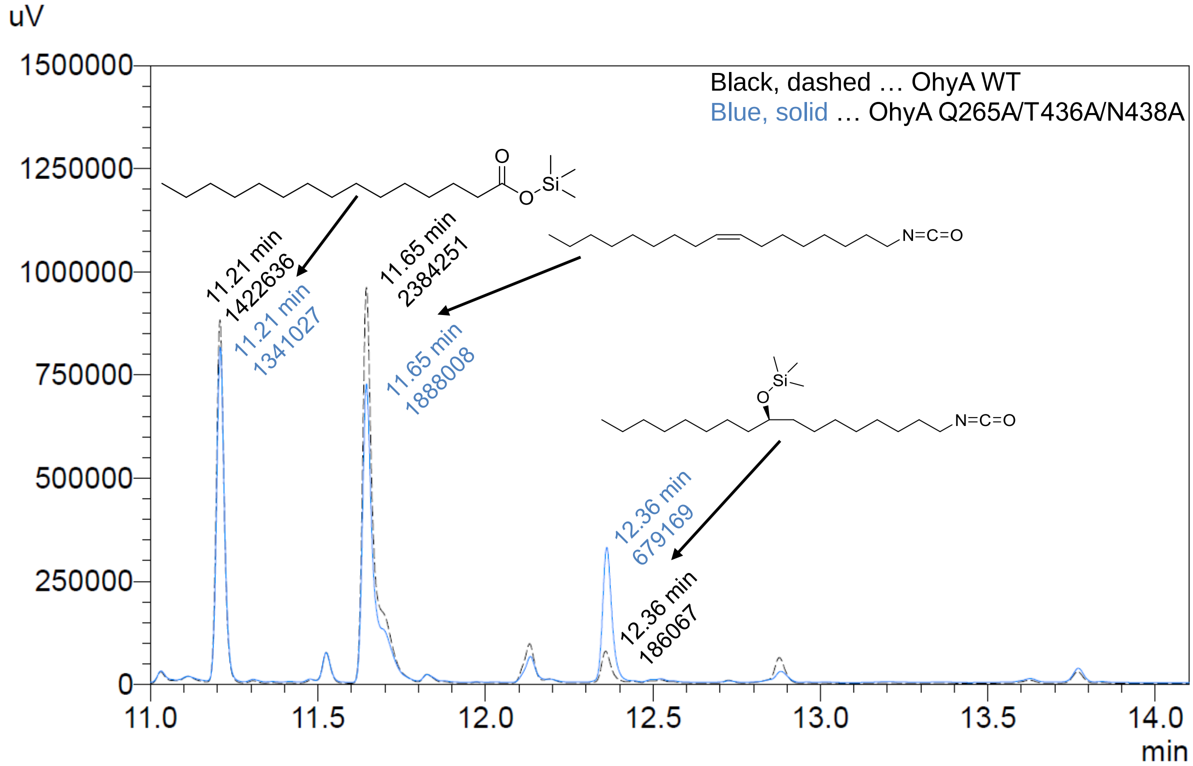

Figure S51. Overlay of GC-FID chromatograms from bioconversions of *N*-hydroxy oleamide (**1d**) to **2d** with *E. coli* whole cells over-expressing OhyA wild type and OhyA Gln265Ala/Thr436Ala/Asn438Ala. Retention times and peak area values for the TMS-derivative of the internal standard *n*-pentadecanoic acid (11.21 min), **1d** (11.65 min) and **2d** (12.36 min) are highlighted.

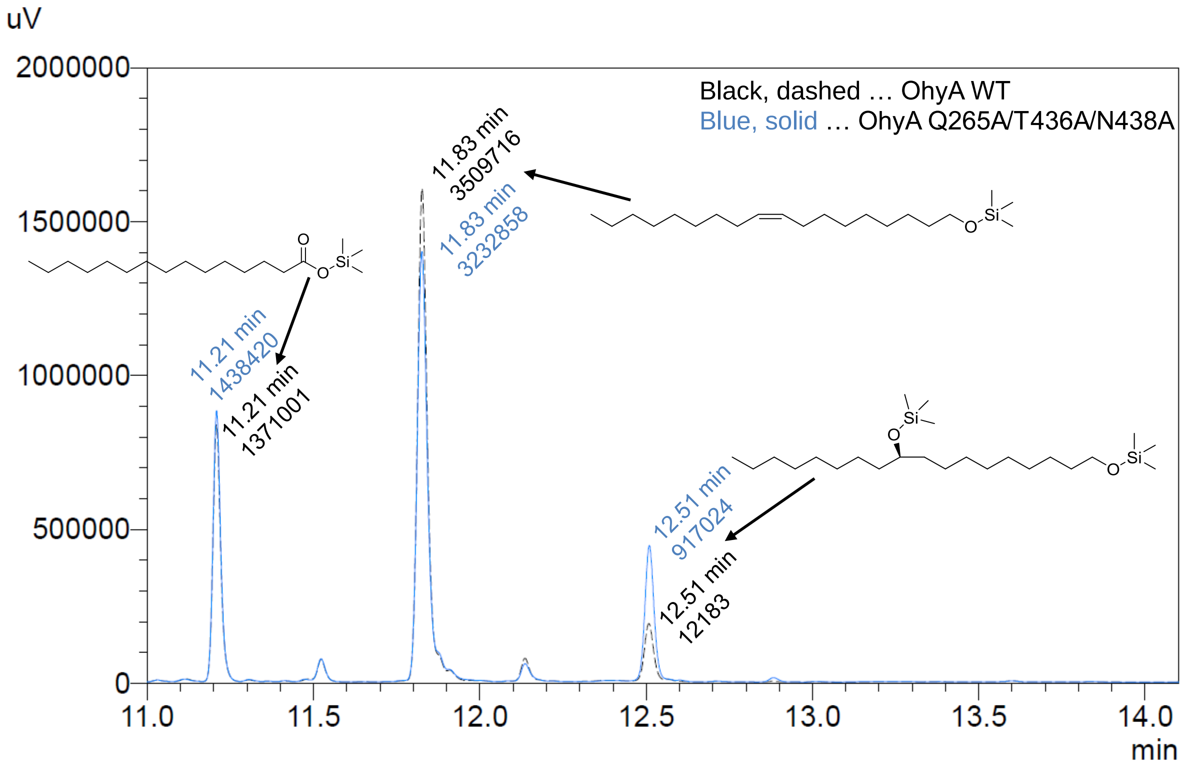

Figure S52. Overlay of GC-FID chromatograms from bioconversions of oleyl alcohol (**1e**) to **2e** with *E. coli* whole cells over-expressing OhyA wild type and OhyA Gln265Ala/Thr436Ala/Asn438Ala. Retention times and peak area values for the TMS-derivative of the internal standard *n*-pentadecanoic acid (11.21 min), **1e** (11.83 min) and **2e** (12.51 min) are highlighted.

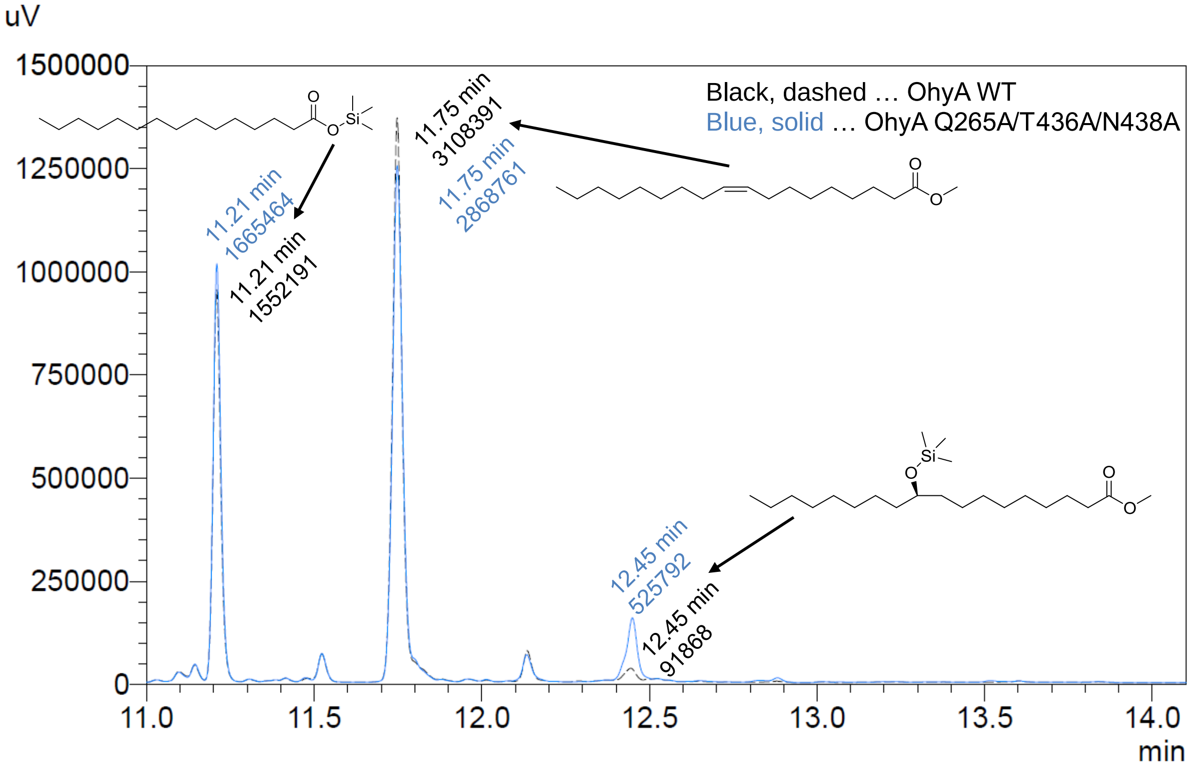

Figure S53. Overlay of GC-FID chromatograms from bioconversions of methyl oleate (**1f**) to **2f** with *E. coli* whole cells over-expressing OhyA wild type and OhyA Gln265Ala/Thr436Ala/Asn438Ala. Retention times and peak area values for the TMS-derivative of the internal standard *n*-pentadecanoic acid (11.21 min), **1f** (11.75 min) and **2f** (12.45 min) are highlighted.

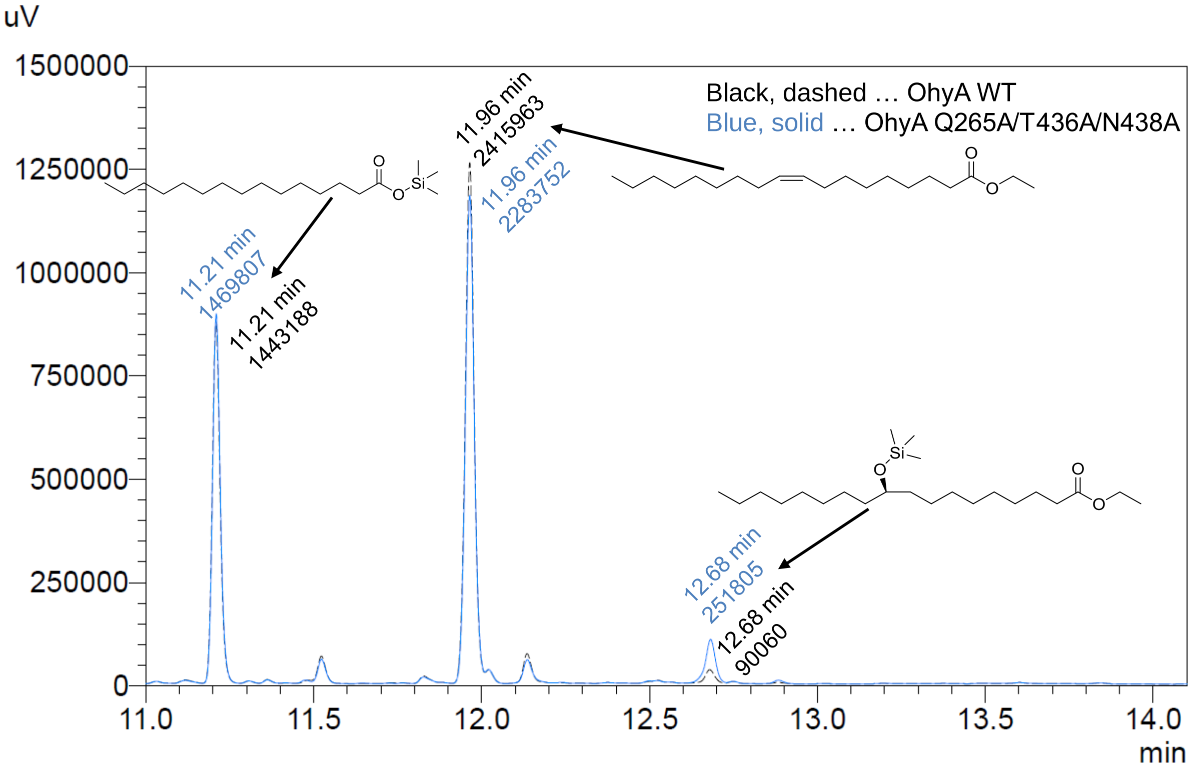

Figure S54. Overlay of GC-FID chromatograms from bioconversions of ethyl oleate (**1g**) to **2g** with *E. coli* whole cells over-expressing OhyA wild type and OhyA Gln265Ala/Thr436Ala/Asn438Ala. Retention times and peak area values for the TMS-derivative of the internal standard *n*-pentadecanoic acid (11.21 min), **1g** (11.96 min) and **2g** (12.68 min) are highlighted.

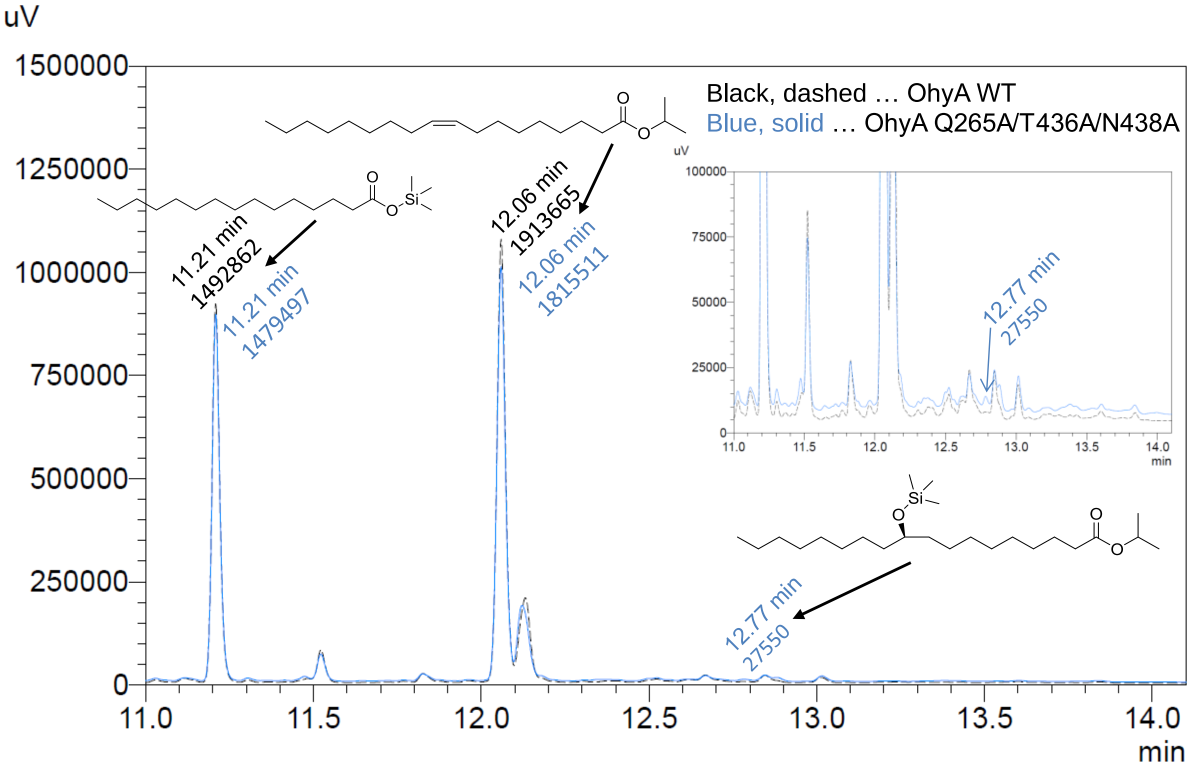

Figure S55. Overlay of GC-FID chromatograms from bioconversions of *i*-propyl oleate (**1h**) to **2h** with *E. coli* whole cells over-expressing OhyA wild type and OhyA Gln265Ala/Thr436Ala/Asn438Ala. Retention times and peak area values for the TMS-derivative of the internal standard *n*-pentadecanoic acid (11.21 min), **1h** (12.06 min) and **2h** (12.77 min) are highlighted. The insert shows a zoom-in to the section in which the reaction product **2h** is eluting.

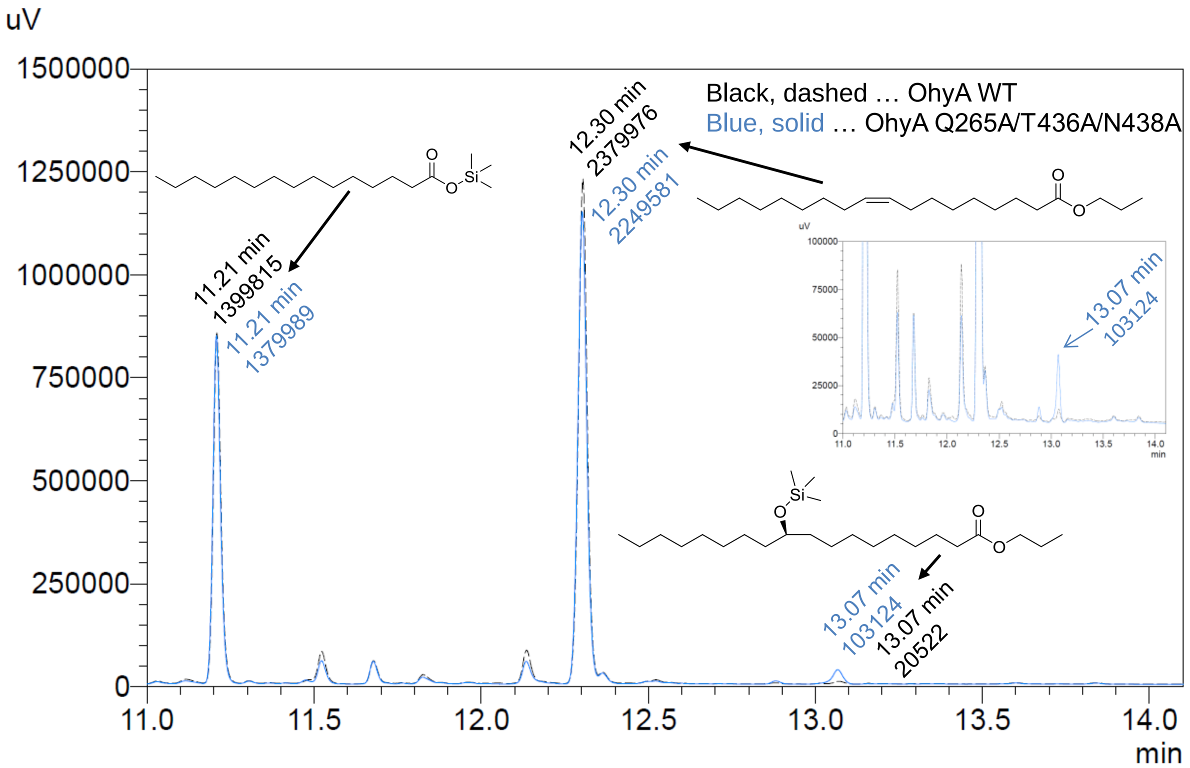

Figure S56. Overlay of GC-FID chromatograms from bioconversions of *n*-propyl oleate (**1i**) to **2i** with *E. coli* whole cells over-expressing OhyA wild type and OhyA Gln265Ala/Thr436Ala/Asn438Ala. Retention times and peak area values for the TMS-derivative of the internal standard *n*-pentadecanoic acid (11.21 min), **1i** (12.30 min) and **2i** (13.07 min) are highlighted. The insert shows a zoom-in to the section in which the reaction product **2i** is eluting

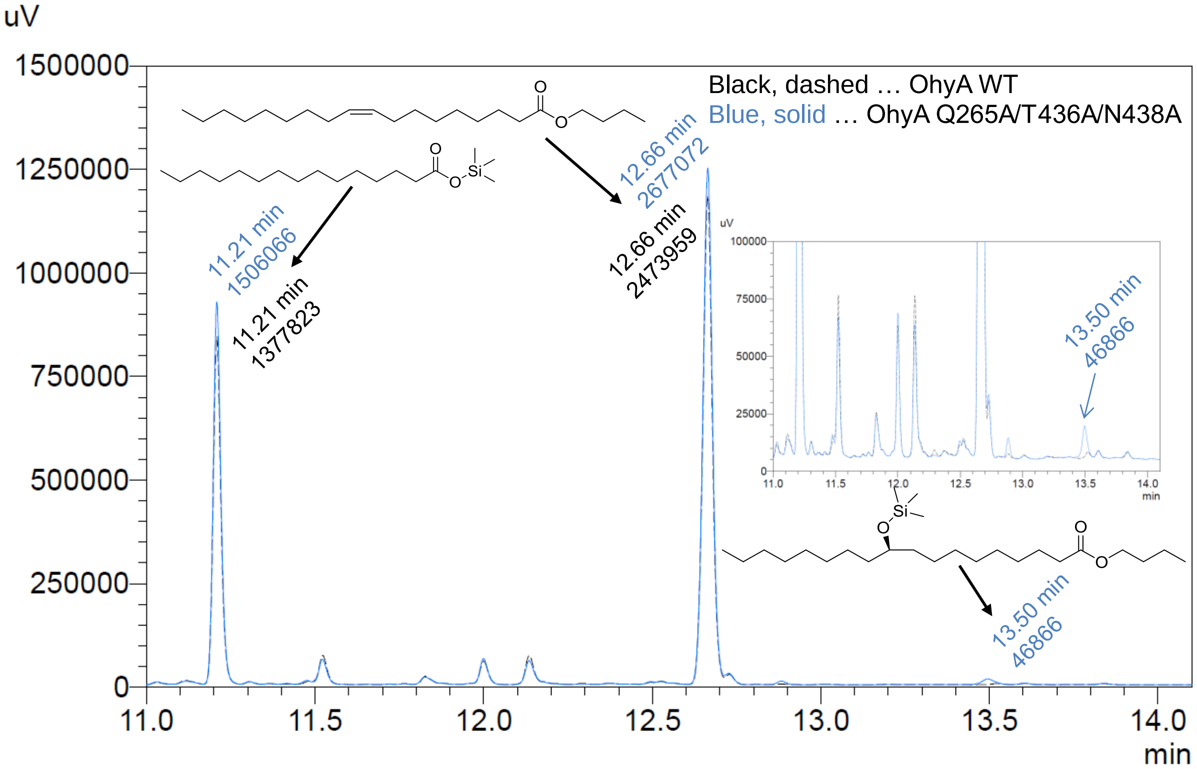

Figure S57. Overlay of GC-FID chromatograms from bioconversions of *n*-butyl oleate (**1j**) to **2j** with *E. coli* whole cells over-expressing OhyA wild type and OhyA Gln265Ala/Thr436Ala/Asn438Ala. Retention times and peak area values for the TMS-derivative of the internal standard *n*-pentadecanoic acid (11.21 min), **1j** (12.66 min) and **2j** (13.50 min) are highlighted. The insert shows a zoom-in to section in which the reaction product **2j** is eluting.

Control reactions with OA esters

In vivo hydrolysis of fatty acid esters is inherent to essentially all microbes, including *E. coli*.[12] To exclude any side reactions, in particular hydration of the corresponding free fatty acids after ester cleavage and re-esterification with available alcohols, respectively, we co-incubated **1f** (Figure S57) and **1g** (Figure S58) with methanol, ethanol or *i*-propanol. For experimental details,

please refer to the section ‘Whole cell bioconversions’ in the Experimental Procedures part of the Supporting Information. For this control experiment, we applied only the OhyA triple variant Gln265Ala/Thr436Ala/Asn438Ala, since this enzyme variant showed the highest activity towards **1f** and **1g**. Owing to the non-formation of 10-hydroxy stearic acid acid ethyl and *i*-propyl esters in biotransformations of **1f**, as well as the absence of any 10-hydroxy stearic acid acid methyl and *i*-propyl esters in biotransformations of **1g**, generation of free acid-derived hydration products was highly unlikely.

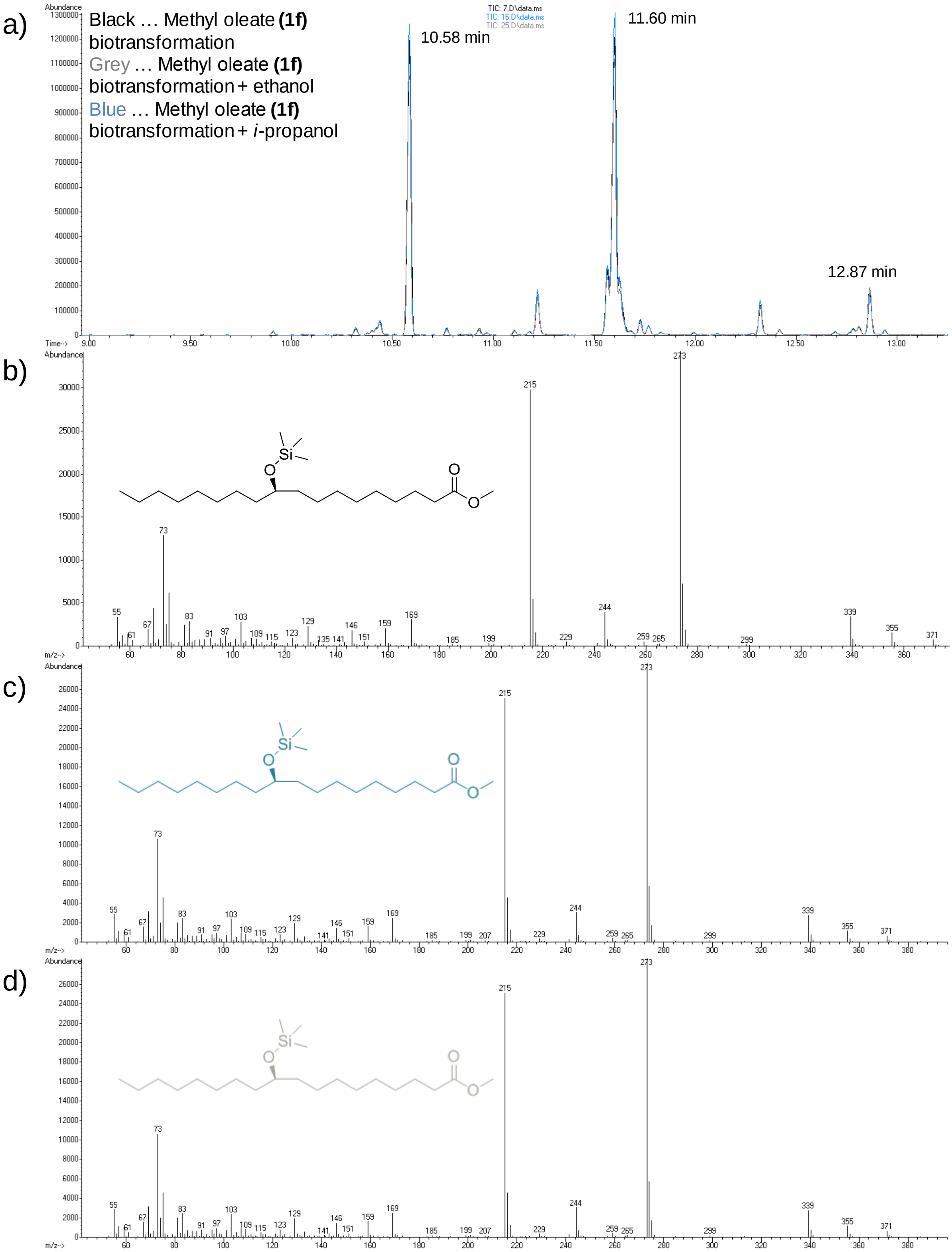

Figure S58. Bioconversion of methyl oleate (**1f**) to **2f** with *E. coli* whole cells over-expressing OhyA Gln265Ala/Thr436Ala/Asn438Ala. Assays were co-incubated with alcohol additives ethanol or *i*-propanol for monitoring of potential side reactions from hydration of **1f** after its possible hydrolysis to **1a** by *E. coli*-endogenous hydrolases and subsequent re-esterification with available alcohols. a) Overlay of representative GC-MS chromatograms from technical triplicates of **1f** biotransformations without alcohol additives and with addition of either ethanol or *i*-propanol. Retention times of the TMS-derivative of the internal standard *n*-pentadecanoic acid (10.58 min), **1f** (11.60 min) and the TMS-derivative of **2f** (12.87 min) are highlighted. b) Mass spectrum of **2f** in biotransformations without alcohol additives. c) Mass spectrum of **2f** in biotransformations with ethanol as alcohol additive. d) Mass spectrum of **2f** in biotransformations with *i*-propanol as alcohol additive. Based on the absence of additional peaks and the identical mass spectra in b) – d), we can exclude any of the aforementioned side reactions of **1f** in whole *E. coli* cells.

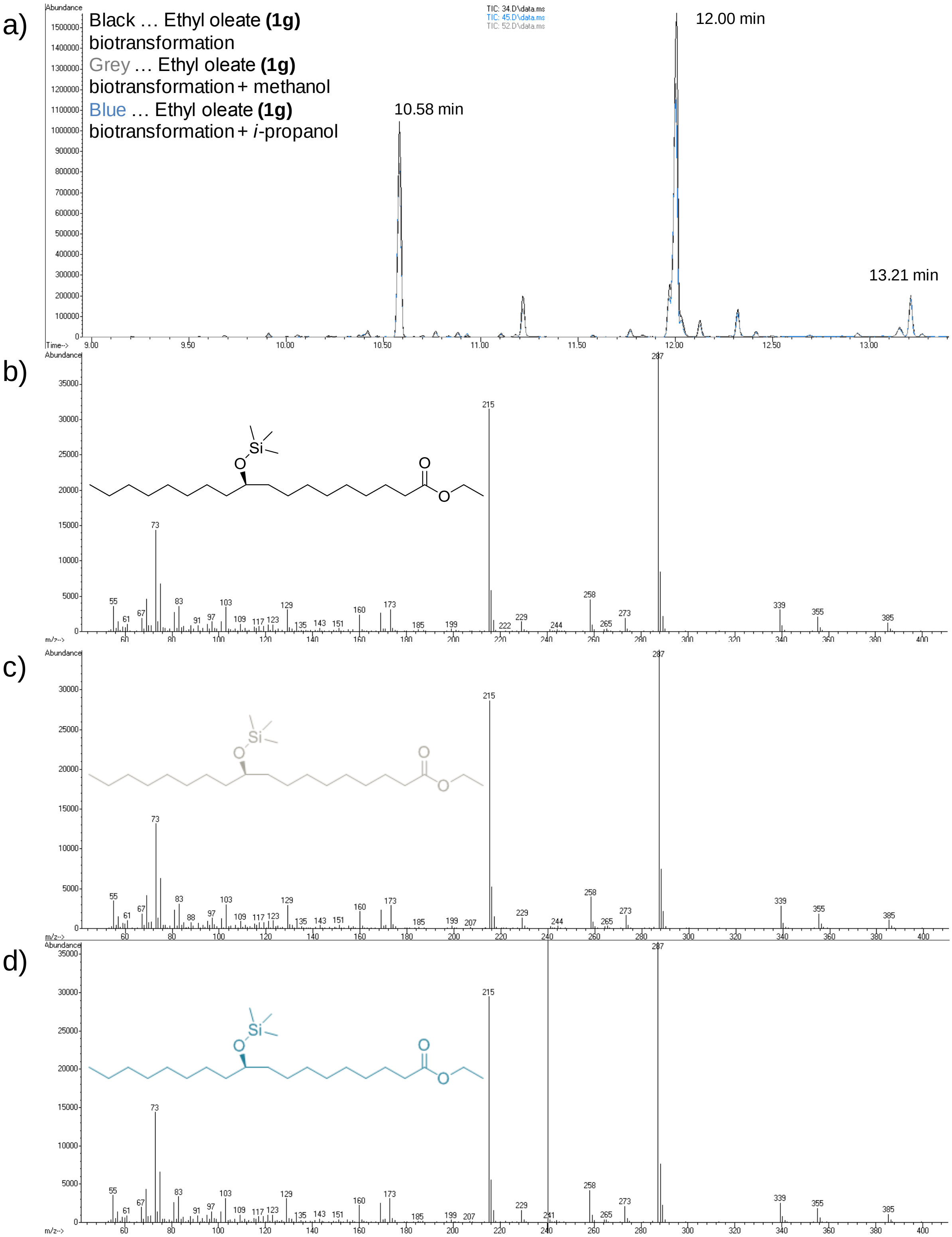

Figure S59. Bioconversion of ethyl oleate (**1g**) to **2g** with *E. coli* whole cells over-expressing OhyA Gln265Ala/Thr436Ala/Asn438Ala. Assays were co-incubated with alcohol additives methanol or *i*-propanol for monitoring of potential side reactions from hydration of **1g** after its possible hydrolysis by *E. coli*-endogenous hydrolases and subsequent re-esterification with available alcohols. a) Overlay of representative GC-MS chromatograms from technical triplicates of **1g** biotransformations without alcohol additives and with addition of either methanol or *i*-propanol. Retention times of the TMS-derivative of the internal standard *n*-pentadecanoic acid (10.58 min), **1g** (12.00 min) and the TMS-derivative of **2g** (13.21 min) are highlighted. b) Mass spectrum of **2g** at 13.21 min in biotransformations without alcohol additives. c) Mass spectrum of **2g** in biotransformations with methanol as alcohol additive. d) Mass spectrum of **2g** in biotransformations with *i*-propanol as alcohol additive. Based on the absence of additional peaks and the identical mass spectra in b) – d), we can exclude any of the aforementioned side reactions of **1g** in whole *E. coli* cells.

References

[1] F. M. Ausubel, R. Brent, R. E. Kingston, D. D. Moore, J. G. Seidman, J. A. Smith, K. Struhl, *Current Protocols in Molecular Biology*, Wiley-VCH, Weinheim, **2003**.

[2] M. Engleder, T. Pavkov-Keller, A. Emmerstorfer-Augustin, A. Hromic, S. Schrempf, G. Steinkellner, T. Wriessnegger, E. Leitner, G. A. Strohmeier, I. Kaluzna, D. Mink, M. Schürmann, S. Wallner, P. Macheroux, K. Gruber, H. Pichler, *ChemBioChem* **2015**, *16*, 1730-1734.
[3] J. Schmid, L. Steiner, S. Fademrecht, J. Pleiss, K. B. Otte, B. Hauer, *J. Mol. Catal. B: Enzym.* **2016**, *133*, 243–249.
[4] F. Sievers, A. Wilm, D. Dineen, T. J. Gibson, K. Karplus, W. Li, R. Lopez, H. McWilliam, M. Remmert, J. Söding, J. D. Thompson, D. G. Higgins, *Mol. Syst. Biol.* **2011**, *7*, 539.
[5] C. Fong, D. Wells, I. Krodkiewska, P. G. Hartley, C. J. Drummond, *Chem. Mater.* **2006**, *18*, 594–597.
[6] H. P. Kaufmann, K.-J. Skiba, *Fette, Seifen, Anstrichm.* **1958**, *60*, 362–364.
[7] B. R. Moser, B. K. Sharma, K. M. Doll, S. Z. Erhan, *J. Am. Oil Chem. Soc.* **2007**, *84*, 675–680.
[8] O. Trott, A. J. Olson, *J. Comput. Chem.* **2010**, *31*, 455–461.
[9] O. Kreye, S. Wald, M. A. R. Meier, *Adv. Synth. Catal.* **2013**, *355*, 81–86.
[10] K. Sugihara, S. Kitamura, S. Ohta, K. Tatsumi, *Xenobiotica* **2000**, *30*, 457–467.
[11] A. Brunner, L. Hintermann, *Helv. Chim. Acta* **2016**, *99*, 928–943.
[12] M. Kadisch, A. Schmid, B. Buehler, *J. Ind. Microbiol. Biotechnol.* **2017**, *44*, 339–351.

Author contributions

M. Engleder: Conceptualization, data curation, formal analysis, investigation, methodology, validation, visualization, writing - original draft, writing - review & editing
G. A. Strohmeier: Conceptualization, data curation, formal analysis, investigation, methodology, validation, visualization, writing - review & editing
H. Weber: Conceptualization, formal analysis, investigation, methodology, validation, visualization
G. Steinkellner: Formal analysis, investigation, visualization
E. Leitner: Investigation, methodology
M. Mueller: Funding acquisition, resources, writing - review & editing
D. Mink: Funding acquisition, project administration, writing - review & editing
M. Schuermann: Funding acquisition, project administration, resources, writing - review & editing
K. Gruber: Conceptualization, funding acquisition, project administration, resources, validation, writing - review & editing
H. Pichler: Conceptualization, funding acquisition, project administration, resources, supervision, validation, writing - review & editing
